# Deep Learning With Radiomics for Disease Diagnosis and Treatment: Challenges and Potential

**DOI:** 10.3389/fonc.2022.773840

**Published:** 2022-02-17

**Authors:** Xingping Zhang, Yanchun Zhang, Guijuan Zhang, Xingting Qiu, Wenjun Tan, Xiaoxia Yin, Liefa Liao

**Affiliations:** ^1^ Institute of Advanced Cyberspace Technology, Guangzhou University, Guangzhou, China; ^2^ Department of New Networks, Peng Cheng Laboratory, Shenzhen, China; ^3^ Department of Respiratory Medicine, First Affiliated Hospital of Gannan Medical University, Ganzhou, China; ^4^ Department of Radiology, First Affiliated Hospital of Gannan Medical University, Ganzhou, China; ^5^ Key Laboratory of Intelligent Computing in Medical Image, Ministry of Education, Shenyang, China; ^6^ School of Information Engineering, Jiangxi University of Science and Technology, Ganzhou, China

**Keywords:** radiomics, deep learning, multi-modality images, precision diagnosis and treatment, dosiomics

## Abstract

The high-throughput extraction of quantitative imaging features from medical images for the purpose of radiomic analysis, i.e., radiomics in a broad sense, is a rapidly developing and emerging research field that has been attracting increasing interest, particularly in multimodality and multi-omics studies. In this context, the quantitative analysis of multidimensional data plays an essential role in assessing the spatio-temporal characteristics of different tissues and organs and their microenvironment. Herein, recent developments in this method, including manually defined features, data acquisition and preprocessing, lesion segmentation, feature extraction, feature selection and dimension reduction, statistical analysis, and model construction, are reviewed. In addition, deep learning-based techniques for automatic segmentation and radiomic analysis are being analyzed to address limitations such as rigorous workflow, manual/semi-automatic lesion annotation, and inadequate feature criteria, and multicenter validation. Furthermore, a summary of the current state-of-the-art applications of this technology in disease diagnosis, treatment response, and prognosis prediction from the perspective of radiology images, multimodality images, histopathology images, and three-dimensional dose distribution data, particularly in oncology, is presented. The potential and value of radiomics in diagnostic and therapeutic strategies are also further analyzed, and for the first time, the advances and challenges associated with dosiomics in radiotherapy are summarized, highlighting the latest progress in radiomics. Finally, a robust framework for radiomic analysis is presented and challenges and recommendations for future development are discussed, including but not limited to the factors that affect model stability (medical big data and multitype data and expert knowledge in medical), limitations of data-driven processes (reproducibility and interpretability of studies, different treatment alternatives for various institutions, and prospective researches and clinical trials), and thoughts on future directions (the capability to achieve clinical applications and open platform for radiomics analysis).

## 1 Introduction

In the new era of precision medicine, interest has grown in exploring potential biomarkers embedded in different images. The development of advanced machine and deep learning algorithms has enabled capturing the shape and texture of tissues of concern from multimodality images such as X-ray, computed tomography (CT), magnetic resonance (MR), positron emission tomography (PET), and ultrasound (US). These integrated computational and analytical methods for medical images called radiomics (1, 2) are an emerging field of study.

Intelligent analysis algorithms can be helpful in radiology as an effective aid to physician decision-making in cases of cancer and non-cancer (3–5). In oncology, structural and functional imaging, pathological tissue sections, and combinations provide valuable insights for screening, diagnosis, treatment, and prognostic assessment. Meanwhile, three-dimensional (3D) dose distribution data are considered new “images” and novel predictors of toxicity and prognosis after radiotherapy (RT). The radiomic features extracted from these four images will capture anatomical, anatomical, and functional, pathological, and dose spatial aspects (6, 7), respectively. Evidence has shown that some common imaging characteristics may exist between these different data types, albeit with undoubtedly independent biomarkers and unclear correlations. By integrating the phenotypic properties of medical images and messages extracted from other sources (e.g., pathology and clinical reports recorded) (1, 8, 9), a more comprehensive assessment can be effectively conducted for diagnosing and preparing personalized treatment plans. Radiomics and deep learning have been two rapidly evolving technologies in recent years to achieve this aim, such as the emerging technique of dosiomics, which is an extension of this approach. Their ultimate goal is to create faster and more reliable clinical decision support systems for assisting clinicians, rather than replacing them (3).

Herein, radiomics and deep learning-based radiomics were reviewed, focusing on the types of characteristics, approaches for extraction and selection, statistical analysis, predictive models, and depth feature-based methods. Subsequently, their latest applications and advances in radiology, multimodality, pathology images, and 3D RT dose distribution are summarized and analyzed. Finally, future challenges and recommendations for both techniques were discussed and a robust framework for radiomic analysis was presented. To the best of our knowledge, no systemic reports are available on the progress and challenges associated with dosiomics.

## 2 Materials and Selection Criteria

A literature search was conducted using the Web of Science/PubMed/Medline database by employing the methodological subject terms “Radiomics”, “Deep learning”, and “Dosiomics, “and their “Tumor”, “Cancer”, “Lesion”, “Images”, “Multi-modality”, “Cross-modality”, “Histopathological”, and “H&E” associations to identify relevant studies in November 2021. The inclusion criteria of materials included (a) radiomics, (b) deep learning-based radiomic analysis, (c) uni- or multimodality surveys, (d) single- or multi-omics investigations, and (e) dosiomics. Data were retrieved with a focus on the latest developments and applications of techniques related to radiomics in oncology. First, an initial check regarding potential research that met the inclusion criteria was performed based on the titles and abstracts. Then, an independent and comprehensive review of papers deemed pertinent was conducted. The exclusion criteria included literature from irrelevant fields, published manuscripts in languages other than English, duplicate studies, case reports, and articles that did not include human results.

Moreover, for the newly published applied studies that met the inclusion/exclusion criteria, data from the articles based on full-text analysis and retrieval, including (a) datasets; (b) with or without data enhancement; (c) modality; (d) research subjects; (e) study objectives; (f) methods; (g) relevant features (clinical characteristics, radiomic profiles, and predictive attributes); (h) model building (prediction and validation); (i) results of the model; and (j) conclusions, were extracted and analyzed.

## 3 Radiomics

Radiomics is a cost-effective and non-invasive approach to characterize tissue intensity, shape, and texture by quantifying the imaging phenotype of the region of interest (ROI) (1, 10, 11). Several basic steps are involved, including image acquisition and preprocessing, ROI annotation, feature extraction and selection, and model construction and prediction. Numerous studies have indicated that radiological differences in radiomic signatures can aid in describing tissue heterogeneity. In addition, as demonstrated by several applications, texture characteristics are associated with the genotype of an organism and contribute to the biological interpretation of image phenotypes, i.e., an area of research that is commonly referred to as radiogenomics (12, 13).

### 3.1 Radiomic Feature Classes

The extracted features mainly fall into qualitative (semantic) and quantitative (non-semantic) attributes. Semantic properties (14) are empirical descriptors proposed by radiologists to quantify the lesion phenotype and are usually associated with clinical outcomes. These traits cannot be mathematically expressed but are helpful for clinicians and radiomics studies. For instance, Wu et al. (15) mentioned that CT semantic signatures of partial solid nodules showed correlation with the diagnosis of patients with invasive lung adenocarcinoma.

**Figure 1:**
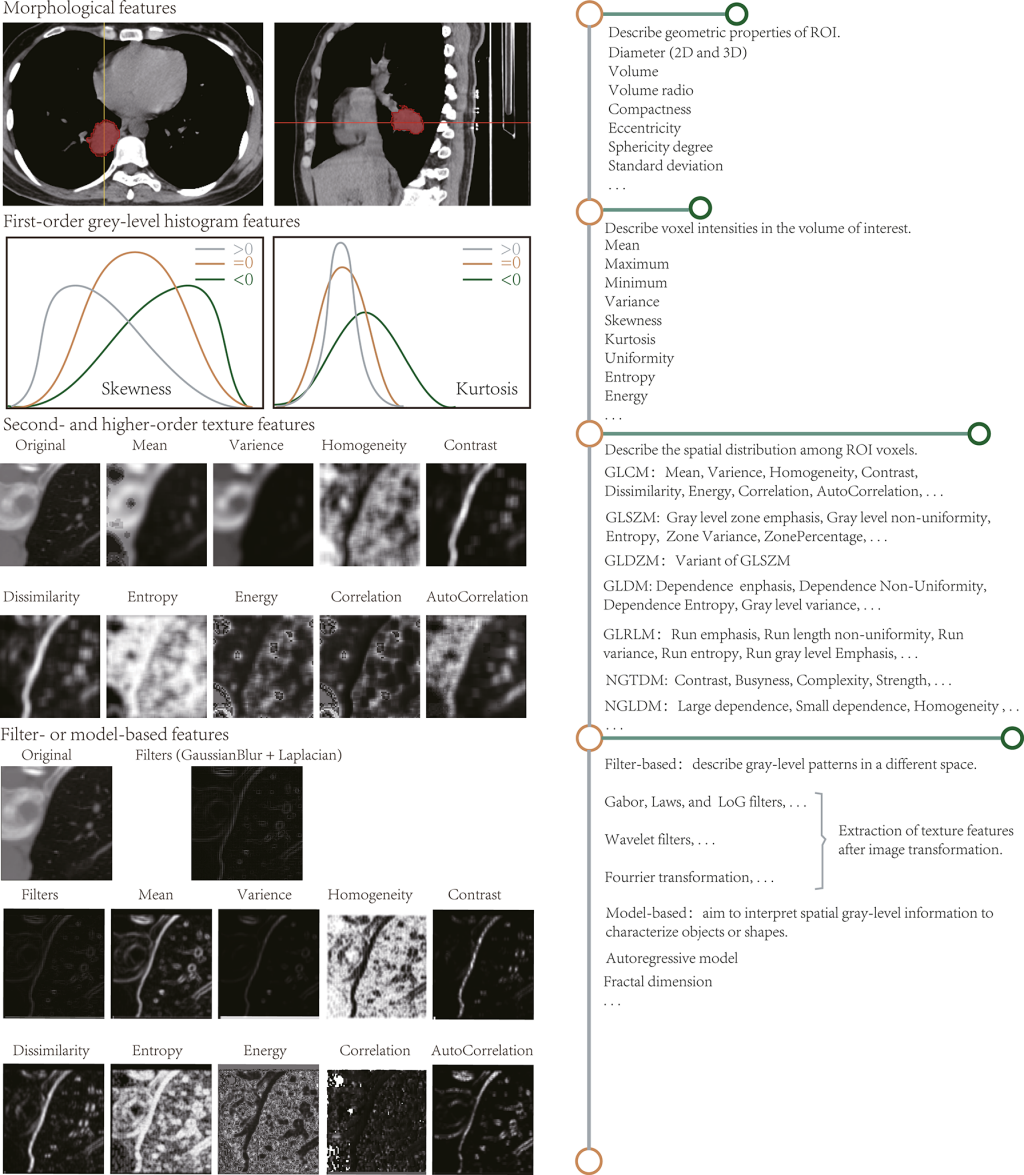
Radiomic features extracted by radiomic analysis tools. The length of the horizontal line on the right describes the approximate number of features.

Non-semantic characteristics can be defined as image representations obtained by building mathematical expressions (2). In radiomic analysis, most quantitative attributes come from voxel information computed from predefined ROI. The first group is the histogram signatures, including the size, shape, and frequency distribution of the lesion voxel intensity. The second set contains the spatial interrelationships of voxel intensities, i.e., texture traits. The properties obtained from the raw or transformed ROI generate the following categories (**Figure 1**).

#### 3.1.1 Morphological Features

Describe the geometric ROI composition. Characteristics that are associated with shape and volume (16, 17), such as two-dimensional (2D) and 3D maximum diameter, effective diameter, maximum axial length, and ratio, volume, maximum surface area, the surface to volume ratio, compactness, eccentricity, sphericity degree, and standard deviation, have already been reviewed. Conceptually, shape properties are simpler and easier to understand than other attributes. For instance, the standard deviation reflects the similarity of the ROI to a circle or sphere, and the sharp edges present the morphological appearance of the lesion area.

#### 3.1.2 First-Order Gray-Level Histogram Features

Reflect the gray-level frequency distribution of voxel intensities and do not contain spatial relationships. Histogram analysis aims to calculate the statistical variables for each voxel on the image such as mean, maximum, minimum, standard deviation, variance, skewness, kurtosis, uniformity, and entropy. Among these variables, skewness and kurtosis describe the shape of the data density distribution and measure the asymmetry and flatness of the data layout curve, respectively. Because histogram-based attributes do not focus on voxel locations and distinguish between spatial distributions, they cannot subsist understood as actual “texture” characteristics.

The grayscale histogram based on single-pixel or single-voxel analysis reveals the homogeneity of images and is known as a summary of first-order statistical information. Histograms of homogeneous and heterogeneous lesions correspond to a narrow and a broad intensity peak, respectively. Based on tumor volume changes in patients with cervical cancer who are treated with RT, Bowen et al. (18) proposed statistical test intensity histogram scores on MR, fluorodeoxyglucose (FDG)-PET/CT, and diffusion-weighted imaging images to describe tumor heterogeneity, such as FDG-PET SUVCoV, dynamic contrast-enhanced-MRI SICoV, and DW-MRI apparent diffusion coefficient (kurtosis). Considerable variation was noted in the apparent diffusion coefficient on DW-MRI in early RT treatment, suggesting that some intensity histogram heterogeneity signatures are concerned with RT response. Virginia et al. (19) identified primary mass entropy as a prognostic indicator of the overall survival in a training set for non-small cell lung cancer (NSCLC), but not reproduced in the validation cohort, thereby raising questions about the results of a small cohort study.

#### 3.1.3 Second- and Higher-Order Texture Features

By evaluating the spatial distribution among voxels, spatial variations in voxel intensity levels can be perceived or measured. Second-order statistical characteristics are obtained by computing the spatial relationships between neighboring voxels such as energy, entropy, uniformity, and contrast. Third- and higher-order texture traits describe the distribution across three or more voxels and assess roughness, busyness, and complexity, among other parameters. Such texture signatures are derived based on matrices that mainly contain the gray-level co-occurrence matrix, gray-level size zone matrix, gray-level distance zone matrix, gray-level dependence matrix, gray-level run-length matrix, neighborhood gray-tone difference matrix, and neighborhood gray-level dependence matrix. They are elucidated in **Supplementary Section 1.2**.

Therefore, texture analysis evaluates the spatial distribution of pixel intensities based on different parent matrices, focusing on the relationship between each voxel and its neighboring regions and emphasizing on local analysis. Conversely, histogram features reflect global properties.

#### 3.1.4 Filter- and Transform-Based Features

Unlike Section Section *Second- and Higher-Order Texture Features*, and higher-order texture, it is calculated after the ROI transformation. Spatial and frequency domains describe the texture information at different scales and are extensively studied on CT and MR images (20, 21). Filters, such as Gabor, Laws’ texture, Gaussian, and Laplacian, are usually applicable for spatial level conversion. Complex linear or radial wavelets are available in the frequency domain, and Fourier can also turn the spatial representation of an image into a frequency mode.

Wavelet features are excellent examples. For instance, the grayscale evolution of an image can be observed better using Haar wavelets (22) after processing the image using a high-pass (focus on image details) and low-pass filter (ignore image details), and image preprocessing and segmentation is also performed adequately using these wavelets.

#### 3.1.5 Model-Based Features

These depict the spatial gray-level shape information, which inscribes geometric complexity from the most complex mathematical models. For instance, the fractal analysis (23) assesses the self-similarity and roughness of distinct dimensions by superimposing various patterns on the image. Such methods generate fractal dimensional profiles that reflect the rate of change between magnification scale or resolution and structural detail, i.e., the self-dependent likelihood of the texture when the image is scaled up.

Most above-mentioned features are neither original nor novel. Texture signatures to quantify image representations and the adoption of filters and digital transformations to decompose signals essentially emerged decades ago. In medical images, the main innovation lies in radiomics, which captures novel biomarkers of tissue lesions. More importantly, it can be combined with other data types, such as metabolism, genetics, and pathology, to identify more valuable phenotypic profiles, promising for comprehensive disease assessment.

### 3.2 Feature Extraction Process

Data collection is the first step in the radiomics workflow. At present, most investigations rely on retrospective data. The impact of image acquisition parameters and reconstruction algorithms needs to be considered while designing the study methodology (**Supplementary Section 1.1**).

#### 3.2.1 Image Segmentation

In radiomics guidelines, delineating the ROI from 2D or 3D images is critical, determining the region to calculate the radiomics features. This step is considerably tedious and challenging, especially in diffuse diseases or in the presence of multiple lesions.

ROI segmentation has not been automated yet. The tumor or lesion tissue gets manually outlined by experienced radiologists (22, 24), which is considered the most straightforward method. However, this process is laborious and susceptible to interobserver variation (IOV), and it should be ensured that at least two or more experts simultaneously observe and reach consensus to minimize IOV. Pavic et al. (25) investigated the effect of IOV for radiomic analysis based on ROI delineated by three experts and found that three distinct tumor types had varying median Dice coefficients (DC), i.e., considerable IOV existed. Many researchers believe that having experts divided ROI is the fundamental truth despite time consumption and variability.

Semi-automatic segmentation algorithms have evolved to maximize timeliness, accuracy, and automaticity in different imaging modalities and lesions. Region growth (26) is a routine procedure in which the operator first selects a seed point. When the neighboring pixel sites have similarities with the core pixel point, they are merged and are allowed to continue to grow outward until no more pixel spots satisfy the condition. Such an approach is suitable for partitioning relatively homogeneous patches and requires an experienced physician to perform contour correction of in homogeneous tissue areas, e.g., non-/sub-solid and nodules involving the blood vessels and pleural surfaces. Threshold class algorithms were generally performed in terms of robustness and accuracy, especially in heterogeneity analysis (27, 28). Furthermore, in the assessment of metabolically active tumor volume, threshold-based approaches focus on the tumor subvolume with the highest uptake, vastly underestimating the true metabolically active tumor volume range (29, 30), which in turn increases the bias of heterogeneity estimation. Some studies have employed the watershed method (31), which connects pixel points with similar spatial locations and grayscale values to form a closed outline. Then, the user selects a rough area, and the algorithm automatically generates a 3D image of the lesion, which is then manually refined on the 3D surface. Yin et al. (32) proposed a novel, fast, and fully automated morphology segmentation algorithm for dividing breast tissue in breast MR images with accuracy and precision that exceeds those of the existing methods. Huang et al. (33) compared and analyzed thresholding-, clustering-, and watershed-based segmentation architectures in breast US images recently and concluded that each technique has benefits and drawbacks.

Software packages are publicly available that support semi- or auto-lesion outlining, mainly from 3D Slicer (34), MITK (35), ITK-SNAP (36), MeVisLab (37), LIFEx (38), ImageJ2 (39), and SmartPaint (40). Parmar et al. (26) compared ROIs obtained by semi-automatic, and five experts depicted layer-by-layer, with intraclass correlation coefficients (ICC) of 0.77 ± 0.17 and 0.85 ± 0.15, indicating higher reproducibility and robustness of radiomic features derived from the ROI outlined by the region growth algorithm. No suitable universal segmentation algorithm is available because of the lack of a gold standard for defining ROI and the possible difficulty in capturing morphological variations and boundary blurring between different lesions. Therefore, performing preprocessing operations is essential to improve the quality of ROI before extracting traits.

#### 3.2.2 Feature Extraction

The following phase is to quantify the attributes at the gray level within the segmented region. Radiomic properties have been described in detail in Section 3.1. Their extraction and analysis are complex, but several open-source packages are already available. The Image Biomarker Explorer is already being tested for feature extraction and modeling cross-modality medical images. Recently, Bettinelli et al. (41) investigated the compliance of Image Biomarker Explorer and showed that preprocessing introduces non-negligible inconsistencies, but the developed Standardized Image Biomarker Explorer complies with the Image Biomarker Standardization Initiative (IBSI) standard. PyRadiomics (10) and Chang Gung Image Texture Analysis (42) can be accessed as plugins to generate quantitative imaging signatures but do not include analysis modules. Mazda (43) and Computational Environment for Radiological Research (44) contain several textural analysis modules such as image import, outlining ROI, image preprocessing, and feature extraction. These are open-source toolkits, and commercial packages, such as RadiomiX (45) and TexRAD (46), are available. They are simple to use but support fixed characteristics and are less scalable. Some in-house programs are also available, e.g., the open-source Matlab-based Quantitative Image Feature Engine (47). The characteristics and functions supported by these available packages are summarized in **Supplementary Table S1**.

These toolkits differ in the level of support for image types and formats, outlining ROI, preprocessing, and modeling, and show inconsistencies in the types and names and the number of features. Therefore, different methods produce various gray-level phenotypes, and validating the same model using different programs is challenging.

However, the IBSI provides a uniform definition for the supported radiomic feature, i.e., the name and number of offers are always constant. The values obtained for the same characteristic are variable under distinct calculation parameters, especially for second- and higher-order texture traits. Therefore, newer information about the segmentation region can become accessible with specific and different computational variables. Although such variability may affect the texture profile robustness, it can aid in optimizing the texture analysis. For instance, checking the compliance of diverse software packages according to IBSI can improve the potential for reproducible validation of the same model (41).

Standards for image preprocessing and features facilitate the construction of reproducible models and potentially accelerate the translation of radiomics methods to clinical applications. In addition, updating multiple existing toolkits to meet the IBSI initiative standards is one way to obtain a common software solution.

### 3.3 Feature Selection and Dimension Reduction

As noted in Section *Feature Extraction Process*, extraction, the number of computed features can often vary from a few hundred to several thousand (e.g., 104, 867, 1108, and 7260 (48–50), respectively), which is frequently more considerable than a much larger size of the study cohort that may continue to increase. Many factors do not aid in outcome prediction (51, 52); as a result, improving the count does not mean that more amount of new and valuable information is available. The non-repeatable, highly correlated, redundant, extremely large, or small variance and outlier traits should be exclusionary. Moreover, the greater the number of traits involved for forecasting or the smaller the patient sample size, the more likely is the overfitting result. Therefore, selecting valuable attributes from the character set is essential to build a prediction model. Several methods for feature selection have been developed, mainly covering the following aspects:

#### 3.3.1 Feature Harmonization

Harmonization techniques eliminate batch effects in high-throughput data, i.e., removing the center-dependent impact of scanner parameter variations for radiomic analysis. ComBat Harmonization is one of the newest and most promising schemes available. Initially, this method proved effective in genomic data and preserved pathophysiology information (53). However, it was soon adopted to solve the center effect problems in radiomics studies. Mahon et al. (54) assessed the capability of the ComBat algorithm on CT images of patients with lung cancer. The percentage of considerably diverging characteristics produced by the 32 imaging protocols was noted to be 0%–2% or was retained at 0%. Additionally, studies based on MR and PET images have employed this technique (55, 56), thereby further demonstrating the possibility to reconcile the radiomic profiles of different imaging modalities. Recently, Da-ano et al. (57) proposed a hybrid version based on a modified B-ComBat and M-ComBat, namely BM-ComBat, to improve the robustness (B) and flexibility (M) of the estimation. All ComBat versions could eliminate the differences in radiomic characteristics between institutions, but the improved ComBat provided the best results. Therefore, the BM-ComBat method is recommended as the preferred choice for model development and validation in a multicenter study.

#### 3.3.2 Removal of the Interobserver Variation Features

If manual or semi-automatic methods are implemented in lesion segmentation, irreproducible or highly variable signatures introduced by IOV require exclusion. ICC is routinely employed to assess inter- and intra-reader agreements. Pavic et al. (25) compared the variability of tumor regions that are manually outlined by three observers on CT images (ICC of >0.8 indicates excellence). They found that IOV has various degrees of influence on the radiomic analysis of diverse tumor. Considering that the ICC calculation relies on the natural variance of the underlying data, testing repeatability alone may be insufficient. Kendall’s W (58) can also evaluate IOV consistency when three or more operators are present, i.e., test–retest analysis is a necessity to maximize the robustness of imaging attributes.

#### 3.3.3 Selecting Relevant Features

A common scheme excludes redundant or irrelevant characteristics. Based on recent studies (59–62), the popular feature selection methods in radiomics are summarized in **Supplementary Table S2**. The previous three types are filtering, wrapping, and embedding. First, the filtering way ranks variables according to their scoring criteria in two manners: univariate and multivariate. The univariate analysis depends on profile relevance to the target variables, whereas the multivariate analysis combines correlations and redundancies. For instance, Relief (63) calculates weights based on the relevance of each property to the outcome, and components with less value than a certain threshold will get removed. The core idea of minimum redundancy maximum relevance (62) is to determine the amount of mutual information between a set of indicators and predictor variables and then select those with the maximum mutual information and minimum redundancy. This filtering operation is typically performed without considering the model and is also an independent process. Secondly, the wrapping pattern prunes unwanted elements in the initial signature set by recursive model training until the best subset is found. Among them, recursive feature elimination (64) is the most frequently employed approach. Finally, the embedding manner incorporates feature selection into the model building process. The least absolute shrinkage and selection operator is an exquisite example of simultaneously generating relevant properties and predictive models (65). Both wrapping and embedding techniques evaluate traits based on the forecast results. The difference is that one is recursive filtering and the other is automatic adjustment of parameters during model learning. Generally, the solution with model involvement has higher accuracy, and filtering focuses on preliminary screening, e.g., some studies employ P-values (<0.05) to detect associated hallmarks (66), and others reduce the number of attributes by the Chi-squared test and Mann–Whitney U test (67).

Apart from the three methods already described, unsupervised techniques are another effective means to reduce data dimensionality (68, 69). Mapping the characteristic set to a lower-dimensional space by linear or non-linear transformation minimizes information loss. Cluster analysis and principal component analysis are representative examples. The first step of cluster analysis is to establish attributes with high intra-cluster redundancy and low inter-cluster correlation and then choose the most representative variables from the different groups to build the model, which can be visualized by clustering heatmaps (70). The principal component analysis targets creating a smaller subset of maximally uncorrelated from the feature set to describe the phenotypic evolution in imaging with as few primary elements as possible (71). Furthermore, this approach does not rely on objective variables (benign or malignant), has no overfitting risk, and is a highly preferred method.

### 3.4 Model Construction and Classification/Predictive

Once the feature selection step is complete, the most promising predictors that remain are directed to model training to evaluate the current research objectives. The target variables can be scalar (survival in time) or categorical (tumor diagnosis and cancer subtype). Depending on the usage level of prior variables (outcomes), the models are classified as supervised, semi-supervised, and unsupervised learning. **Supplementary Table S2** summarizes the feature selection methods and models popularly practiced in radiomics to enhance the readability and comprehensibility of the manuscript.

Supervised learning models are analyzed in conjunction with outcome variables to establish a mathematical representation between the selected characteristics and the target variables, a widely utilized method in radiomic analysis. Support vector machine (SVM) is a commonly employed promising discriminative classification technique and is a typical practice to introduce multiple classification models for profiling to achieve better performance (24, 71–73). For instance, Kim et al. (71) showed that SVM, logistic regression (LR), bagging tree, boosting tree, and dual-channel bidirectional long and short-term memory network performed well for prostate cancer identification on tissue images. Many other supervised classifiers exhibit favorable learning abilities such as the least absolute shrinkage and selection operator-LR (74), multivariate Cox proportional hazards regression models (59), decision trees (75), and random forest (51). A study comparing six feature selection strategies and nine classification measures in a prognostic task for nasopharyngeal carcinoma in which a combination of RF-based hallmark screening with RF classifier was used showed the best performance (76). The machine learning algorithms allowed for easy realization in tools such as R (77), MATLAB (78), and scikit-learn (79), ranging from simple linear regression or LR to more complex SVM or neural networks. The predictive power of the models is excellent; however, their learning process considers the prophecy goals and is prone to overfitting problems, thereby leading to overly optimistic results. Sufficient signatures make forecasting possible in random data even without incorporating objective variables. Therefore, on one hand, more potential traits need to be mined, and on the other hand, more medical data should be made available. Radiomics has developed to a considerable extent in recent years; however, limited annotated data are still a pain point.

In the case of poorly labeled data, unsupervised learning models could serve as an alternative. They utilize the distance metric between samples to calculate the similarity, divide the samples with high similarity into groups, and evaluate the prediction level based on the clustering results. Commonly employed algorithms include k-means (80), fuzzy (81), and consensus clustering (82). A previous study with consensus clustering assessed tumor heterogeneity on CT images of patients with lung, head, and neck cancer, splitting the clustering outcomes into two teams, each with different radiomic characteristics and varying prognoses. This approach, which does not consider target variables, seems more clinically appropriate, but the performance of the model is hardly satisfactory.

A semi-supervised learning model may be a great choice to balance performance and labeled data. The principle is to exploit a large amount of non-annotated data to mine potentially valuable information, combined with a small number of annotated items, and establish a relationship between features and desired output values. The method is relatively common in the deep learning framework, as described in the next section.

Performance evaluation is an essential process after modeling. The predictive power of a model can be quantified in different ways. The most widely adopted metrics for binary discriminant types are the receiver operating characteristic curve, the area under the curve (AUC), specificity, sensitivity, and accuracy (49, 62). In survival analysis and regression tasks, the general assessment measures are the consistency index and the time-based receiver operating characteristic curve (83, 84). Moreover, calibration often works as an indicator with a calibration plot that visualizes the correlation between forecast and actual risk values (85).

The reliability of the findings is another crucial metric that is considered a prerequisite for entry into clinical practice. Internal validation is the first thing to be satisfied by dividing the dataset into a training and testing and validation set, and then training, optimizing, and evaluating the model in the segmented subset. Stratified sampling (69) and random division (86) are commonly performed. The groups, after utilizing stratified partitioning, have similarity distribution but are more tedious than simple random separation. Conversely, the stochastic nature of random splitting leads to uneven data patterns and typically requires multiple separations and reporting of average returns. Cross-validation is the most prevalent scheme. *K*- fold cross-validation divides the samples into k disjoint subsets, where *k – 1* is the training set, and the remaining is the test set (68). The leave-one-out cross-validation method ensures that the value of *k* is equal to the number of examples and only one is used for testing at a time (87). The second is external validation based on patient data from different institutions, which is more reliable because of the difference in distribution of patients in various regions.

At present, no single feature selection method or classifier seems to perform best for different tasks. Therefore, considering ensemble learning and fusing several classifiers may be an effective way to improve model robustness. Additionally, deep learning techniques play an increasingly important role in the medical field and provide a promising direction for radiomics.

## 4 Deep Learning-Based Radiomics

**Figure 2:**
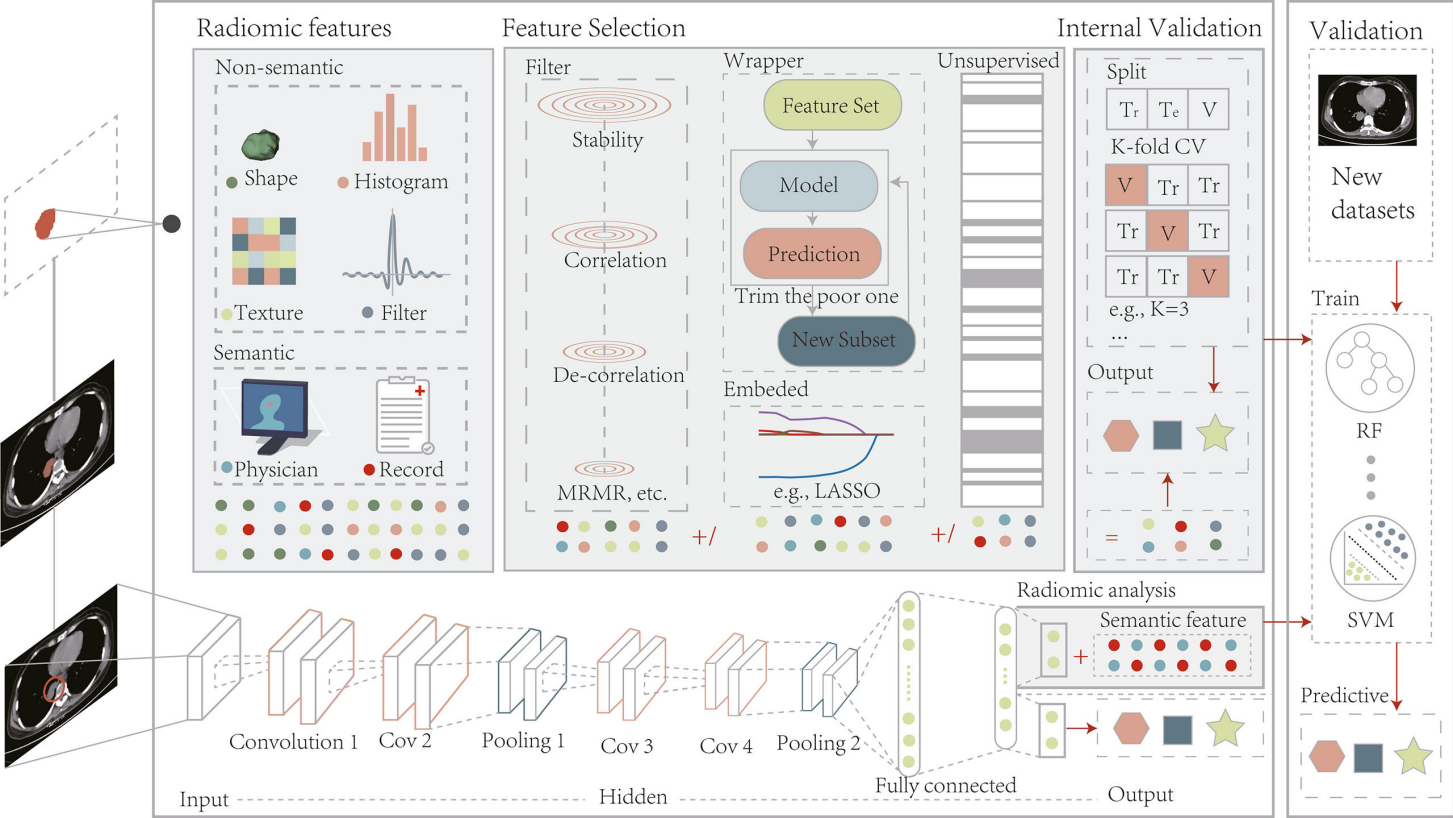
The radiomics pipeline includes two modeling approaches, manually defined and depth features. Modeling pre-defined features involve several basic steps, including image acquisition and pre-processing, ROI segmentation, computation of features, feature filtering, and internal validation. Deep learning picks up features by performing end-to-end training on a coarse region containing the target without a separate feature extraction and selection process. After training is complete, depth features can be combined with semantic ones for radiomic analysis or applied directly to model prediction. The models built by both methods should undergo external validation on a new dataset. Tr, Train; Te, Test; V, Validation; LASSO, least absolute shrinkage and selection operator;.

As described in Section 3.2.1, radiomics can be a valuable tool for accurate diagnosis and treatment planning. However, ROI segmentation requirements hinder development because the process is too cumbersome and dependent on the experience of the operator. Deep learning algorithms are a good alternative to address this problem because they are capable of automatically learning phenotypic features with powerful characterization capabilities without predefined characteristics and human intervention and are considered advanced radiomics (**Figure 2**) (88–90). Considering that deep learning methods are not the focus of this study, we have focused on their application in radiomic analysis. Information on the segmentation algorithms and prediction models of these methods is provided in **Supplementary Sections 1.3, 1.4**.

### 4.1 Deep Learning-Based Lesion Segmentation

As described in Section *Image Segmentation*, segmentation algorithms have demonstrated that they can improve the annotation process of ROI; however, reaching a level of automation is still a challenge. Deep learning methods are a more potentially effective means (**Supplementary Section 1.3**).

Recently, deep learning-based automatic segmentation techniques have been rapidly emerging (91–93). Because multimodality images can provide complementary information, Guo et al. (94) proposed a deep learning framework for automatic selection of gross tumor volume in PET/CT with a DC of 0.73, indicating that the suggested method has a better outlining result than the advanced U-Net (DC 0.71). Tan et al. (91) compared the performance of 12 models based on U-Net, GAN, attention mechanism, and multiscale fusion for separating pulmonary vessels on CT and CT angiography imaging. Results showed that spatial information and multiscale feature maps facilitate algorithm accuracy. Convolutional neural networks (CNN) are already engaged in processing histopathology slices. Xu et al. (92) utilized CNN pre-trained by ImageNet to segment and classify brain tumors and colon cancer and noted that the characteristics extracted by CNN are considerably more effective than those designed by experts. Besides contouring tumors, Amyar et al. (93) developed a multi-task model to auto-screen COVID-19 pneumonia from chest CT images and validated the superiority of the proposed approval (DC >0.88). Automatic ROI generation is beneficial for radiomics studies because it can improve the IOV and labor costs in manual or semi-automatic ways to a considerable extent. However, performing the same segmentation objective with trained models on different datasets usually leads to task failure or not achieving the expected outcomes, which may be the main reason for the limitations of this technique.

As discussed in Section *Feature Selection and Dimension Reduction*, subtle segmentation errors will lead to discrepancies in the extracted radiomic features, which may result in significant measurement bias. Therefore, deep learning-based methods are proven to be very promising; however, validation and correction of the results should not be neglected. Any automated segmentation technique that acts as an outlining tool should undergo careful review and approval of the results by medical experts to ensure the reliability of the study.

Moreover, the study of radiomics remains controversial, where differences in preprocessing, ROI segmentation, feature extraction, feature selection, and the classifier can affect the final performance of the model. Semantic layer segmentation is getting closer to the physician’s visual level with the addition of deep learning algorithms; however, its role in optimizing the overall workflow remains limited.

### 4.2 Depth Feature-Based Radiomics

Deep learning is not a new concept and has been around for decades. The progressive availability of accessible medical data and computing power has given rise to new radiomics that are non-deterministic and non-pre-defined (**Figure 2**) (95, 96). Such deep network architectures rely on the data driven to produce more abstract, richer, general, and robust depth features without expert definition, which perfectly match the medical big data. They can do what medical experts with extensive experience do in many ways, such as identifying image attributes, fusing multiple types of metrics for diagnosis, and generating preliminary diagnostic reports (97–99). Meanwhile, several studies have compared this technology with handcrafted signatures for radiomics and reported the potential of depth characteristics (100–102).

Conceptually, deep learning algorithms are generally broadly classified into generative and discriminative ways (96). They can be used to generate models to guide the different relationships of the input data. The conditional probabilities of various categories are then calculated by utilizing the joint distribution, and finally, the category with the highest probability works as the prediction outcome. Extending this approach to radiomics, which aimed to identify intrinsic features of the phenotype and assess its heterogeneity (tumors), a generative strategy may be more appropriate. The frequently employed models are shown in **Supplementary Section 1.4.1**.

Contrarily, discriminative models do not compute joint distributions but learn the mapping relationship between *x* and *y* directly. The ultimate concern is the output of *y*, given the input of *x*. For instance, if the research question predicts a benign or malignant nature, discriminative learning may be a better choice. **Supplementary Section 1.4.2** is a typical example.

Deep learning algorithms are typically network architectures composed of three or more layers; thus, millions of parameters (weights) need to be estimated, which is computationally intensive and requires datasets of sufficient size for training and parameter tuning. Samples per category require 1000 or more to perform well in a classification task trained from scratch. Approximately, 100 per class possibly achieves a more reasonable result in some data augmentation techniques (103, 104). Furthermore, the potential of transfer learning has been evident in several studies (100, 104). The principle is that common characteristics between source and target data are identified and migrated to new feature space for the training of the target model (105). Xu et al. (106) proposed a pre-training-based model for problems associated with insufficient data, which effectively reduces the overfitting risks. Several researchers worked with only the first few layers of a pre-trained CNN and then retrained the later ones in a new optimization task (107). Thus, transfer learning can first train a rough approximation model for a given task and serve as a basis for modeling a novel task. Therefore, successful examples are rare between medical images, and only a few studies have explored the stability of depth traits.

Deep learning methods demonstrated outperforming the feature engineering-based approaches in many tasks such as detecting lesions (108), predicting mortality (109), and image registration (110). Furthermore, with the increasing amount of electronic data from major medical institutions and the availability of more medical data, deep learning solutions should be the preferred alternative for radiomics research in the coming years. A basic guideline should rule whichever is selected: avoid building a complex model with no significant performance improvement than simple machine learning methods.

Deep learning-based radiomics solutions undoubtedly have tremendous potential for development. However, many challenges need to be addressed to replace traditional radiomics effectively: (a) deep network structures contain millions of parameters and require reliance on massive datasets for efficient training to avoid overfitting; (b) the design and parameter optimization of algorithms are very complex and involve many hyperparameters that require tuning (e.g., number and size of the convolutional kernel, learning rate, and activation function); (c) traditional machine learning models seem to offer more explanatory power than the black-box deep learning approach. The introduction of transfer learning, data augmentation, and visualization techniques and the construction of a diagnostic map of medical knowledge will contribute to resolving these issues. Multi-omics methods that fuse different types of medical data are a promising and novel topic for future research. The next section presents the latest developments in radiomics to assist stakeholders in understanding the applications of clinical, imaging, pathology, and genetic data.

## 5 Some Case Studies and Applications

**Figure 3:**
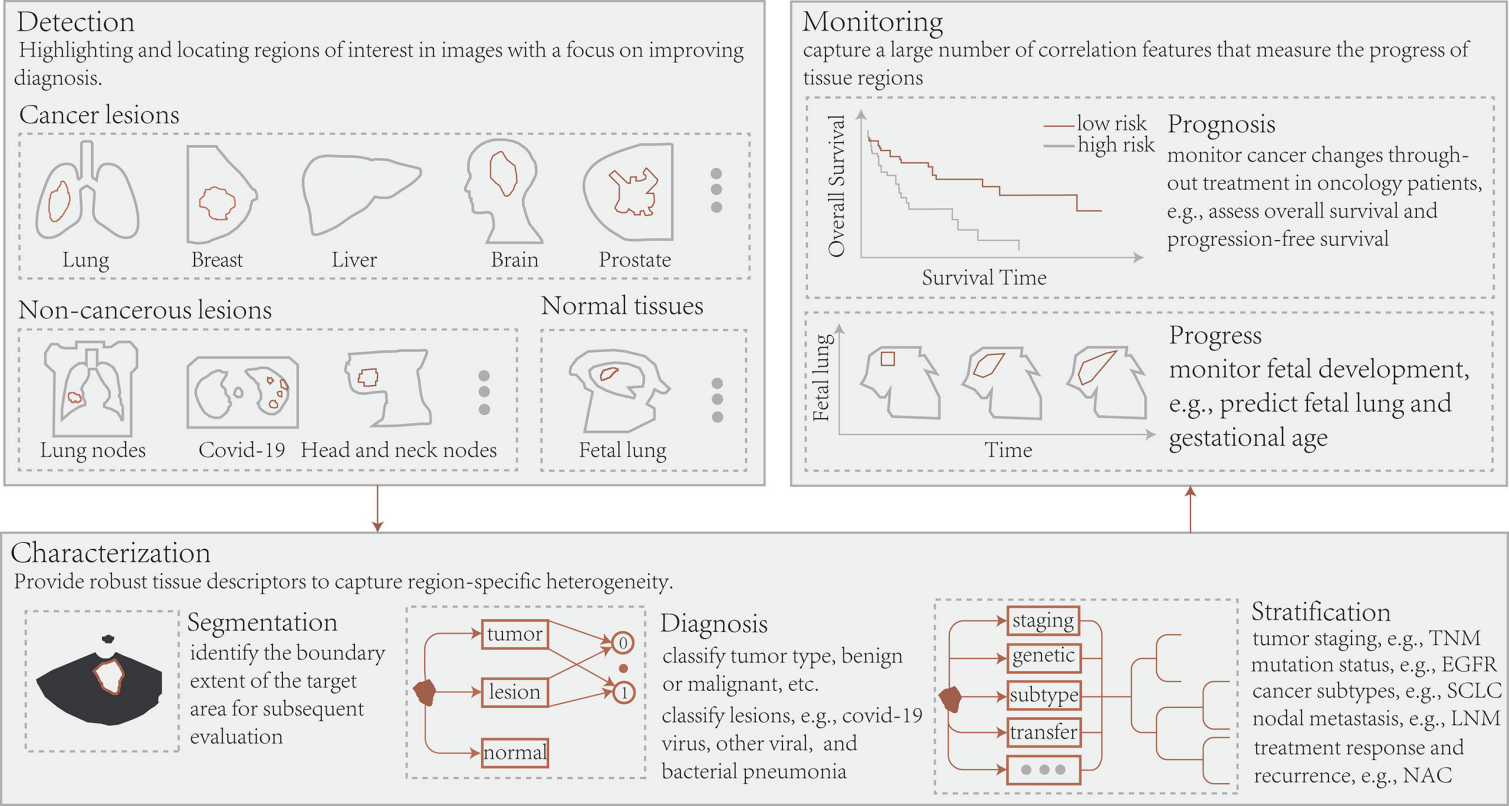
Conceptually, radiomics and deep learning in radiology allow the application of three essential types of image-based clinical tasks: 1) Detection of regions of interest, including cancerous, non-cancerous, and normal tissue; 2) Segmentation of target regions, disease diagnosis, and patient stratification; 3) Treatment response or prognosis and tissue progression. TNM, tumor node metastasis; EGFR, epidermal growth factor receptor; SCLC, small cell lung cancer; LNM, lymph node metastasis; NAC, neoadjuvant chemotherapy.

A growing number of studies demonstrated the value of deep learning with radiomics to achieve personalized medicine. Herein, advances in their application in radiological images, histopathological images, and 3D dose distributions from the perspective of disease diagnosis and treatment have been outlines (**Figure 3**). Additionally, **Supplementary Table S3** summarizes these latest applications to improve the study readability.

### 5.1 Conventional Radiological Images

CT imaging quantifies tissue density and has vast application. Radiomic analysis of images from multiparametric MR and PET/CT by many investigators allows a more comprehensive assessment from different perspectives such as imaging, genetics, and metabolism. Simultaneously, the potential in simple and real-time US images is becoming apparent. It can be subdivided into radiomics, ultrasonics, and metabolomics depending on the imaging modality.

Moreover, with radiomics techniques, multimodality and/or multi-omic studies have become a vital research topic. Multimodality is the extraction of radiomic features from different imaging, whereas multi-omics attempts to establish cross-omics associations between various data types. Simply put, a multilevel fusion framework has been established, mining more valuable characteristics for a comprehensive radiomic analysis. Fusion methods are crucial in diagnosing and treating tumors because they capture richer information.

#### 5.1.1 Diagnosis

##### 5.1.1.1 Single-Modality Analysis

The most common radiomic application in radiology images is to aid in disease diagnosis. Li et al. (104) proposed an attention mechanism-based model with 88.7% and 70.0% classification accuracy for breast density on in-house and public X-ray datasets, respectively, which can effectively reduce manual operations. Kolossvary et al. (111) found that coronary CT angiography features could predict changes in coronary atherosclerosis. Benedetti et al. (112) explored the association between characteristics of contrast-enhanced CT (ceCT) and non-contrast-enhanced (non-ceCT) images and histopathologic profiles and revealed their role in tumor characterization. Pang et al. (103) used TripleGAN to synthesize breast US data to improve mass classification performance, resulting in a semi-supervised model with AUC, sensitivity, and specificity of 90.41%, 87.94%, and 85.86%, respectively. Jiang et al. (113) designed a depth feature approach based on MR images that achieved better results than the conventional radiomics model in distinguishing vessel invasion in patients with cervical cancer. Several researchers have recently compared the discriminatory power of 2D and 3D ROIs. Meng et al. (62) extracted 2D and 3D signatures from CT images of patients with gastric cancer (GC) from four centers to forecast lymph node metastasis and lymphovascular invasion (LVI) and classify *pT4* and other *p^T^* stages. They noted that time-saving 2D annotations showed comparable performance to 3D in describing GC. Xie et al. (114) claimed that 3D non-ceCT attributes outperformed 2D in predicting BRCA1-associated protein 1 (BAP1) status in patients with malignant pleural mesothelioma. Fewer studies reported on 2D and 3D radiomic analysis, and their findings are conflicting and insufficient to generalize to all studies. Besides being employed for oncology diseases, Du et al. (115) utilized the profile of gestational diabetes mellitus, pre-eclampsia, and normal pregnancies to assess fetal lung and gestational age in gestational diabetes mellitus/pre-eclampsia, supporting the research of neonatal respiratory disorders. Salvatore et al. (116) performed a comprehensive evaluation of MR traits in patients with Alzheimer’s and Parkinson’s diseases to investigate their role in neurodegenerative disorders.

Determining the immunophenotype and genotype of a tumor is crucial for treatment decisions in patients with cancer, especially in those with advanced stages. Ligero et al. (117) identified 14 features from the CT imaging of 85 cases with advanced solid tumors to predict the response to anti-PD-1/PD-L1, exposing a potential relationship between radiomic characteristics and tumor immunophenotype. Genotyping by Rossi et al. (48) indicated that their machine learning model showed globally good accuracy in recognizing epidermal growth factor receptor (EGFR) mutations in CT images of patients with NSCLC after data optimization, a finding validated in public (The Cancer Imaging Archive) and external datasets. Agazzi et al. (49) assessed EGFR-positive status and predicted anaplastic lymphoma kinase rearrangements in NSCLC by radiomic signatures, obtaining an accuracy of 81.76%. Bhandari et al. (118) conducted a systematic review of MR image-based studies of lower-grade gliomas (LGGs) and found that radiomic analysis can non-invasively predict isocitrate dehydrogenase and 1p19q mutations and noted that this method could be an alternative to invasive biopsy techniques. Choi et al. (119) distinguished isocitrate dehydrogenase mutation status in gliomas by a fully automated approach combining radiomics and deep learning, obtaining 93.8%, 87.9%, and 78.8% accuracy in-house in the Seoul National University Hospital and The Cancer Imaging Archive datasets, respectively. These discoveries suggest that radiomics performs well in the detection of immunophenotypic and genomic tumors.

##### 5.1.1.2 Multimodality Analysis

Combining diverse images to identify benign or malignant cancers is the main application. Guo et al. (120) utilized an integrated model of profiles of T2-weighted, DW, and ceCT images to predict perineural invasion in rectal cancer. Results showed that the multimodality approach achieved better performance and was considerably better than any of the individual methods in AUC. Khan et al. (121) proposed an automated multimodality brain tumor classification technique that employs deep learning to integrate the best depth signatures extracted from T1, T1CE, T2, and Flair images by VGG16/VGG19 networks. Radiomic signatures are affected by imaging modality and tumor histology; thus, combining different images can provide more potential information and make feature fusion and selection difficult. Wu et al. (122) designed a deep learning method across two modalities and three cancer types to ensure reproducible automatic tumor segmentation and recognition. Zhao et al. (123) developed a multi-stage radiomics framework based on united adversarial learning and achieved 92.94% accuracy in liver tumor segmentation and detection tasks. Unlike typical radiomic schemes, they designed a pyramid module that computed similarity characteristics to extract complementary multimodal signatures and new feature fusion and selection channels to select the best-fused signatures. Several models to differentiate cancer subtypes are to be developed and validated. Alvarez-Jimenez et al. (124) predicted patients with adenocarcinoma and squamous cell carcinoma by WSI and CT-based traits and found essential correlation attributes across scales between pathological sections and CT images to significantly improve the identification of NSCLC subtypes. Giardina et al. (125) assessed the value of profiles of optical coherence tomography, multiphoton microscopy, and line scan Raman microspectroscopy to analyze and identify morpho-molecular metabolic. The accuracy of the suggested model was as high as 88% and 99% for the binary task of identifying gland and adenomas and the multivariate task of distinguishing pituitary adenoma subtypes, respectively. Shiri et al. (66) performed a radiomic analysis of CT, ceCT, and PET images of NSCLC targeting EGFR and Kirsten rat sarcoma virus mutations with 6 feature selection methods and 12 different classifiers for the combination with genomic data and revealed that the stochastic gradient descent model outperformed the best among the 12 methods. Besides tumor-related applications, Zhou et al. (73) employed multimodality signatures of children’s multiparametric MR images and an SVM classifier to differentiate attention-deficit/hyperactivity disorder from normal children with diagnostic AUC and accuracy of 69.8% and64.3%, respectively, a significant improvement over earlier feature fusion and single-modality approaches. These studies demonstrate that multimodality fusion features possess rich and complementary information that allows robust and highly accurate tumor characterization. Recently, Calisto et al. (126) explored the value of multimodality imaging histology techniques in clinical applications and concluded that introducing the complementary diagnostic technique resulted in a significantly increased clinician productivity and improved diagnostic quality from a report on the behavior of 45 physicians from nine different institutions.

#### 5.1.2 Treatment

##### 5.1.2.1 Single-Modality Analysis

Treatment response assessment is of great value in clinical decision-making, particularly in cancer prognosis. FOLFIRI and bevacizumab are first-line treatment options for colorectal cancer (CRC), and multivariate Cox analysis based on CT radiomic features can predict patients with good responses early (127). Chen et al. (128) showed promising results in predicting the objective response to first transarterial chemoembolization by utilizing characteristics of preoperative ceCT images in cases with intermediate-stage hepatocellular carcinoma (HCC). Dissaux et al. (129) revealed that PET/CT profiles can detect recurrence after stereotactic body RT (SBRT) in patients with early-stage NSCLC. Fatima et al. (130) performed radiomic analysis of US images of patients with head and neck squamous cell carcinoma to assess the recurrence status after RT. The best-selected SVM model achieved AUC and accuracy of 75% and 81% at week 1 and 80% and 82% at week 4, respectively. Xiong et al. (131) discovered that MRI traits could anticipate treatment response to neoadjuvant chemotherapy (NAC) in patients with breast cancer. DiCenzo et al. (132) and Quiaoit et al. (72) demonstrated a relationship between radiomic signatures and NAC on US images of patients with breast cancer. Furthermore, Jiang et al. (85) detected complete pathological responses of NAC based on depth features of US imaging. Hu et al. (133) employed deep learning-based radiomics to assess treatment response in patients with esophageal squamous cell carcinoma directly from pretreatment CT images and indicated that the best-performing ResNet50 model, superior to both radiomics and clinical models, could effectively and accurately forecast the response to neoadjuvant chemoradiotherapy for esophageal squamous cell carcinoma.

Additionally, many studies have concentrated on the prognostic aspects. Haider et al. (134) evaluated the ability of quantitative characteristics of PET/CT images in predicting the prognosis of human papillomavirus-associated oropharyngeal squamous cell carcinoma and demonstrated that selected signatures were associated with the local progression of human papillomavirus-associated oropharyngeal squamous cell carcinoma. Zhao et al. (67) discovered that MRI profiles were predictors of intracranial progression-free survival in patients with anaplastic lymphoma kinase-positive NSCLC. Ferreira et al. (135) discovered that F-18 FDG-PET traits could be additionally helpful information for predicting disease-free survival in cervical cancer but would be affected by different PET/CT device parameters. For longitudinal models, Kickingereder et al. (136) noted that the dynamic automatic quantification of tumor volume in space and time utilizing deep learning algorithms for MRI surpassed the response assessment in neuro-oncology in terms of reliability and prediction of overall survival. Crombe et al. (137) demonstrated on the MR images at baseline and after two cycles of chemotherapy that the Delta-radiomics approach could provide valuable information for predicting the early responses to STS patients with receive NAC that the assessment was improved compared with RECIST criteria.

##### 5.1.2.2 Multimodality Analysis

The main concerns in prognosis are treatment response and OS. Recently, two research groups validated the predictive power of radiomics under multiple imaging for pathological and immune responses (138, 139). Joo et al. (138) investigated the potential of multimodality MR characteristics, clinical information of patients with breast cancer in predicting pathologic complete response to NAC, and deep learning models with fused attributes performed best. Yang et al. (139) designed a unified deep learning architecture based on multimodality sequence messages from CT, laboratory data, and baseline clinical metrics to increase the proportion of NSCLC cases benefiting from anti-PD-1/PD-L1 immunotherapy, thereby fusing multidimensional details to distinguish between cohort anti-PD-1/PD-L1 responders and non-responders. The claimed model can be a promising approach to better distinguish patients who will benefit from this compared with typical radiomics. Several studies have validated that fusion characteristics can also be a robust biomarker in other tumors. For instances, Lv et al. (140) performed a combined PET and CT radiomic analysis to anticipate the prognosis of head and neck cancers. Amini et al. (141) established a fusion signature based on F-18 FDG-PET and CT to assess overall survival for improving NSCLC prognosis and found that image-level fusion strategies considerably outperformed approaches based on single-modal images, clinical information, and feature-level fusion. Yan et al. (142) proved that fusion traits generated from multimodality MRI of glioblastoma could strongly predict progression phenotype after treatment. Additionally, some investigators have successfully applied it to surgical treatment and tumor heterogeneity assessment. Mariscotti et al. (143) employed binary LR to analyze the characteristics of four image types to predict preoperative surgical outcomes for breast cancer, and the combined model had a mastectomy rate of 45%, which indicates superior performance over clinical or individual imaging predictors. Moreover, longitudinal studies of multiple imaging from different time points during the treatment offer unique advantages. Peeken et al. (144) utilized changes in MRI radiomic features before and after neoadjuvant therapy to predict the pathological complete response in patients with high-grade soft-tissue sarcomas. The results showed that the established “Delta-radiomics” model achieved better performance and reproducibility than a single time point method. Xu et al. (145) demonstrated that a deep learning approach based on CT imaging of lung cancer at multiple time points before and after treatment significantly correlated model performance with the number of incorporated follow-up images in predicting prognostic endpoints and was comparable to the time-consuming manual methods used for outlining tumor volumes. Multimodality or multi-omics fusion characteristics and delta-radiomic signatures are potential biomarkers for prognostic assessment.

### 5.2 Histopathological Images

The pathological test is the gold standard for cancer diagnosis, and diagnosis efficiency and accuracy are critical for the subsequent treatment and prognostic assessment. Radiomic techniques offer a new approach; however, radiomic analysis of whole slide imaging (WSI) with gigapixels is a challenging task that has become a research hotspot in the field of pathology, which is known as pathomics.

#### 5.2.1 Diagnosis

Evidence supports that the classification and grading of many tumors, such as breast, colorectal, prostate, glioma, and lung cancer, are possible through histopathological images (21, 71, 88, 146, 147). Specifically, Sharma and Mehra (146) evaluated the discriminative power of handcrafted and baseline pathology and depth features in a breast cancer multi-classification problem, with linear SVM and VGG16 networks exhibiting excellent predictive performance. Trivizakis et al. (21) proposed a multiscale texture analysis framework for CRC classification. They obtained an accuracy of 95.3% in the recognition task of eight types of CRC tissue image patches, which is better than the 87.4% obtained in recent studies. Kim et al. (71) classified benign vs. malignant and low-grade vs. high-grade prostate cancer utilizing the five best pathomic signatures. Pei et al. (147) developed a deep neural network model incorporating molecular and cellular characteristics to differentiate LGG and high-grade glioma. This algorithm reportedly outperforms state-of-the-art methods in detecting LGG II and LGG III and performs better in distinguishing LGG from high-grade glioma. Concerning cancer tissue separation in WSI, Li et al. (88) compared 10 deep learning-based multi-model and single-model methods for lung cancer segmentation. The performance of the best methods was close to the observer’s results. These investigations illustrated that radiomics could facilitate pathologists in locating suspicious areas for further analysis of cancerous tissue.

Furthermore, some applications are determined to analyze molecular typing. Chen et al. (148) proposed an automated method to test the most common HCC subtype of liver cancer through the Inception V3 network with a performance approaching that of a pathologist with 5 years of experience. They discovered the ability to predict CTNNB1, FMN2, TPP3, and ZFX4 mutations. The molecular subtype evaluation of bladder cancer by Woerl et al. (90) indicated that deep learning models reached a level similar to pathologists. Recently, Hu et al. (149) designed for the first time a CNN model to directly predict anti-PD-1 responses on hematoxylin and eosin (H&E) images of patients with melanoma and lung cancer, obtaining optimal results and potentially providing a complementary clinical diagnosis in clinical practice. Qu et al. (150) built an attention mechanism-based deep learning algorithm from WSIs of patients with breast cancer to detect six important genetic mutations associated with targeted therapy, revealing a correlation between depth features and molecular typing. Wang et al. (151) obtained similar outcomes employed a ResNet network to anticipate breast cancer’s BRCA mutation status. Farahmand et al. (152) developed an H&E-based deep learning algorithm to determine human EGFR 2 statuses and trastuzumab treatment response in patients with breast cancer with an AUC of 0.81 and 0.80, respectively, independent of the TCGA dataset. They demonstrated power classification within the level of interobserver variability. However, the clinical meaning of these differences is unclear.

#### 5.2.2 Treatment

Considerable interest has been generated in prognostic assessment based on WSIs in cancer prognosis. Arya and Saha (153) established a multimodality deep learning approach for breast cancer survival detection by combining genomic data, WSIs, and clinical details. The proposed sigmoid gated attention CNN as a feature extraction algorithm and RF as a classifier resulted in considerably better prediction performance than existing methods. Yamashita et al. (7) reported that a deep learning model could automatically learn pathological characteristics associated with microsatellite instability from H&E-stained WSI of patients with CRC, and it recognized microsatellite instability at the level of five gastrointestinal pathologists. Klein et al. (154) demonstrated that deep learning algorithms could directly detect human papillomavirus association in oropharyngeal squamous cell carcinomas from H&E-stained sections to identify patients with favorable prognoses. Wang et al. (155) utilized the depth characteristics of lymph node histopathology images to anticipate GC prognosis and concluded that the tumor area to metastatic lymph node ratio was a clinical indicator of improved prognostic staging. Histological and cellular morphological signatures can provide valuable insights into survival; however, tumor risk stratification shows a considerable association with survival. The established tumor risk score based on WSIs divides patients with HCC into five groups with entirely different prognoses, providing a novel prognostic phenotype for HCC risk stratification (156). Wulczyn et al. (84) developed a deep learning system for 10 cancers to stratify patients with tumors across stages. The deep learning system demonstrated a 3.7% absolute improvement in predicting survival of patients with cancer compared with a baseline clinical staging model. Some studies recognized the potential of histological profiles of the cancer cell microenvironment as a prognostic biomarker, and the combination with genomic data is a promising avenue for improving survival outcomes.

### 5.3 3D Radiotherapy Dose Distribution (Images)

RT is the primary anticancer therapy for patients with cancer, and its applicability rate in the cancer population is close to 50% (6). Radiomic analysis of 3D information on dose distribution in radiation treatment plans utilizing a radiomics framework, known as dosiomics (6), is a new field of radiomics research that has emerged in recent years. Contrary to conventional models based on the dose-volume histogram and normal tissue complication probability, radiomic analysis techniques provide a new approach to predict treatment-related toxicity and prognosis by incorporating spatial and statistical data in 3D dose distribution.

#### 5.3.1 Radiotherapy Toxicity

This approach initially arose in the task of predicting gastrointestinal and genitourinary toxicity after RT for prostate cancer, and the findings revealed that dosiomic features containing spatial relationships between voxel doses improved predictive performance (157). Meanwhile, several researchers have described the potential value in xerostomia after RT for patients with head and neck cancer and radiation pneumonitis (RP) after volume-modifying arc therapy (VMAT) for patients with NSCLC (158, 159). Recently, dosiomic analysis has gained momentum in assessing side effects and prognosis after RT. Adachi et al. (160) developed the dose-volume indices (DVIs) and dosiomics and hybrid (DVIs + dosiomics) models to analyze RP after SBRT in a retrospective NSCLC cohort at three institutions. The dosiomics (ROC–AUC, 0.837 ± 0.054) and hybrid (ROC–AUC, 0.846 ± 0.049) approaches outperformed the DVI (ROC–AUC, 0.660 ± 0.054) approach, indicating that texture-based dosiomic attributes can independently prognosticate RP. Lee et al. (161) utilized a multi-view model based on radiomics and dosiomics to divine early weight loss in lung cancer RT and indicated that radiomics and dosiomics signatures (AUC, 0.710) had a significantly higher predictive power than dose-volume histogram and/or clinical parameters (AUC, [0.534–0.675]) and that dosiomic characteristics were more critical than radiomic profiles. Additionally, a recent study validated the feasibility of applying CNN for RP forecast in patients with NSCLC undergoing VMAT (162). In this research, the CNN model, as compared with dosimetric, normal tissue complication probability, and dosiomics ways, respectively, and the outcomes revealed that methods with deep dose distribution characteristics displayed the best predictive performance (AUC, 0.842 vs. 0.676, 0.744, and 0.782).

#### 5.3.2 Radiotherapy Prognosis

Several recent studies have illustrated that dosiomics is also applicable in evaluating prognosis after radiotherapies, such as locoregional recurrences (LR) (163), biochemical recurrence (BCR) (164), and local control (LC) (165). An LR study in intensity-modulated RT (IMRT) for neck tumors indicated that the combined model based on features of CT, PET, and 3D dose distribution maps performed better than radiomics alone, suggesting that dosiomic characteristics are associated with LR and have prognostic value (163). A dosiomics approach based on prostate, clinical target volume, and planning target volume is a more powerful tool than the classical model containing clinical variables, dosimetric parameters, and radiomic profiles to distinguish between high- and low-risk cases in terms of the risk for BCR in patients with prostate cancer treated with IMRT (164). Notably, the dosiomic features are not more powerful than the clinical parameters, but the combination of these two attributes substantially improves performance. Buizza et al. (165) extended this method to rare tumors to assess LC after carbon-ion RT (CIRT) for skull-based chordomas (SBC) and noted that dosiomic signatures were the most promising traits compared with clinical variables, the profile of CT and MR, with comparable radiomic and clinical model capabilities. The studies by Murakami et al. (164) and Buizza et al. (165) yielded different conclusions when assessing the predictive power of dosiomic profiles and clinical factors, which may be associated with variations in radiation treatment regimens. Additionally, for the forecasting of gamma passing rate values in VMAT treatment regimens, Hirashima et al. (166) demonstrated that plan and dosiomic traits are potent factors in classifying and predicting the risk of BCR in patients with prostate cancer by comparing plan complexity, dosimetric, and combined models of both for eight diseases. Such findings support the role of dosiomic signatures as a new indicator to evaluate the quality of treatment schemes.

## 6 Discussion

Advances in deep learning with radiomics in radiology have been witnessed in recent years. Their potential to tap into underlying phenotypes has been revealed, i.e., the ability to capture unique imaging features at levels beyond the reach of the naked eye. This technology has thus become a beneficial tool for clinical tasks such as accurate diagnosis, treatment response, and prognostic assessment. Next, the potential and value of this technology from diagnostic and treatment strategies were analyzed and the advances and challenges of dosiomics in RT were independently discussed. Several aspects of the radiomics pipeline that can be improved and will propose a new and robust framework for radiomic analysis were identified to optimize existing workflows. Finally, factors that affect model stability and data-driven process limitations were discussed and unique insights into future challenges and recommendations were provided.

### 6.1 Analysis of the Application of Radiomics

Structural images serve to visualize and assess the internal structure of anatomical regions in radiology, and functional imaging reflects the anatomical and metabolic information of tissues and organs. However, histopathological sections can identify heterogeneity at the cellular level. Underlying biomarkers independent of these three images provide valuable information on tumor diagnosis, staging and stratification, and treatment decisions. Thus, structural and functional radiomic features have the potential to decode many physiopathological architecture descriptors at the microscopic scale, creating opportunities for reverse inference from phenotype to genotype.

#### 6.1.1 Potential in Diagnosis

##### 6.1.1.1 Differentiation and Localization of Cancerous Lesions

Phenotypic information that is difficult to observe visually can detect/diagnose cancer or automatically outline carcinoma lesions. Deep learning-based radiomics-driven multi-classification methods for breast cancer typically employ supervised models based on transfer learning (146). Multiscale texture characteristics of WSIs have demonstrated the best performance in recent studies in terms of the CRC tissue region differentiation (21). Khan et al. (121) used an extreme learning machine model combining transfer learning and feature fusion to classify voxels from multimodality MRI of patients with brain tumors automatically. Moreover, cross-modality, uni- or multi-modal, and united adversarial learning-based approaches were employed for lesion segmentation in multiple cancers (lung, breast, and brain) (122), lung cancer (88), and liver tumors (123), respectively.

##### 6.1.1.2 Histopathological Evaluation and Tumor Stratification

Radiomics is a promising technique for revolutionizing the traditional macroscopic variable approach to tumor characterization, replacing the classical cancer features. In the relationship between tumor phenotypes and pathological characteristics, employing the radiomic signatures of ceCT and non-ceCT, WSIs, and pathology and CT can identify pathological biomarkers of pancreatic neuroendocrine tumors (112), diagnose and grade prostate cancer (71), and recognize NSCLC subtypes (apparent diffusion coefficient and squamous cell carcinoma) (124), respectively. The accuracy of the radiomic model based on depth characteristics of WSIs in predicting glioma grading (LGG and high-grade glioma) (147), liver cancer subtypes (148), and molecular subtypes of bladder cancer (90) has reached the level that can be assessed by pathologists. Multimodality spectral imaging (optical coherence tomography, malignant pleural mesothelioma, and LSRM) for morphology-molecular metabolism analytics has distinguished the pituitary from tumor and classified pituitary adenoma subtypes (125).

##### 6.1.1.3 Tumor Heterogeneity Characterization

Combining dynamic contrast-enhanced-T1 and T2*-weighted imaging MRI features to discriminate vascular invasion in patients with cervical cancer can reveal tumor heterogeneity (113). A multicenter-based CT radiomic analysis focused on characterizing GC (62), i.e., by predicting lymph node metastasis, LVI, and T-stage to quantify tumor progression. Additionally, the integrated signatures of multimodality (T2*-weighted imagings, diffusion-weighted imagings, and ceCT) can predict perineural invasion in rectal cancer better (120). Radiomics yields diversity metrics to quantify tumor habitat and provide traction to establish relationships between underlying molecular alterations and clinical outcomes.

##### 6.1.1.4 Tumor Genotype

Several studies demonstrated the correlation between tumor phenotype and genomic features. For NSCLC, signatures of CT, fusion characteristics of ceCT, and PET forecasted EGFR mutations and anaplastic lymphoma kinase rearrangements, EGFR and Kirsten rat sarcoma virus positivity (48, 49, 66), respectively. Preoperative radiomic analysis of MR and CT images was successfully applied to differentiate isocitrate dehydrogenase and 1p19q mutations in glioma and BAP1 mutation in malignant pleural mesothelioma (114, 118, 119). In breast cancer, deep learning-based radiomics was successfully implemented to identify BRCA, and six different types of positive statuses from WSIs correlated with targeted therapy (150, 151). Many investigations suggested an association between radiomics and genomics; however, few preclinical reports have confirmed a noteworthy relationship.

##### 6.1.1.5 Clinical Variables and Phenotypic Characteristics

The traits derived from US, X-ray, and coronary CT angiography were associated with clinical variables related to disease diagnosis and progression. These parameters include breast masses, breast density, and risk factors for coronary artery disease (103, 104, 111). This finding provides an avenue for early screening and progression assessment of disease.

##### 6.1.1.6 Non-Tumor Diseases

Recent findings that indicate the benefit of radiomic features in neonatal respiratory disease (115), Alzheimer’s and Parkinson’s disease (116), and attention-deficit/hyperactivity disorder (73) suggest that radiomics is also effective in non-oncology cases.

#### 6.1.2 Values in Treatment Strategies

##### 6.1.2.1 Local Recurrence and Response

Phenotypic attributes of medical images have value in dividing local recurrence and treatment response preoperatively. Response to NAC is evaluable based on MR and US imaging of patients with breast cancer (85, 131, 138) and CT imaging of cases with esophageal squamous cell carcinoma (133). Several investigators have combined the radiomic properties of PET/CT with independent clinical and therapeutic parameters to assess local recurrence in patients with NSCLC after SBRT treatment (129).

##### 6.1.2.2 Distant Metastasis

The CT and PET/CT image-based radiomics model can determine the risk of distant metastasis in NSCLC cases treated with SBRT (4, 167). CT radiomic signatures at baseline and 2 months after FOLFIRI and bevacizumab chemotherapy predicted early adverse outcomes in those with metastatic CRC (127). More aggressive tumors may exhibit diverse morphological patterns in the peri-cancerous region; therefore, radiomic analysis of the peri-tumor space contributes to providing potential insights into distant metastasis.

##### 6.1.2.3 Survival Assessment

Fusion characteristics evaluated from multimodality data (genomic data, WSIs, and clinical factors), 18F-FDG-PET and CT, and RFS and PET/CT served to estimate the survival rates of patients with breast (153), NSCLC (141), and head and neck cancers (140), respectively. Staging and stratification of 10 cancer cases using a depth feature-based approach revealed considerably higher survival rates (84). Generally, multimodality and/or multimodal radiomics models have superior survival prediction capabilities than single-modality or single-modal radiomics models.

##### 6.1.2.4 Molecular Targeted Therapy

Overexpression of oncogenes in many tumors benefits from molecular targeted therapies such as EGFR tyrosine kinase inhibitor. Evidence suggests that changes in CT radiomic features extracted before and after treatment could distinguish between NSCLC cases that benefit from gefitinib treatment (4).

##### 6.1.2.5 Immunotherapy

Cancer immunotherapy, which is being strongly developed, is a promising treatment modality, but only if patients who respond to it are selected. Radiomics has successfully adapted to diverse immune phenotypes. Radiomic traits extracted from CT imaging of patients with solid tumors (117), H&E images of melanoma and lung cancer (149), and WSIs of patients with breast cancer (152) can determine the response to anti-PD-1/PD-L1 and anti-PD-1 and trastuzumab, respectively.

##### 6.1.2.6 Side Effects and Prognosis

Radiomics methods can assist in early post-treatment side effect assessment such as radiation-induced lung injury. Reports of lung injuries in patients with lung cancer derived from changes in CT radiomic profiles before surgery and after SBRT treatment showed considerable correlation with expert scores and indicated associations with dose and fractionation. Additionally, characteristics extracted from preoperative H&E images could act as independent factors to assess treatment response and prognosis in patients with colorectal, oropharyngeal squamous cell, gastric, and breast cancers (7, 143, 154, 155).

##### 6.1.2.7 Recurrence or Progression

Studies that recognize tumor recurrence in follow-up images have gradually become apparent. US-based radiomic features can identify recurrence risk in patients with head-neck squamous cell carcinoma treated with RT (130). Glioblastoma multiforme has a poor prognosis and inevitably recurs or progresses. The fusion characteristics of pretreatment multimodality MRI are an important prognostic factor for glioblastoma multiforme progression (142). The delta-radiomic signatures can precisely reflect radiation-induced biological changes.

##### 6.1.2.8 Other Treatments

While performing transarterial chemoembolization in patients with intermediate to advanced HCC (128), ceCT images have variable radiomic signatures and can distinguish between cases with objective responses.

### 6.2 Developments and Challenges in Dosiomics

Dosiomics is the latest development in radiomics and offers new opportunities for establishing more informative models of RT outcomes. This approach has demonstrated prognostic value in patients with different types of tumors and various RT techniques, including weight loss after RT in lung cancer (161), RP after VMAT or SBRT in NSCLC (159, 160, 162), xerostomia after RT, and LR after IMRT in head and neck cancer (158, 163), gastrointestinal, and genitourinary toxicity after RT and BCR after IMRT in prostate cancer (157, 164), LC after CIRT in SBC (165), and prediction of gamma passing rate (166); however, relevant studies remain relatively sparse. Therefore, these results should be considered cautiously, with necessary additional investigations to elucidate their application and potential value in the field of radiation therapy. This technique is undoubtedly suitable for predicting any RT outcome, whether positive (survival and control) or negative (normal tissue injury and complications).

Several aspects in further research require attention. (a) Given their retrospective nature and relatively small sample sizes, reported results must be analyzed and validated in the context of data from multiple institutions. Multicenter studies may include biases related to treatment schemes such as each institution’s protocol policies and dose limits. (b) Dosiomics is similarly subject to reproducibility issues because dose calculation algorithms, grid sizes, planners, and treatment regimens can lead to variations in DVI values and dosiomic characteristics. Recently, Placidi et al. (168) evaluated the correlation between dosiomic properties and clinical outcomes by employing various dose calculation algorithms, 30 distinct dose distributions, and 2 grid resolutions at 8 centers to determine the sensitivity characteristics when there is a change in the dose distribution. This study favorably supports a multicenter investigation of this method, although only dosiomic signatures were considered, without including all possible RT techniques and excluding different feature extraction algorithms. (c) This approach currently focuses on a traditional radiomics framework, which may not fully reflect the unique attributes of a given RT. However, combining handcrafted traits with depth features from deep learning is expected to further improve the performance of predictive models. (d) Till date, no investigations have systematically explored how this technology can address the specific challenges of classical dose-volume histogram and normal tissue complication probability modeling, namely category imbalance due to low morbidity, varying follow-up times, diverse treatment regimens, and heterogeneity and noisy data. Similar work has been put forth in radiomics, and such analysis is missing in dosiomics studies.

### 6.3 Robust Radiomic Analysis Framework

**Figure 4:**
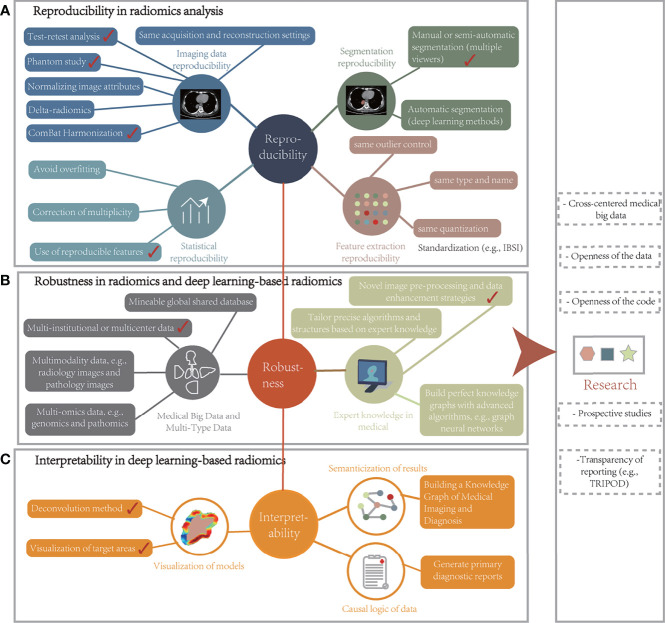
Robust radiomic analysis framework. **(A)** Reproducibility in radiomics analysis can be obtained in terms of imaging data reproducibility, segmentation reproducibility, feature extraction reproducibility, statistical analysis reproducibility, and research reproducibility. **(B)** The robustness of radiomics and deep learning-based radiomics models could be improved in medical big data, multiple types of data, and medical expert knowledge. **(C)** Interpretability in deep learning-based radiomics can be enhanced concerning the visualization of models, semanticization of results, and causal logic of data. IBSI, Image Biomarker Standardization Initiative.

Section *Radiomics* and Section *Deep Learning-Based Radiomics* learning radiomics highlighted the radiomics pipeline in a randomized dichotomous state (**Figure 2**). The traditional approach is to quantitatively extract predefined and handcrafted radiomic features from manually/semi-automatically segmented ROIs for model construction. Owing to the absence of uniform standards, reproducibility and verifiability limitations are often experienced while using these developed models. Therefore, a robust framework for radiomic analysis was proposed (**Figures 4A, B**). First, using test–retest analysis, ComBat harmonization, and phantom study is necessary to reduce the influence of scan acquisition equipment and reconstruction parameters for image data. Second, at least two experts should have reviewed the selected ROIs. Other process counterparts are also involved such as feature extraction, statistical modeling, and the study itself. Thus, ensuring maximum repetition of each step in the workflow is possible, which facilitates the standardization of the entire process.

Moreover, ROI outlining is an important bottleneck because it relies on multiple medical experts with extensive experience who are already overwhelmed by clinical work (14). This problem is expected to be resolved as deep learning algorithms become more prevalent in delineating tumors, non-cancerous lesions, or other structures. Automatically segmented ROIs are fed into the same pipeline, which not only reduces labor costs but also minimizes the effect of IOV. Another approach is to employ the entire image or a coarse target region as input to detect imaging biomarkers. This method typically utilizes deep neural networks, which automatically learn and extract characteristics and obtain more signatures than any manually defined feature algorithm. However, the impact of changes in the way ROIs come from is still limited compared with a rigorous workflow.

The second is deep learning-based radiomics (**Figure 2**), which has shown an apparent growing trend in the development of radiomic analysis. It can learn notable depth features from images without prior manual definition. The advantage of this working strategy is the fully automated classification/prediction process, where the extracted depth characteristics are associated with the expected results. However, deep learning algorithms are not without limitations. The entire process of feature learning and task execution takes place in deep network architecture; thus, many training samples are required to build a satisfactorily robust model. Therefore, purposes, such as disease diagnosis and prognosis prediction, usually require training on thousands of single-class cases, with the available standardized medical image data being relatively less. For instance, in this study, except for large classification tasks (identifying benign and malignant lung cancer), the vast majority of studies have single sample sizes between a few tens and a hundred. At present, many investigations have tried various data augmentation and transfer learning techniques to overcome this weakness. Another issue is the black box problem associated with deep learning. Even the designers/algorithm architects of the researches are unsure about how to select the most relevant depth traits for prediction. Regardless of the accuracy, clinicians are often skeptical of such unexplained outcomes. Therefore, an interpretability framework applicable to depth signatures was proposed to improve the clinical interpretability of studies in visualizing models (**Figures 4B, C**), establishing causal data logic, and generating semantic reports. In the following subsections, we will discuss the factors that affect the robustness of the model.

#### 6.3.1 Medical Big Data and Multitype Data

The future of radiomics is still thirsty for data. First, a reliable conclusion requires a sufficient sample size for training and validation, especially for deep learning methods. Moreover, there is a higher demand for the standardization of data. However, owing to ownership and protection factors, the data are scattered in different medical institutions or research centers worldwide, and researchers do not have easy access to them. Federated learning (169) enables data conversion from multiple centers into mineable shared data while preserving privacy constraints. Simultaneously, the increased type and number of samples and the raised layers and resolution of image scanners make the collection process extremely time-consuming. Therefore, despite its higher storage and labor costs, building a shared database from across the globe is essential. The Cancer Imaging Archive is an important example, and many investigators utilize this dataset to train and validate newly developed models (60, 156, 170). The Reference Image Database to Evaluate Therapy Response is another publicly available data integration project consisting of 31 sets of repeated CT scans at 15-min intervals that can be feature screened by test–retest analysis (171). With such multicenter clinical big data, future researchers working closely with clinicians can conceivably improve the clinical applicability of study results. Noting that early radiomic analysis mainly used semantic and medical expert-defined features with clinical significance is important. Now, the focus of this field has moved to predictive performance improvement with a trend toward high-throughput agnostic analysis. The disconnect with biological interpretation will inherently limit the translation of research results to clinical applications. Overall, reintroducing biological significance into radiomics through emerging approaches, such as genomics, pathomics, and proteomics is necessary.

Conversely, several studies have proved the potential value of complementary information from various modalities or different omics data (5, 172, 173). Moreover, medical images are not isolated assessment metrics, and many factors can influence the disease measurements. For instance, for patients with cancer, molecular tumor biomarkers (blood reporting characteristics), lifestyle habits, socioeconomic status, and even social networks could impact the final treatment outcome. There is a growing awareness that data sources have diversified to include wearable devices, smartphones, social networks, unstructured electronic medical records, and input from other intelligent methods. However, deep learning algorithms are well suited for fusing diverse data streams. Thus, this approach promises to enhance the potential of radiomics techniques in all aspects of radiology.

#### 6.3.2 Expert Knowledge in Medical

Advances in methodology can continuously improve model performance for medical image analysis; however, a surprising conclusion that sophisticated algorithms and precise structures are not decisive factors for building a great solution was reached. Many researchers utilized the same analysis method/network structure when assessing tumor heterogeneity but showed different results. An easily overlooked critical aspect is that expert knowledge in medical of a specific task is more beneficial than the algorithm itself. Studies that achieve outstanding outcomes in utilizing radiomics techniques are often unique in ways other than the approach such as novel image preprocessing and data enhancement strategies or unique network structures that incorporate clinical knowledge. The best-performing model in the breast US-based mass classification task showed considerable improvement after adding preprocessing and data enhancement, rather than changing the network structure (AUC from 88.72% to 90.41%) (103). Furthermore, establishing a strong link between medical images and expert knowledge to form a diagnosis with a causal relationship can significantly increase the study’s credibility. Although graph neural networks have become an effective tool for building well-established knowledge graphs, it is disappointing that no clear way exists to achieve promising performance.

The following discusses the challenges and recommendations for implementing these two radiomics frameworks.

### 6.4 Limitations and Suggestions for Data-Driven Processes

#### 6.4.1 Methods of Reproducibility Studies

Most studies aimed to develop a predictive or prognostic model with high accuracy and efficiency. Ideally, large enough datasets can train and test the new methods and tools developed while using a completely new open dataset for evaluation, which may be the standard for future researches. However, in multicenter datasets, some phenotype variations may not reflect the actual biomarkers of the tissue owing to the image acquisition and reconstruction algorithms, resulting in limited model performance or biased results (174). Therefore, particular attention or exclusion is essential for those features that are more influential, such as kVp, mAs, signal-to-noise ratio (SNR), and thick layers for CT; sequence settings (type, many other sequence factors), temporal parameters (echo, repetition, and relaxation) for MR; and spatial resolution for PET. Additionally, a comprehensive analysis of the reproducibility and stability of quantitative characteristics of X-ray, US, and histopathological images is unavailable. The recent efforts of IBSI in pursuing standardization of high-throughput signatures provide a very informative direction. Another critical way is the Radiology Quality Score (175), which can help assess the robustness of radiomics methods. Pointing out that the Radiology Quality Score primarily guides the workflow of investigations and does not reveal the overall quality of approaches is essential.

For the reproducibility risk of multicenter, many researchers have explored different approaches such as improving SNR, resampling, filtering, and super-resolution reconstruction. Park et al. (23) developed a way for SNR correction based on images with nine different CT scan parameters and found that optimizing SNR is a factor in improving the assessment of tumor heterogeneity. Ibrahim et al. (176) noted a considerable improvement in 42 features after resampling data from 8 different CT machine scanners from 3 different manufacturers. Mali et al. (177) demonstrated that the correction method of resampling and Butterworth low-pass filtering effectively reduced the variation in radiomic characteristics owing to voxel size differences and other CT acquisition variables. Information might be lost during preprocessing and standardizing the CT scan protocol before image reconstruction is best. Tan et al. (178) proposed a preprocessing approach for super-resolution reconstruction of CT imaging that minimizes the effect of layer thickness on the extracted traits to improve the image quality to enhance the vertical resolution, i.e., to strengthen the spatial information in the coronal and sagittal planes. Orlhac et al. (179) could modify the variation in the values of the signatures of CT imaging in a better manner with different thickness layers (1.25 mm, 2.5 mm, and 5 mm) with the ComBat compensation method.

#### 6.4.2 Reproducibility of Different Studies

Reproducibility is a common problem in parameter-sensitive imaging, such that variations in grayscale values in CT and PET images can lead to differences in characteristics and affect prediction results. Some works have addressed the impact of various acquisition parameters; however, absence of reproducibility remains the most critical issue in radiomic analysis, limiting the feasibility of their clinical implementation. Different surveys have developed specific models based on a particular software, making outcome comparison or replication difficult. If specific standardization and validation pathways are in place, the utility of models in clinical trials can be determined. The following aspects can be considered to incorporate radiomics into clinical tests: first, by disclosing the details of each step in the study process to facilitate reproducibility and comparison with other research and meta-analyses, and second, the model must be trained and tested on a sufficiently large data set and its efficiency validated relative to existing models (from other studies) with statistical methods. All methodological details, clinical information reports, final models, and study-related codes should be publicly accessible for optimal reproducibility gains and further independent testing. Finally, pre-validated characterizations may serve as primary or secondary endpoints for inclusion in clinical trials. In an “exploratory endpoint”, an additional test can subsist to identify the most promising signature. Such data-driven biomarkers are indistinguishable from quantitative imaging biomarkers and can further facilitate personalized tumor treatments.

#### 6.4.3 Different Treatment Options for Various Institutions

Multicenter validation is intrinsic to achieving standardization, and multi-institutional studies are subject to imaging modalities, acquisition strategies, and quality assurance devices, which will result in biased results. A recent investigation indicated that dosiomics models outperformed radiomics in forecasting LC in patients with SBC after CIRT. This observation can be interpreted by the high degree of standardization of dosing regimens in patients with SBC, supporting the idea that optimizing treatment schemes can facilitate improved predictive power. Regarding the effect of bin width, Rossi et al. (157) developed a prediction model with a bin width of 1 Gy to assess gastrointestinal and genitourinary toxicity after RT in prostate cancer cases. However, Lee et al. (161) employed a smaller bin width (value of 25 cGy) to detect weight loss after RT for patients with lung cancer to reveal subtle variations between the 3D dose distribution maps; their findings indicated that distinct bin widths result in changes in dose signatures and such discrepancies influence model outputs. This discovery is consistent with the effect of diverse acquisition parameters in radiology images. Second, quality assurance equipment standards from across vendors can affect the research outcomes. Additionally, the association between radiomic and dosiomic features and failed quality assurance plans is difficult to determine, hindering the exclusion of adverse factors. Moreover, 3D dose distribution can vary depending on the material such as CTs and/or phantoms. Therefore, the impact of different treatment plans on the predictive performance of therapy and prognosis needs future investigation.

#### 6.4.4 Interpretability of the Study

The black box problem of models is another limitation of clinical application. Interpretable results are critical for clinicians. Without understanding how and why the algorithm is classifying or evaluating, the conclusions drawn are often less than acceptable. Therefore, interpretable radiomic features are an urgent requirement. For manually defined characteristics, correlating them with the biological properties of the tissue may be a promising option. They are not uniformly defined; however, their association with pathophysiology may accelerate with the development of multimodality and multi-omics studies. Therefore, establishing significant relationships between the known biological properties of different images and handcrafted signatures is promising. For deep network architecture, understanding and interpreting the depth traits learned by the algorithm is more complex. Many studies demonstrated that deep neural networks could easily be spoofed to misclassify specific tasks (89, 180, 181). Such erroneous predictions or evaluation outcomes may eventually cause fatal accidents in the medical field, making their interpretability even more urgent. Fortunately, several researchers are working on techniques to attenuate deep learning black-box perception (**Supplementary Section 1.5**) and have achieved better outcomes. However, the interpretability of depth attributes is still low and difficult to conceptualize. Therefore, translating radiomics methods into clinical practice is a challenge even now. However, these methods may evolve into new algorithms or emerging techniques developed to understand and analyze medical data in the future.

#### 6.4.5 Prospective Studies and Clinical Trials

Although the results of retrospective analyses can help with screening, diagnosis, treatment, and prognostic assessment, prediction does not change the outcome later. However, prospective studies can overcome this drawback and, after validating the algorithm with clinical data, can be targeted to guide the next treatment step. Data reporting should comply with the recommendations of the Transparent Reporting of a multivariable prediction model for Individual Prognosis or Diagnosis in the study design to ensure result validity in clinical practice (182). Zwanenburg and Lock (183) discussed that different TRIPOD analysis types have inherently varying reliabilities, and it is needed to avoid the over-fitting phenomena and build models with external validation. Peeken et al. (144) performed a *post-hoc* secondary analysis to determine the final effect in a mixed cohort of two independent institutions based on Transparent Reporting of a multivariable prediction model for Individual Prognosis or Diagnosis Type III validation requirements. Additionally, clinical trials are placing higher demands on study compliance owing to regulatory restrictions and data protection rules. Recently, national and international network initiatives (184), ethical regulation of algorithms (185), and data privacy protections (169) were further discussed to support precision medicine and AI-based paramedic programs. **Supplementary Section 1.6** discussed the design of research compliance from a legal and regulatory perspective. These initiatives have facilitated the creation of collaborative structured annotation databases to extensively assess model generalization capabilities.

## 7 Conclusions

The above-discussed analysis framework and challenges raise questions about the future development of radiomics. The first is how to look at the factors that affect model robustness. Training on a large high-quality dataset seems to be a standard measure to improve the predictive power of the algorithm. However, as the number of feature sets continues to increase, the field is gradually moving toward quantitative, high-throughput agnostic analysis, further leading to a disconnect between findings and biological significance, inherently limiting the ability to translate research results into clinical practice. Apart from the complement of experimental data, one aspect that tends to be overlooked is that task-specific expert knowledge may be more beneficial than the algorithms themselves, which is not unlikely to be a viable approach. Additionally, integrating multiple types of medical data, such as clinical data, test reports, genetic data, radiology images, pathology images, and exploring potential connections, between quantitative imaging biomarkers and biological and clinical outcomes can not only improve the performance of algorithms but also more importantly, reintroduce biological significance into the radiomic analysis. Second, considering the reproducibility and interpretability of studies and the impact of different treatment protocols at various institutions, the following studies should focus on independent validation of the robustness of existing and/or new models. The specific implementation strategies are discussed accordingly in the presented analysis framework. Third, whether radiomics can achieve clinical application is crucial for future investigation. Therefore, multicenter prospective studies and clinical trials are necessary. Finally, an open platform for radiomics analysis should be determined in the future. A rounded and systematic dissection of clinical data based on compliance with legal and ethical requirements and respecting patient privacy will better facilitate the forward development of the field.

In summary, radiomics and deep learning remain two rapidly evolving novel technologies with considerable potential value in disease diagnosis, treatment, and prognosis. A representative example is the emerging dosiomics in RT. As they continue to be studied and validated more widely, their applications in radiology will become part of clinical decision-making and give rise to more comprehensive and personalized treatments. To advance the translation of research results to clinical implementation, additional prospective studies are necessary to ensure outcome validity and generalizability and demonstrate the value of this technology for workflow and treatment decisions through expert reports in clinical trials.

## Author Contributions

YZ and XZ: conception and design the study. GZ and XQ: medical support and manuscript correction. XZ: manuscript writing. YZ, WT, XY, and LL: expert guidance and manuscript review. All authors: final approval of the manuscript. All authors contributed to the article and approved the submitted version.

## Funding

This work was supported by the National Natural Science Foundation of China (61971118) and the Science and Technology Program of Guangzhou (Grant No. 202102010472).

## Conflict of Interest

The authors declare that the research was conducted in the absence of any commercial or financial relationships that could be construed as a potential conflict of interest.

## Publisher’s Note

All claims expressed in this article are solely those of the authors and do not necessarily represent those of their affiliated organizations, or those of the publisher, the editors and the reviewers. Any product that may be evaluated in this article, or claim that may be made by its manufacturer, is not guaranteed or endorsed by the publisher.

## Abbreviations

AUC, Area under the curve; ceCT, Contrast-enhanced computed tomography; CNN, Convolutional neural networks; CT, Computed tomography; DVI, Dose-volume indices; EGFR, Epidermal growth factor receptor; GC, Gastric cancer; IBSI, Image Biomarker Standardization Initiative; ICC, Intraclass correlation coefficients; IOV, Interobserver variation; LC, Local control; LGG, Lower-grade glioma; LR, Logistic regression; MR, Magnetic resonance; NSCLC Non-small cell lung cancer; PET, Positron emission tomography; RF, Random forest; ROI, Region of interest; RP, Radiation pneumonitis; SBC, Skull-based chordomas; SNR, Signal-to-noise ratio; SVM, Support vector machine; VMAT, Volume-modifying arc therapy; WSI, Whole slide imaging.

## References

[1] Tomaszewski MR Gillies RJ. The Biological Meaning of Radiomic Features. Radiology (2021) 298:505–16. doi: 10.1148/radiol.2021202553 PMC7924519 33399513

[2] Mayerhoefer ME Materka A Langs G Haggstrom I Szczypiriski P Gibbs P. Introduction to Radiomics. J Nucl Med (2020) 61:488–95. doi: 10.2967/jnumed.118.222893 PMC9374044 32060219

[3] Wang Y Herrington DM. Machine Intelligence Enabled Radiomics. Nat Mach Intell (2021) 3:838–9. doi: 10.1038/s42256-021-00404-0

[4] Ibrahim A Primakov S Beuque M Woodruff HC Halilaj I Wu G. Radiomics for Precision Medicine: Current Challenges, Future Prospects, and the Proposal of a New Framework. Methods (2021) 188:20–9. doi: 10.1016/j.ymeth.2020.05.022 32504782

[5] Singh G Manjila S Sakla N True A Wardeh AH Beig N. Radiomics and Radiogenomics in Gliomas: A Contemporary Update. Br J Cancer (2021) 125:641–57. doi: 10.1038/s41416-021-01387-w PMC8405677 33958734

[6] Yang WC Hsu FM Yang PC. Precision Radiotherapy for Non-Small Cell Lung Cancer. J Biomed Sci (2020) 27:1–21. doi: 10.1186/s12929-020-00676-5 32693792 PMC7374898

[7] Yamashita R Long J Longacre T Peng L Berry G Martin B. Deep Learning Model for the Prediction of Microsatellite Instability in Colorectal Cancer: A Diagnostic Study. Lancet Oncol (2021) 22:132–41. doi: 10.1016/S1470-2045(20)30535-0 33387492

[8] Batenchuk C Chang HW Cimermancic P Yi ES Sadhwani A Velez V. A Machine Learning-Based Approach for the Inference of Immunotherapy Biomarker Status in Lung Adenocarcinoma From Hematoxylin and Eosin (H&E) Histopathology Images. J Clin Oncol (2020) 38:3122–3122. doi: 10.1200/JCO.2020.38.15_suppl.3122

[9] Hyun SH Ahn MS Koh YW Lee SJ. A Machine-Learning Approach Using Pet-Based Radiomics to Predict the Histological Subtypes of Lung Cancer. Clin Nucl Med (2019) 44:956–60. doi: 10.1097/Rlu.0000000000002810 31689276

[10] van Griethuysen JJM Fedorov A Parmar C Hosny A Aucoin N Narayan V. Computational Radiomics System to Decode the Radiographic Phenotype. Cancer Res (2017) 77:E104–7. doi: 10.1158/0008-5472.Can-17-0339 PMC5672828 29092951

[11] Bibault JE Xing L Giraud P El Ayachy R Giraud N Decazes P. Radiomics: A Primer for the Radiation Oncologist. Cancer Radiother (2020) 24:403–10. doi: 10.1016/j.canrad.2020.01.011 32265157

[12] Bodalal Z Trebeschi S Nguyen-Kim TDL Schats W Beets-Tan R. Radiogenomics: Bridging Imaging and Genomics. Abdominal Radiol (2019) 44:1960–84. doi: 10.1007/s00261-019-02028-w 31049614

[13] Coates JTT Pirovano G El Naqa I. Radiomic and Radiogenomic Modeling for Radiotherapy: Strategies, Pitfalls, and Challenges. J Med Imaging (2021) 8:031902. doi: 10.1117/1.Jmi.8.3.031902 PMC7985651 33768134

[14] Santucci D Faiella E Cordelli E Sicilia R de Felice C Zobel BB. 3t Mri-Radiomic Approach to Predict for Lymph Node Status in Breast Cancer Patients. Cancers (2021) 13:2228. doi: 10.3390/cancers13092228 34066451 PMC8124168

[15] Wu GY Woodruff HC Shen J Refaee T Sanduleanu S Ibrahim A. Diagnosis of Invasive Lung Adenocarcinoma Based on Chest Ct Radiomic Features of Part-Solid Pulmonary Nodules: A Multicenter Study. Radiology (2020) 297:451–8. doi: 10.1148/radiol.2020192431 32840472

[16] Brunese L Mercaldo F Reginelli A Santone A. An Ensemble Learning Approach for Brain Cancer Detection Exploiting Radiomic Features. Comput Methods Programs Biomed (2020) 185:105134. doi: 10.1016/j.cmpb.2019.105134 31675644

[17] Alvarez-Jimenez C Antunes JT Talasila N Bera K Brady JT Gollamudi J. Radiomic Texture and Shape Descriptors of the Rectal Environment on Post-Chemoradiation T2-Weighted Mri are Associated With Pathologic Tumor Stage Regression in Rectal Cancers: A Retrospective, Multi-Institution Study. Cancers (2020) 12:2027. doi: 10.3390/cancers12082027 PMC7463898 32722082

[18] Bowen SR Yuh WTC Hippe DS Wu W Partridge SC Elias S. Tumor Radiomic Heterogeneity: Multiparametric Functional Imaging to Characterize Variability and Predict Response Following Cervical Cancer Radiation Therapy. J Magn Reson Imaging (2018) 47:1388–96. doi: 10.1002/jmri.25874 PMC5899626 29044908

[19] Virginia BM Laura F Silvia R Roberto F Francesco F Eva H. Prognostic Value of Histogram Analysis in Advanced non-Small Cell Lung Cancer: A Radiomic Study. Oncotarget (2018) 9:1906–14. doi: 10.18632/oncotarget.22316 PMC5788608 29416740

[20] Zhou JL Lu JH Gao C Zeng JJ Zhou CY Lai XB. Predicting the Response to Neoadjuvant Chemotherapy for Breast Cancer: Wavelet Transforming Radiomics in Mri. BMC Cancer (2020) 20:1–10. doi: 10.1186/s12885-020-6523-2 PMC7003343 32024483

[21] Trivizakis E Ioannidis GS Souglakos I Karantanas AH Tzardi M Marias K. A Neural Pathomics Framework for Classifying Colorectal Cancer Histopathology Images Based on Wavelet Multi-Scale Texture Analysis. Sci Rep (2021) 11:1–10. doi: 10.1038/s41598-021-94781-6 34330946 PMC8324876

[22] Leithner D Bernard-Davila B Martinez DF Horvat JV Jochelson MS Marino MA. Radiomic Signatures Derived From Diffusion-Weighted Imaging for the Assessment of Breast Cancer Receptor Status and Molecular Subtypes. Mol Imaging Biol (2020) 22:453–61. doi: 10.1007/s11307-019-01383-w PMC7062654 31209778

[23] Park YW Kim S Ahn SS Han K Kang SG Chang JH. Magnetic Resonance Imaging-Based 3-Dimensional Fractal Dimension and Lacunarity Analyses may Predict the Meningioma Grade. Eur Radiol (2020) 30:4615–22. doi: 10.1007/s00330-020-06788-8 32274524

[24] Mashayekhi R Parekh VS Faghih M Singh VK Jacobs MA Zaheer A. Radiomic Features of the Pancreas on Ct Imaging Accurately Differentiate Functional Abdominal Pain, Recurrent Acute Pancreatitis, and Chronic Pancreatitis. Eur J Radiol (2020) 123:108778. doi: 10.1016/j.ejrad.2019.108778 31846864 PMC7968044

[25] Pavic M Bogowicz M Wurms X Glatz S Finazzi T Riesterer O. Influence of Inter-Observer Delineation Variability on Radiomics Stability in Different Tumor Sites. Acta Oncol (2018) 57:1070–4. doi: 10.1080/0284186x.2018.1445283 29513054

[26] Parmar C Velazquez ER Leijenaar R Jermoumi M Carvalho S Mak RH. Robust Radiomics Feature Quantification Using Semiautomatic Volumetric Segmentation. PloS One (2014) 9:e102107. doi: 10.1371/journal.pone.0102107 25025374 PMC4098900

[27] Iantsen A Ferreira M Lucia F Jaouen V Reinhold C Bonaffini P. Convolutional Neural Networks for Pet Functional Volume Fully Automatic Segmentation: Development and Validation in a Multi-Center Setting. Eur J Nucl Med Mol Imaging (2021) 48:3444–56. doi: 10.1007/s00259-021-05244-z PMC8440243 33772335

[28] Pfaehler E Burggraaff C Kramer G Zijlstra J Hoekstra OS Jalving M. Pet Segmentation of Bulky Tumors: Strategies and Workflows to Improve Inter-Observer Variability. PloS One (2020) 15:e0230901. doi: 10.1371/journal.pone.0230901 32226030 PMC7105134

[29] Zhuang MZ Garcia DV Kramer GM Frings V Smit EF Dierckx R. Variability and Repeatability of Quantitative Uptake Metrics in F-18-Fdg Pet/Ct of Non-Small Cell Lung Cancer: Impact of Segmentation Method, Uptake Interval, and Reconstruction Protocol. J Nucl Med (2019) 60:600–7. doi: 10.2967/jnumed.118.216028 30389824

[30] Martens RM Koopman T Noij DP Pfaehler E Ubelhor C Sharma S. Predictive Value of Quantitative F-18-Fdg-Pet Radiomics Analysis in Patients With Head and Neck Squamous Cell Carcinoma. Ejnmmi Res (2020) 10:1–15. doi: 10.1186/s13550-020-00686-2 32894373 PMC7477048

[31] Anter AM Hassenian AE. Ct Liver Tumor Segmentation Hybrid Approach Using Neutrosophic Sets, Fast Fuzzy C-Means and Adaptive Watershed Algorithm. Artif Intell Med (2019) 97:105–17. doi: 10.1016/j.artmed.2018.11.007 30558825

[32] Yin XX Jian YX Zhang Y Zhang YC Wu JL Lu H. Automatic Breast Tissue Segmentation in Mris With Morphology Snake and Deep Denoiser Training *via* Extended Stein’s Unbiased Risk Estimator. Health Inf Sci Syst (2021) 9:1–21. doi: 10.1007/s13755-021-00143-x 33898019 PMC8021687

[33] Huang QH Luo YZ Zhang QZ. Breast Ultrasound Image Segmentation: A Survey. Int J Comput Assisted Radiol Surg (2017) 12:493–507. doi: 10.1007/s11548-016-1513-1 28070777

[34] Durnea CM Siddiqi S Nazarian D Munneke G Sedgwick PM Doumouchtsis SK. 3d-Volume Rendering of the Pelvis With Emphasis on Paraurethral Structures Based on Mri Scans and Comparisons Between 3d Slicer and Osirix (R). J Med Syst (2021) 45:27. doi: 10.1007/s10916-020-01695-3 33469726 PMC7815623

[35] Tasnadi EA Toth T Kovacs M Diosdi A Pampaloni F Molnar J. 3d-Cell-Annotator: An Open-Source Active Surface Tool for Single-Cell Segmentation in 3d Microscopy Images. Bioinformatics (2020) 36:2948–9. doi: 10.1093/bioinformatics/btaa029 PMC7203751 31950986

[36] Zhou YQ Son GH Shi YQ Yu YJ Li MY Zhang Q. Quantitative Segmentation Analysis of the Radiological Changes by Using Itk-Snap: Risk Assessment of the Severity and Recurrence of Medication-Related Osteonecrosis of the Jaw. Int J Med Sci (2021) 18:2209–16. doi: 10.7150/ijms.56408 PMC8040413 33859529

[37] Kronig ODM Kronig SAJ Vrooman HA Veenland JF Jippes M Boen T. Introducing a New Method for Classifying Skull Shape Abnormalities Related to Craniosynostosis. Eur J Pediatr (2020) 179:1569–77. doi: 10.1007/s00431-020-03643-2 PMC7479008 32303825

[38] Nioche C Orlhac F Boughdad S Reuze S Goya-Outi J Robert C. Lifex: A Freeware for Radiomic Feature Calculation in Multimodality Imaging to Accelerate Advances in the Characterization of Tumor Heterogeneity. Cancer Res (2018) 78:4786–9. doi: 10.1158/0008-5472.Can-18-0125 29959149

[39] Rueden CT Schindelin J Hiner MC DeZonia BE Walter AE Arena ET. Imagej2: Imagej for the Next Generation of Scientific Image Data. BMC Bioinf (2017) 18:1–26. doi: 10.1186/s12859-017-1934-z PMC5708080 29187165

[40] Sun LY Chen P Xiang W Chen P Ga WY Zhang KJ. Smartpaint: A Co-Creative Drawing System Based on Generative Adversarial Networks. Front Inf Technol Electron Eng (2019) 20:1644–56. doi: 10.1631/Fitee.1900386

[41] Bettinelli A Branchini M De Monte F Scaggion A Paiusco M. Technical Note: An Ibex Adaption Toward Image Biomarker Standardization. Med Phys (2020) 47:1167–73. doi: 10.1002/mp.13956 31830303

[42] Awe AM Rendell VR Lubner MG Winslow ER. Texture Analysis an Emerging Clinical Tool for Pancreatic Lesions. Pancreas (2020) 49:301–12. doi: 10.1097/Mpa.0000000000001495 PMC7135958 32168248

[43] Xu ML Tang Q Li MX Liu YL Li F. An Analysis of Ki -67 Expression in Stage 1 Invasive Ductal Breast Carcinoma Using Apparent Diffusion Coefficient Histograms. Quantit Imaging Med Surg (2021) 11:1518–31. doi: 10.21037/qims-20-615 PMC7930667 33816188

[44] Fornacon-Wood I Mistry H Ackermann CJ Blackhall F McPartlin A Faivre-Finn C. Reliability and Prognostic Value of Radiomic Features are Highly Dependent on Choice of Feature Extraction Platform. Eur Radiol (2020) 30:6241–50. doi: 10.1007/s00330-020-06957-9 PMC7553896 32483644

[45] Keek S Sanduleanu S Wesseling F de Roest R van den Brekel M van der Heijden M. Computed Tomography-Derived Radiomic Signature of Head and Neck Squamous Cell Carcinoma (Peri)Tumoral Tissue for the Prediction of Locoregional Recurrence and Distant Metastasis After Concurrent Chemo-Radiotherapy. PloS One (2020) 15:e0232639. doi: 10.1371/journal.pone.0237048 32442178 PMC7244120

[46] Sandrasegaran K Lin YN Asare-Sawiri M Taiyini T Tann M. Ct Texture Analysis of Pancreatic Cancer. Eur Radiol (2019) 29:1067–73. doi: 10.1007/s00330-018-5662-1 30116961

[47] Echegaray S Bakr S Rubin DL Napel S. Quantitative Image Feature Engine (Qife): An Open-Source, Modular Engine for 3d Quantitative Feature Extraction From Volumetric Medical Images. J Digital Imaging (2018) 31:403–14. doi: 10.1007/s10278-017-0019-x PMC6113159 28993897

[48] Rossi G Barabino E Fedeli A Ficarra G Coco S Russo A. Y Radiomic Detection of Egfr Mutations in Nsclc. Cancer Res (2021) 81:724–31. doi: 10.1158/0008-5472.Can-20-0999 33148663

[49] Agazzi GM Ravanelli M Roca E Medicina D Balzarini P Pessina C. Ct Texture Analysis for Prediction of Egfr Mutational Status and Alk Rearrangement in Patients With Non-Small Cell Lung Cancer. Radiologia Med (2021) 126:786–94. doi: 10.1007/s11547-020-01323-7 33512651

[50] Xu X Zhang HL Liu QP Sun SW Zhang J Zhu FP. Radiomic Analysis of Contrast-Enhanced Ct Predicts Microvascular Invasion and Outcome in Hepatocellular Carcinoma. J Hepatol (2019) 70:1133–44. doi: 10.1016/j.jhep.2019.02.023 30876945

[51] Haubold J Demircioglu A Gratz M Glas M Wrede K Sure U. Non-Invasive Tumor Decoding and Phenotyping of Cerebral Gliomas Utilizing Multiparametric F-18-Fet Pet-Mri and Mr Fingerprinting. Eur J Nucl Med Mol Imaging (2020) 47:1435–45. doi: 10.1007/s00259-019-04602-2 31811342

[52] Peng YT Lin P Wu LY Wan D Zhao YJ Liang L. Ultrasound-Based Radiomics Analysis for Preoperatively Predicting Different Histopathological Subtypes of Primary Liver Cancer. Front Oncol (2020) 10:1646. doi: 10.3389/fonc.2020.01646 33072550 PMC7543652

[53] Beer JC Tustison NJ Cook PA Davatzikos C Sheline YI Shinohara RT. Longitudinal Combat: A Method for Harmonizing Longitudinal Multi-Scanner Imaging Data. Neuroimage (2020) 220:117129. doi: 10.1016/j.neuroimage.2020.117129 32640273 PMC7605103

[54] Mahon RN Ghita M Hugo GD Weiss E. Combat Harmonization for Radiomic Features in Independent Phantom and Lung Cancer Patient Computed Tomography Datasets. Phys Med Biol (2020) 65:015010. doi: 10.1088/1361-6560/ab6177 31835261

[55] Badic B Da-Ano R Poirot K Jaouen V Magnin B Gagniere J. Prediction of Recurrence After Surgery in Colorectal Cancer Patients Using Radiomics From Diagnostic Contrast-Enhanced Computed Tomography: A Two-Center Study. Eur Radiol (2022) 32(1):405–14. doi: 10.1007/s00330-021-08104-4 34170367

[56] Nakajo M Jinguji M Tani A Kikuno H Hirahara D Togami S. Application of a Machine Learning Approach for the Analysis of Clinical and Radiomic Features of Pretreatment [F-18]-Fdg Pet/Ct to Predict Prognosis of Patients With Endometrial Cancer. Mol Imaging Biol (2021) 23:756–65. doi: 10.1007/s11307-021-01599-9 33763816

[57] Da-ano R Masson I Lucia F Dore M Robin P Alfieri J. Performance Comparison of Modified Combat for Harmonization of Radiomic Features for Multicenter Studies. Sci Rep (2020) 10:1–12. doi: 10.1038/s41598-020-66110-w 32581221 PMC7314795

[58] Li Y Tan GH Vangel M Hall J Cai WL. Influence of Feature Calculating Parameters on the Reproducibility of Ct Radiomic Features: A Thoracic Phantom Study. Quantit Imaging Med Surg (2020) 10:1775–+. doi: 10.21037/qims-19-921 PMC7417756 32879856

[59] Krarup MMK Nygard L Vogelius IR Andersen FL Cook G Goh V. Heterogeneity in Tumours: Validating the Use of Radiomic Features on F-18-Fdg Pet/Ct Scans of Lung Cancer Patients as a Prognostic Tool. Radiother Oncol (2020) 144:72–8. doi: 10.1016/j.radonc.2019.10.012 31733491

[60] Le NQK Hung TNK Do DT Lam LHT Dang LH Huynh TT. Radiomics-Based Machine Learning Model for Efficiently Classifying Transcriptome Subtypes in Glioblastoma Patients From Mri. Comput Biol Med (2021) 132:104320. doi: 10.1016/j.compbiomed.2021.104320 33735760

[61] Zhang JY Zhao XM Zhao Y Zhang JM Zhang ZQ Wang JF. Value of Pre-Therapy F-18-Fdg Pet/Ct Radiomics in Predicting Egfr Mutation Status in Patients With Non-Small Cell Lung Cancer. Eur J Nucl Med Mol Imaging (2020) 47:1137–46. doi: 10.1007/s00259-019-04592-1 31728587

[62] Meng LW Dong D Chen X Fang MJ Wang RP Li J. 2d and 3d Ct Radiomic Features Performance Comparison in Characterization of Gastric Cancer: A Multi-Center Study. IEEE J Biomed Health Inf (2021) 25:755–63. doi: 10.1109/Jbhi.2020.3002805 32750940

[63] Li ML Zhang J Dan YB Yao YF Dai WX Cai GX. A Clinical-Radiomics Nomogram for the Preoperative Prediction of Lymph Node Metastasis in Colorectal Cancer. J Trans Med (2020) 18:1–10. doi: 10.1186/s12967-020-02215-0 PMC6993349 32000813

[64] Song L Zhu ZC Mao L Li XL Han W Du HY. Clinical, Conventional Ct and Radiomic Feature-Based Machine Learning Models for Predicting Alk Rearrangement Status in Lung Adenocarcinoma Patients. Front Oncol (2020) 10:369. doi: 10.3389/fonc.2020.00369 32266148 PMC7099003

[65] Mahon RN Hugo GD Weiss E. Repeatability of Texture Features Derived From Magnetic Resonance and Computed Tomography Imaging and Use in Predictive Models for Non-Small Cell Lung Cancer Outcome. Phys Med Biol 64 (2019) 64:145007. doi: 10.1088/1361-6560/ab18d3 30978707

[66] Shiri I Maleki H Hajianfar G Abdollahi H Ashrafinia S Hatt M. Next-Generation Radiogenomics Sequencing for Prediction of Egfr and Kras Mutation Status in Nsclc Patients Using Multimodal Imaging and Machine Learning Algorithms. Mol Imaging Biol (2020) 22:1132–48. doi: 10.1007/s11307-020-01487-8 32185618

[67] Zhao SJ Hou DH Zheng XM Song W Liu XQ Wang SC. Mri Radiomic Signature Predicts Intracranial Progression-Free Survival in Patients With Brain Metastases of Alk-Positive non-Small Cell Lung Cancer. Trans Lung Cancer Res (2021) 10:368. doi: 10.21037/tlcr-20-361 PMC7867779 33569319

[68] Liang L Zhi X Sun Y Li HR Wang JJ Xu JX. A Nomogram Based on a Multiparametric Ultrasound Radiomics Model for Discrimination Between Malignant and Benign Prostate Lesions. Front Oncol (2021) 11:610785. doi: 10.3389/fonc.2021.610785 33738255 PMC7962672

[69] Fang MJ He BX Li L Dong D Yang X Li C. Ct Radiomics can Help Screen the Coronavirus Disease 2019 (Covid-19): A Preliminary Study. Sci China-Information Sci (2020) 63:1–8. doi: 10.1007/s11432-020-2849-3

[70] Berenguer R Pastor-Juan MD Canales-Vazquez J Castro-Garcia M Villas MV Legorburo FM. Radiomics of Ct Features may be Nonreproducible and Redundant: Influence of Ct Acquisition Parameters. Radiology (2018) 288:407–15. doi: 10.1148/radiol.2018172361 29688159

[71] Kim CH Bhattacharjee S Prakash D Kang S Cho NH Kim HC. Artificial Intelligence Techniques for Prostate Cancer Detection Through Dual-Channel Tissue Feature Engineering. Cancers (2021) 13:1524. doi: 10.3390/cancers13071524 33810251 PMC8036750

[72] Quiaoit K DiCenzo D Fatima K Bhardwaj D Sannachi L Gangeh M. Quantitative Ultrasound Radiomics for Therapy Response Monitoring in Patients With Locally Advanced Breast Cancer: Multi-Institutional Study Results. PloS One (2020) 15:e0236182. doi: 10.1371/journal.pone.0236182 32716959 PMC7384762

[73] Zhou XC Lin QM Gui YY Wang ZX Liu MH Lu H. Multimodal Mr Images-Based Diagnosis of Early Adolescent Attention-Deficit/Hyperactivity Disorder Using Multiple Kernel Learning. Front Neurosci (2021) 15:710133. doi: 10.3389/fnins.2021.710133 34594183 PMC8477011

[74] Fallerini C Daga S Mantovani S Benetti E Picchiotti N Francisci D. Association of Toll-Like Receptor 7 Variants With Life-Threatening Covid-19 Disease in Males: Findings From a Nested Case-Control Study. Elife (2021) 10:e67569. doi: 10.7554/eLife.67569 33650967 PMC7987337

[75] Gevaert O Echegaray S Khuong A Hoang CD Shrager JB Jensen KC. Predictive Radiogenomics Modeling of Egfr Mutation Status in Lung Cancer. Sci Rep (2017) 7:1–8. doi: 10.1038/srep41674 28139704 PMC5282551

[76] Zhang B He X Ouyang FS Gu DS Dong YH Zhang L. Radiomic Machine-Learning Classifiers for Prognostic Biomarkers of Advanced Nasopharyngeal Carcinoma. Cancer Lett (2017) 403:21–7. doi: 10.1016/j.canlet.2017.06.004 28610955

[77] Huang C Cintra M Brennan K Zhou M Colevas AD Fischbein N. Development and Validation of Radiomic Signatures of Head and Neck Squamous Cell Carcinoma Molecular Features and Subtypes. Ebiomedicine (2019) 45:70–80. doi: 10.1016/j.ebiom.2019.06.034 31255659 PMC6642281

[78] Limkin EJ Reuze S Carre A Sun R Schernberg A Alexis A. The Complexity of Tumor Shape, Spiculatedness, Correlates With Tumor Radiomic Shape Features. Sci Rep (2019) 9:1–12. doi: 10.1038/s41598-019-40437-5 30867443 PMC6416263

[79] Lemaitre G Nogueira F Aridas CK. Imbalanced-Learn: A Python Toolbox to Tackle the Curse of Imbalanced Datasets in Machine Learning. J Mach Learn Res (2017) 18:559–63.

[80] Salmanpour MR Shamsaei M Saberi A Hajianfar G Soltanian-Zadeh H Rahmim A. Robust Identification of Parkinson’s Disease Subtypes Using Radiomics and Hybrid Machine Learning. Comput Biol Med (2021) 129:104142. doi: 10.1016/j.compbiomed.2020.104142 33260101

[81] Rundo L Beer L Ursprung S Martin-Gonzalez P Markowetz F Brenton JD. Tissue-Specific and Interpretable Sub-Segmentation of Whole Tumour Burden on Ct Images by Unsupervised Fuzzy Clustering. Comput Biol Med (2020) 120:103751. doi: 10.1016/j.compbiomed.2020.103751 32421652 PMC7248575

[82] Huang CY Lee CC Yang HC Lin CJ Wu HM Chung WY. Radiomics as Prognostic Factor in Brain Metastases Treated With Gamma Knife Radiosurgery. J Neuro-Oncol (2020) 146:439–49. doi: 10.1007/s11060-019-03343-4 32020474

[83] Khorrami M Prasanna P Gupta A Patil P Velu PD Thawani R. Changes in Ct Radiomic Features Associated With Lymphocyte Distribution Predict Overall Survival and Response to Immunotherapy in non-Small Cell Lung Cancer. Cancer Immunol Res (2020) 8:108–19. doi: 10.1158/2326-6066.Cir-19-0476 PMC7718609 31719058

[84] Wulczyn E Steiner DF Xu ZY Sadhwani A Wang HW Flament-Auvigne I. Deep Learning-Based Survival Prediction for Multiple Cancer Types Using Histopathology Images. PloS One (2020) 15:e0233678. doi: 10.1371/journal.pone.0233678 32555646 PMC7299324

[85] Jiang M Li CL Luo XM Chuan ZR Lv WZ Li X. Ultrasound-Based Deep Learning Radiomics in the Assessment of Pathological Complete Response to Neoadjuvant Chemotherapy in Locally Advanced Breast Cancer. Eur J Cancer (2021) 147:95–105. doi: 10.1016/j.ejca.2021.01.028 33639324

[86] Li C Dong D Li L Gong W Li XH Bai Y. Classification of Severe and Critical Covid-19 Using Deep Learning and Radiomics. IEEE J Biomed Health Inf (2020) 24:3585–94. doi: 10.1109/Jbhi.2020.3036722 PMC8545168 33166256

[87] Dai HJ Lu MH Huang BS Tang MM Pang TT Liao B. Considerable Effects of Imaging Sequences, Feature Extraction, Feature Selection, and Classifiers on Radiomics-Based Prediction of Microvascular Invasion in Hepatocellular Carcinoma Using Magnetic Resonance Imaging. Quantit Imaging Med Surg (2021) 11:1836–53. doi: 10.21037/qims-20-218 PMC8047362 33936969

[88] Li Z Zhang JH Tan T Teng XC Sun XL Zhao H. Deep Learning Methods for Lung Cancer Segmentation in Whole-Slide Histopathology Images-the Acdc@Lunghp Challenge 2019. IEEE J Biomed Health Inf (2021) 25:429–40. doi: 10.1109/Jbhi.2020.3039741 33216724

[89] Liang B Li HC Su MQ Li XR Shi WC Wang XF. Detecting Adversarial Image Examples in Deep Neural Networks With Adaptive Noise Reduction. IEEE Trans Dependable Secure Computing (2021) 18:72–85. doi: 10.1109/Tdsc.2018.2874243

[90] Woerl AC Eckstein M Geiger J Wagner DC Daher T Stenzel P. Deep Learning Predicts Molecular Subtype of Muscle-Invasive Bladder Cancer From Conventional Histopathological Slides. Eur Urol (2020) 78:256–64. doi: 10.1016/j.eururo.2020.04.023 32354610

[91] Tan W Zhou L Li X Yang X Chen Y Yang J. Automated Vessel Segmentation in Lung Ct and Cta Images *via* Deep Neural Networks. J Xray Sci Technol (2021) 29(6):1123–37. doi: 10.3233/XST-210955 34421004

[92] Xu Y Jia ZP Wang LB Ai YQ Zhang F Lai MD. Large Scale Tissue Histopathology Image Classification, Segmentation, and Visualization *via* Deep Convolutional Activation Features. BMC Bioinf (2017) 18:1–17. doi: 10.1186/s12859-017-1685-x PMC5446756 28549410

[93] Amyar A Modzelewski R Li H Ruan S. Multi-Task Deep Learning Based Ct Imaging Analysis for Covid-19 Pneumonia: Classification and Segmentation. Comput Biol Med (2020) 126:104037. doi: 10.1016/j.compbiomed.2020.104037 33065387 PMC7543793

[94] Guo Z Guo N Gong K Zhong SA Li QZ. Gross Tumor Volume Segmentation for Head and Neck Cancer Radiotherapy Using Deep Dense Multi-Modality Network. Phys Med Biol (2019) 64:205015. doi: 10.1088/1361-6560/ab440d 31514173 PMC7186044

[95] Avanzo M Stancanello J Pirrone G Sartor G. Radiomics and Deep Learning in Lung Cancer. Strahlenther Und Onkologie (2020) 196:879–87. doi: 10.1007/s00066-020-01625-9 32367456

[96] Parekh VS Jacobs MA. Deep Learning and Radiomics in Precision Medicine. Expert Rev Precis Med Drug Dev (2019) 4:59–72. doi: 10.1080/23808993.2019.1585805 31080889 PMC6508888

[97] Han ZY Wei BZ Xi XM Chen B Yin YL Li S. Unifying Neural Learning and Symbolic Reasoning for Spinal Medical Report Generation. Med Image Anal (2021) 67:101872. doi: 10.1016/j.media.2020.101872 33142134

[98] Tian J. Artificial Intelligence Advanced Imaging Report Standardization and Intra-Interdisciplinary Clinical Workflow. Ebiomedicine (2019) 44:4–5. doi: 10.1016/j.ebiom.2019.05.049 31133541 PMC6606521

[99] Yue ZJ Ding S Zhao WD Wang H Ma J Zhang YT. Automatic Cin Grades Prediction of Sequential Cervigram Image Using Lstm With Multistate Cnn Features. IEEE J Biomed Health Inf (2020) 24:844–54. doi: 10.1109/Jbhi.2019.2922682 31199278

[100] Whitney HM Li H Ji Y Liu PF Giger ML. Comparison of Breast Mri Tumor Classification Using Human-Engineered Radiomics, Transfer Learning From Deep Convolutional Neural Networks, and Fusion Method. Proc IEEE (2020) 108:163–77. doi: 10.1109/Jproc.2019.2950187 PMC8152568 34045769

[101] Wang YF Yue WW Li XL Liu SY Guo LH Xu HX. Comparison Study of Radiomics and Deep Learning-Based Methods for Thyroid Nodules Classification Using Ultrasound Images. IEEE Access (2020) 8:52010–7. doi: 10.1109/Access.2020.2980290

[102] Oakden-Rayner L Carneiro G Bessen T Nascimento JC Bradley AP Palmer LJ. Precision Radiology: Predicting Longevity Using Feature Engineering and Deep Learning Methods in a Radiomics Framework. Sci Rep (2017) 7:1–13. doi: 10.1038/s41598-017-01931-w 28490744 PMC5431941

[103] Pang T Wong JHD Ng WL Chan CS. Semi-Supervised Gan-Based Radiomics Model for Data Augmentation in Breast Ultrasound Mass Classification. Comput Methods Programs BioMed (2021) 203:106018. doi: 10.1016/j.cmpb.2021.106018 33714900

[104] Li C Xu JX Liu QG Zhou YJ Mou LS Pu ZH. Multi-View Mammographic Density Classification by Dilated and Attention-Guided Residual Learning. Ieee-Acm Trans Comput Biol Bioinf (2021) 18:1003–13. doi: 10.1109/Tcbb.2020.2970713 32012021

[105] Zhuang FZ Qi ZY Duan KY Xi DB Zhu YC Zhu HS. A Comprehensive Survey on Transfer Learning. Proc IEEE (2021) 109:43–76. doi: 10.1109/Jproc.2020.3004555

[106] Xue LY Jiang ZY Fu TT Wang QM Zhu YL Dai M. Transfer Learning Radiomics Based on Multimodal Ultrasound Imaging for Staging Liver Fibrosis. Eur Radiol (2020) 30:2973–83. doi: 10.1007/s00330-019-06595-w PMC7160214 31965257

[107] Basha SHS Vinakota SK Pulabaigari V Mukherjee S Dubey SR. Autotune: Automatically Tuning Convolutional Neural Networks for Improved Transfer Learning. Neural Netw (2021) 133:112–22. doi: 10.1016/j.neunet.2020.10.009 33181405

[108] Yan T Wong PK Qin YY. Deep Learning for Diagnosis of Precancerous Lesions in Upper Gastrointestinal Endoscopy: A Review. World J Gastroenterol (2021) 27:2531–44. doi: 10.3748/wjg.v27.i20.2531 PMC8160615 34092974

[109] Weiss J Taron J Jin ZX Mayrhofer T Aerts HJWL Lu MT. Radiologists can Visually Predict Mortality Risk Based on the Gestalt of Chest Radiographs Comparable to a Deep Learning Network. Sci Rep (2021) 11:1–9. doi: 10.1038/s41598-021-99107-0 34599265 PMC8486799

[110] Jiang XY Ma JY Xiao GB Shao ZF Guo XJ. A Review of Multimodal Image Matching: Methods and Applications. Inf Fusion (2021) 73:22–71. doi: 10.1016/j.inffus.2021.02.012

[111] Kolossvary M Gerstenblith G Bluemke DA Fishman EK Mandler RN Kickler TS. Contribution of Risk Factors to the Development of Coronary Atherosclerosis as Confirmed *via* Coronary Ct Angiography: A Longitudinal Radiomics-Based Study. Radiology (2021) 299:97–106. doi: 10.1148/radiol.2021203179 33591887 PMC7997618

[112] Benedetti G Mori M Panzeri MM Barbera M Palumbo D Sini C. Ct-Derived Radiomic Features to Discriminate Histologic Characteristics of Pancreatic Neuroendocrine Tumors. Radiologia Med (2021) 126:745–60. doi: 10.1007/s11547-021-01333-z 33523367

[113] Jiang XR Li JX Kan YY Yu T Chang SJ Sha XZ. Mri Based Radiomics Approach With Deep Learning for Prediction of Vessel Invasion in Early-Stage Cervical Cancer. Ieee-Acm Trans Comput Biol Bioinf (2021) 18:995–1002. doi: 10.1109/Tcbb.2019.2963867 31905143

[114] Xie XJ Liu SY Chen JY Zhao Y Jiang J Wu L. Development of Unenhanced Ct-Based Imaging Signature for Bap1 Mutation Status Prediction in Malignant Pleural Mesothelioma: Consideration of 2d and 3d Segmentation. Lung Cancer (2021) 157:30–9. doi: 10.1016/j.lungcan.2021.04.023 34052706

[115] Du Y Fang Z Jiao J Xi G Zhu C Ren Y. Application of Ultrasound-Based Radiomics Technology in Fetal-Lung-Texture Analysis in Pregnancies Complicated by Gestational Diabetes and/or Pre-Eclampsia. Ultrasound Obstet Gynecol (2021) 57:804–12. doi: 10.1002/uog.22037 32250510

[116] Salvatore C Castiglioni I Cerasa A. Radiomics Approach in the Neurodegenerative Brain. Aging Clin Exp Res (2021) 33:1709–11. doi: 10.1007/s40520-019-01299-z 31428998

[117] Ligero M Garcia-Ruiz A Viaplana C Villacampa G Raciti MV Landa J. A Ct-Based Radiomics Signature is Associated With Response to Immune Checkpoint Inhibitors in Advanced Solid Tumors. Radiology (2021) 299:109–19. doi: 10.1148/radiol.2021200928 33497314

[118] Bhandari AP Liong R Koppen J Murthy SV Lasocki A. Noninvasive Determination of Idh and 1p19q Status of Lower-Grade Gliomas Using Mri Radiomics: A Systematic Review. Am J Neuroradiology (2021) 42:94–101. doi: 10.3174/ajnr.A6875 PMC7814803 33243896

[119] Choi YS Bae S Chang JH Kang SG Kim SH Kim J. Fully Automated Hybrid Approach to Predict the Idh Mutation Status of Gliomas *via* Deep Learning and Radiomics. Neuro-Oncology (2021) 23:304–13. doi: 10.1093/neuonc/noaa177 PMC7906063 32706862

[120] Guo Y Wang Q Guo Y Zhang YY Fu Y Zhang HM. Preoperative Prediction of Perineural Invasion With Multi-Modality Radiomics in Rectal Cancer. Sci Rep (2021) 11:1–11. doi: 10.1038/s41598-021-88831-2 33941817 PMC8093213

[121] Khan MA Ashraf I Alhaisoni M Damasevicius R Scherer R Rehman A. Multimodal Brain Tumor Classification Using Deep Learning and Robust Feature Selection: A Machine Learning Application for Radiologists. Diagnostics (2020) 10:565. doi: 10.3390/diagnostics10080565 PMC7459797 32781795

[122] Wu J Li C Gensheimer M Padda S Kato F Shirato H. Radiological Tumour Classification Across Imaging Modality and Histology. Nat Mach Intell (2021) 3:787–+. doi: 10.1038/s42256-021-00377-0 PMC8612063 34841195

[123] Zhao JF Li DW Xiao XJ Accorsi F Marshall H Cossetto T. United Adversarial Learning for Liver Tumor Segmentation and Detection of Multi-Modality Non-Contrast Mri. Med Image Anal (2021) 73:102154. doi: 10.1016/j.media.2021.102154 34280670

[124] Alvarez-Jimenez C Sandino AA Prasanna P Gupta A Viswanath SE Romero E. Identifying Cross-Scale Associations Between Radiomic and Pathomic Signatures of Non-Small Cell Lung Cancer Subtypes: Preliminary Results. Cancers (2020) 12:3663. doi: 10.3390/cancers12123663 PMC7762258 33297357

[125] Giardina G Micko A Bovenkamp D Krause A Placzek F Papp L. Morpho-Molecular Metabolic Analysis and Classification of Human Pituitary Gland and Adenoma Biopsies Based on Multimodal Optical Imaging. Cancers (2021) 13:3234. doi: 10.3390/cancers13133234 34209497 PMC8267638

[126] Calisto FM Santiago C Nunes N Nascimento JC. Introduction of Human-Centric Ai Assistant to Aid Radiologists for Multimodal Breast Image Classification. Int J Human-Computer Stud (2021) 150:102607. doi: 10.1016/j.ijhcs.2021.102607

[127] Dohan A Gallix B Guiu B Le Malicot K Reinhold C Soyer P. Early Evaluation Using a Radiomic Signature of Unresectable Hepatic Metastases to Predict Outcome in Patients With Colorectal Cancer Treated With Folfiri and Bevacizumab. Gut (2020) 69:531–9. doi: 10.1136/gutjnl-2018-316407 31101691

[128] Chen MY Cao JS Hu JH Topatana W Li SJ Juengpanich S. Clinical-Radiomic Analysis for Pretreatment Prediction of Objective Response to First Transarterial Chemoembolization in Hepatocellular Carcinoma. Liver Cancer (2021) 10:38–51. doi: 10.1159/000512028 33708638 PMC7923935

[129] Dissaux G Visvikis D Da-ano R Pradier O Chajon E Barillot I. Pretreatment F-18-Fdg Pet/Ct Radiomics Predict Local Recurrence in Patients Treated With Stereotactic Body Radiotherapy for Early-Stage Non-Small Cell Lung Cancer: A Multicentric Study. J Nucl Med (2020) 61:814–20. doi: 10.2967/jnumed.119.228106 31732678

[130] Fatima K Dasgupta A DiCenzo D Kolios C Quiaoit K Saifuddin M. Ultrasound Delta-Radiomics During Radiotherapy to Predict Recurrence in Patients With Head and Neck Squamous Cell Carcinoma. Clin Trans Radiat Oncol (2021) 28:62–70. doi: 10.1016/j.ctro.2021.03.002 PMC7985224 33778174

[131] Xiong QQ Zhou XZ Liu ZY Lei CQ Yang CQ Yang M. Multiparametric Mri-Based Radiomics Analysis for Prediction of Breast Cancers Insensitive to Neoadjuvant Chemotherapy. Clin Trans Oncol (2020) 22:50–9. doi: 10.1007/s12094-019-02109-8 30977048

[132] DiCenzo D Quiaoit K Fatima K Bhardwaj D Sannachi L Gangeh M. Quantitative Ultrasound Radiomics in Predicting Response to Neoadjuvant Chemotherapy in Patients With Locally Advanced Breast Cancer: Results From Multi-Institutional Study. Cancer Med (2020) 9:5798–806. doi: 10.1002/cam4.3255 PMC7433820 32602222

[133] Hu YH Xie CY Yang H Ho JWK Wen J Han LJ. Computed Tomography-Based Deep-Learning Prediction of Neoadjuvant Chemoradiotherapy Treatment Response in Esophageal Squamous Cell Carcinoma. Radiother Oncol (2021) 154:6–13. doi: 10.1016/j.radonc.2020.09.014 32941954

[134] Haider SP Sharaf K Zeevi T Baumeister P Reichel C Forghani R. Prediction of Post-Radiotherapy Locoregional Progression in Hpv-Associated Oropharyngeal Squamous Cell Carcinoma Using Machine-Learning Analysis of Baseline Pet/Ct Radiomics. Trans Oncol (2021) 14:100906. doi: 10.1016/j.tranon.2020.100906 PMC7568193 33075658

[135] Ferreira M Lovinfosse P Hermesse J Decuypere M Rousseau C Lucia F. [F-18]Fdg Pet Radiomics to Predict Disease-Free Survival in Cervical Cancer: A Multi-Scanner/Center Study With External Validation. Eur J Nucl Med Mol Imaging (2021) 48:3432–43. doi: 10.1007/s00259-021-05303-5 PMC8440288 33772334

[136] Kickingereder P Isensee F Tursunova I Petersen J Neuberger U Bonekamp D. Automated Quantitative Tumour Response Assessment of Mri in Neuro-Oncology With Artificial Neural Networks: A Multicentre, Retrospective Study. Lancet Oncol (2019) 20:728–40. doi: 10.1016/S1470-2045(19)30098-1 30952559

[137] Crombe A Perier C Kind M De Senneville BD Le Loarer F Italiano A. T2 -Based Mri Delta-Radiomics Improve Response Prediction in Soft-Tissue Sarcomas Treated by Neoadjuvant Chemotherapy. J Magn Reson Imaging (2019) 50:497–510. doi: 10.1002/jmri.26589 30569552

[138] Joo S Ko ES Kwon S Jeon E Jung H Kim JY. Multimodal Deep Learning Models for the Prediction of Pathologic Response to Neoadjuvant Chemotherapy in Breast Cancer. Sci Rep (2021) 11:1–8. doi: 10.1038/s41598-021-98408-8 34552163 PMC8458289

[139] Yang Y Yang JC Shen L Chen JJ Xia LL Ni BB. A Multi-Omics-Based Serial Deep Learning Approach to Predict Clinical Outcomes of Single-Agent Anti-Pd-1/Pd-L1 Immunotherapy in Advanced Stage Non-Small-Cell Lung Cancer. Am J Trans Res (2021) 13:743–+. PMC7868825 33594323

[140] Lv WB Ashrafinia S Ma JH Lu LJ Rahmim A. Multi-Level Multi-Modality Fusion Radiomics: Application to Pet and Ct Imaging for Prognostication of Head and Neck Cancer. IEEE J Biomed Health Inf (2020) 24:2268–77. doi: 10.1109/Jbhi.2019.2956354 31804945

[141] Amini M Nazari M Shiri I Hajianfar G Deevband MR Abdollahi H. Multi-Level Multi-Modality (Pet and Ct) Fusion Radiomics: Prognostic Modeling for Non-Small Cell Lung Carcinoma. Phys Med Biol (2021) 66:205017. doi: 10.1088/1361-6560/ac287d 34544053

[142] Yan JL Toh CH Ko L Wei KC Chen PY. A Neural Network Approach to Identify Glioblastoma Progression Phenotype From Multimodal Mri. Cancers (2021) 13:2006. doi: 10.3390/cancers13092006 33919447 PMC8121245

[143] Mariscotti G Durando M Tagliafico A Campanino PP Bosco D Casella C. Preoperative Breast Cancer Staging With Multi-Modality Imaging and Surgical Outcomes. Eur J Radiol (2020) 122:108766. doi: 10.1016/j.ejrad.2019.108766 31809942

[144] Peeken JC Asadpour R Specht K Chen EY Klymenko O Akinkuoroye V. Mri-Based Delta-Radiomics Predicts Pathologic Complete Response in High-Grade Soft-Tissue Sarcoma Patients Treated With Neoadjuvant Therapy. Radiother Oncol (2021) 164:73–82. doi: 10.1016/j.radonc.2021.08.023 34506832

[145] Xu YW Hosny A Zeleznik R Parmar C Coroller T Franco I. Deep Learning Predicts Lung Cancer Treatment Response From Serial Medical Imaging. Clin Cancer Res (2019) 25:3266–75. doi: 10.1158/1078-0432.Ccr-18-2495 PMC6548658 31010833

[146] Sharma S Mehra R. Conventional Machine Learning and Deep Learning Approach for Multi-Classification of Breast Cancer Histopathology Images-a Comparative Insight. J Digital Imaging (2020) 33:632–54. doi: 10.1007/s10278-019-00307-y PMC7256154 31900812

[147] Pei LM Jones KA Shboul ZA Chen JY Iftekharuddin KM. Deep Neural Network Analysis of Pathology Images With Integrated Molecular Data for Enhanced Glioma Classification and Grading. Front Oncol (2021) 11:2572. doi: 10.3389/fonc.2021.668694 PMC8282424 34277415

[148] Chen MY Zhang B Topatana W Cao JS Zhu HP Juengpanich S. Classification and Mutation Prediction Based on Histopathology H&E Images in Liver Cancer Using Deep Learning. NPJ Precis Oncol (2020) 4:1–7. doi: 10.1038/s41698-020-0120-3 32550270 PMC7280520

[149] Hu J Cui CL Yang WX Huang LH Yu RS Liu SY. Using Deep Learning to Predict Anti-Pd-1 Response in Melanoma and Lung Cancer Patients From Histopathology Images. Trans Oncol (2021) 14:100921. doi: 10.1016/j.tranon.2020.100921 PMC7595938 33129113

[150] Qu H Zhou M Yan ZN Wang H Rustgi VK Zhang ST. Genetic Mutation and Biological Pathway Prediction Based on Whole Slide Images in Breast Carcinoma Using Deep Learning. NPJ Precis Oncol (2021) 5:1–11. doi: 10.1038/s41698-021-00225-9 34556802 PMC8460699

[151] Wang XX Zou C Zhang Y Li XQ Wang CX Ke F. Prediction of Brca Gene Mutation in Breast Cancer Based on Deep Learning and Histopathology Images. Front Genet (2021) 12:661109. doi: 10.3389/fgene.2021.661109 34354733 PMC8329536

[152] Farahmand S Fernandez AI Ahmed FS Rimm DL Chuang JH Reisenbichler E. Deep Learning Trained on Hematoxylin and Eosin Tumor Region of Interest Predicts Her2 Status and Trastuzumab Treatment Response in Her2+Breast Cancer. Modern Pathol (2022) 35(1):44–51. doi: 10.1038/s41379-021-00911-w PMC10221954 34493825

[153] Arya N Saha S. Multi-Modal Advanced Deep Learning Architectures for Breast Cancer Survival Prediction. Knowledge-Based Syst (2021) 221:106965. doi: 10.1016/j.knosys.2021.106965

[154] Klein S Quaas A Quantius J Loser H Meinel J Peifer M. Deep Learning Predicts Hpv Association in Oropharyngeal Squamous Cell Carcinomas and Identifies Patients With a Favorable Prognosis Using Regular H&E Stains. Clin Cancer Res (2021) 27:1131–8. doi: 10.1158/1078-0432.Ccr-20-3596 33262137

[155] Wang XD Chen Y Gao YS Zhang HQ Guan ZH Dong Z. Predicting Gastric Cancer Outcome From Resected Lymph Node Histopathology Images Using Deep Learning. Nat Commun (2021) 12:1–13. doi: 10.1038/s41467-021-21674-7 33712598 PMC7954798

[156] Shi JY Wang X Ding GY Dong Z Han J Guan Z. Exploring Prognostic Indicators in the Pathological Images of Hepatocellular Carcinoma Based on Deep Learning. Gut (2021) 70:951–61. doi: 10.1136/gutjnl-2020-320930 32998878

[157] Rossi L Bijman R Schillemans W Aluwini S Cavedon C Witte M. Texture Analysis of 3d Dose Distributions for Predictive Modelling of Toxicity Rates in Radiotherapy. Radiother Oncol (2018) 129:548–53. doi: 10.1016/j.radonc.2018.07.027 30177372

[158] Gabrys HS Buettner F Sterzing F Hauswald H Bangert M. Design and Selection of Machine Learning Methods Using Radiomics and Dosiomics for Normal Tissue Complication Probability Modeling of Xerostomia. Front Oncol (2018) 8:35. doi: 10.3389/fonc.2018.00035 29556480 PMC5844945

[159] Liang B Yan H Tian Y Chen XY Yan LL Zhang T. Dosiomics: Extracting 3d Spatial Features From Dose Distribution to Predict Incidence of Radiation Pneumonitis. Front Oncol (2019) 9:269. doi: 10.3389/fonc.2019.00269 31032229 PMC6473398

[160] Adachi T Nakamura M Shintani T Mitsuyoshi T Kakino R Ogata T. Multi-Institutional Dose-Segmented Dosiomic Analysis for Predicting Radiation Pneumonitis After Lung Stereotactic Body Radiation Therapy. Med Phys (2021) 48:1781–91. doi: 10.1002/mp.14769 33576510

[161] Lee SH Han PJ Hales RK Voong KR Noro K Sugiyama S. Multi-View Radiomics and Dosiomics Analysis With Machine Learning for Predicting Acute-Phase Weight Loss in Lung Cancer Patients Treated With Radiotherapy. Phys Med Biol (2020) 65:195015. doi: 10.1088/1361-6560/ab8531 32235058

[162] Liang B Van Y Chen XY Yan H Yan LL Zhang T. Prediction of Radiation Pneumonitis With Dose Distribution: A Convolutional Neural Network (Cnn) Based Model. Front Oncol (2020) 9:1500. doi: 10.3389/fonc.2019.01500 32076596 PMC7006502

[163] Wu AQ Li YB Qi MK Lu XY Jia QY Guo FT. Dosiomics Improves Prediction of Locoregional Recurrence for Intensity Modulated Radiotherapy Treated Head and Neck Cancer Cases. Oral Oncol (2020) 104:104625. doi: 10.1016/j.oraloncology.2020.104625 32151995

[164] Murakami Y Soyano T Kozuka T Ushijima M Koizumi Y Miyauchi H. Dose-Based Radiomic Analysis (Dosiomics) for Intensity-Modulated Radiotherapy in Patients With Prostate Cancer: Correlation Between Planned Dose Distribution and Biochemical Failure. Int J Radiat Oncol Biol Phys (2022) 112(1):247–59. doi: 10.1016/j.ijrobp.2021.07.1714 34706278

[165] Buizza G Paganelli C D’Ippolito E Fontana G Molinelli S Preda L. Radiomics and Dosiomics for Predicting Local Control After Carbon-Ion Radiotherapy in Skull-Base Chordoma. Cancers (2021) 13:339. doi: 10.3390/cancers13020339 33477723 PMC7832399

[166] Hirashima H Ono T Nakamura M Miyabe Y Mukumoto N Iramina H. Improvement of Prediction and Classification Performance for Gamma Passing Rate by Using Plan Complexity and Dosiomics Features. Radiother Oncol (2020) 153:250–7. doi: 10.1016/j.radonc.2020.07.031 32712247

[167] Kakino R Nakamura M Mitsuyoshi T Shintani T Kokubo M Negoro Y. Application and Limitation of Radiomics Approach to Prognostic Prediction for Lung Stereotactic Body Radiotherapy Using Breath-Hold Ct Images With Random Survival Forest: A Multi-Institutional Study. Med Phys (2020) 47:4634–43. doi: 10.1002/mp.14380 32645224

[168] Placidi L Gioscio E Garibaldi C Rancati T Fanizzi A Maestri D. A Multicentre Evaluation of Dosiomics Features Reproducibility, Stability and Sensitivity. Cancers (2021) 13:3835. doi: 10.3390/cancers13153835 34359737 PMC8345157

[169] Liu T Yang LX Lunga D. Change Detection Using Deep Learning Approach With Object-Based Image Analysis. Remote Sens Environ (2021) 256:112308. doi: 10.1016/j.rse.2021.112308

[170] Sirinukunwattana K Domingo E Richman SD Redmond KL Blake A Verrill C. Image-Based Consensus Molecular Subtype (Imcms) Classification of Colorectal Cancer Using Deep Learning. Gut (2021) 70:544–+. doi: 10.1136/gutjnl-2019-319866 PMC7873419 32690604

[171] van Timmeren JE Cester D Tanadini-Lang S Alkadhi H Baessler B. Radiomics in Medical Imaging-”How-to” Guide and Critical Reflection. Insights into Imaging (2020) 11(1):1–61. doi: 10.1186/s13244-020-00887-2 32785796 PMC7423816

[172] Pan Y Lei XJ Zhang YC. Association Predictions of Genomics, Proteinomics, Transcriptomics, Microbiome, Metabolomics, Pathomics, Radiomics, Drug, Symptoms, Environment Factor, and Disease Networks: A Comprehensive Approach. Med Res Rev (2022) 42(1):441–61. doi: 10.1002/med.21847 34346083

[173] Gu Y Vyas K Shen ML Yang J Yang GZ. Deep Graph-Based Multimodal Feature Embedding for Endomicroscopy Image Retrieval. IEEE Trans Neural Networks Learn Syst (2021) 32:481–92. doi: 10.1109/Tnnls.2020.2980129 32310786

[174] Crandall JP Fraum TJ Lee M Jiang LD Grigsby P Wahl RL. Repeatability of F-18-Fdg Pet Radiomic Features in Cervical Cancer. J Nucl Med (2021) 62:707–15. doi: 10.2967/jnumed.120.247999 PMC8844259 33008931

[175] Staal FCR van der Reijd DJ Taghavi M Lambregts DMJ Beets-Tan RGH Maas M. Radiomics for the Prediction of Treatment Outcome and Survival in Patients With Colorectal Cancer: A Systematic Review. Clin Colorectal Cancer (2021) 20:52–71. doi: 10.1016/j.clcc.2020.11.001 33349519

[176] Ibrahim A Refaee T Primakov S Barufaldi B Acciavatti RJ Granzier RWY. The Effects of in-Plane Spatial Resolution on Ct-Based Radiomic Features’ Stability With and Without Combat Harmonization. Cancers (2021) 13:1848. doi: 10.3390/cancers13081848 33924382 PMC8103509

[177] Mali SA Ibrahim A Woodruff HC Andrearczyk V Muller H Primakov S. Making Radiomics More Reproducible Across Scanner and Imaging Protocol Variations: A Review of Harmonization Methods. J Pers Med (2021) 11:842. doi: 10.3390/jpm11090842 34575619 PMC8472571

[178] Tan WJ Liu P Li XS Liu Y Zhou QH Chen C. Classification of Covid-19 Pneumonia From Chest Ct Images Based on Reconstructed Super-Resolution Images and Vgg Neural Network. Health Inf Sci Syst (2021) 9:1–12. doi: 10.1007/s13755-021-00140-0 33643612 PMC7896179

[179] Orlhac F Frouin F Nioche C Ayache N Buvat I. Validation of a Method to Compensate Multicenter Effects Affecting Ct Radiomics. Radiology (2019) 291:52–8. doi: 10.1148/radiol.2019182023 30694160

[180] Toprak I Toygar O. Detection of Spoofing Attacks for Ear Biometrics Through Image Quality Assessment and Deep Learning. Expert Syst Appl (2021) 172:114600. doi: 10.1016/j.eswa.2021.114600

[181] Su JW Vargas DV Sakurai K. One Pixel Attack for Fooling Deep Neural Networks. IEEE Trans Evol Comput (2019) 23:828–41. doi: 10.1109/Tevc.2019.2890858

[182] Moons KGM Altman DG Reitsma JB Ioannidis JPA Macaskill P Steyerberg EW. Transparent Reporting of a Multivariable Prediction Model for Individual Prognosis or Diagnosis (Tripod): Explanation and Elaboration. Ann Internal Med (2015) 162:W1–73. doi: 10.7326/m14-0698 25560730

[183] Zwanenburg A Lock S. Why Validation of Prognostic Models Matters? Radiother Oncol (2018) 127:370–3. doi: 10.1016/j.radonc.2018.03.004 29598835

[184] Dercle L Henry T Carre A Paragios N Deutsch E Robert C. Reinventing Radiation Therapy With Machine Learning and Imaging Bio-Markers (Radiomics): State-Of-the-Art, Challenges and Perspectives. Methods (2021) 188:44–60. doi: 10.1016/j.ymeth.2020.07.003 32697964

[185] Tobia K Nielsen A Stremitzer A. When Does Physician Use of Ai Increase Liability? J Nucl Med (2021) 62:17–21. doi: 10.2967/jnumed.120.256032 32978285 PMC8679587

