# Supplementary material for: Deep Learning With Radiomics for Disease Diagnosis and Treatment: Challenges and Potential
Source: Front Oncol. 2022 Feb 17;12:773840. doi: 10.3389/fonc.2022.773840 (PMC8891653; doi:10.3389/fonc.2022.773840)
Supplement: Supplementary file 1 [file DataSheet_1.pdf]

Supplementary Material

1 SUPPLEMENTARY DOCUMENTATION

1.1 Data types and pre-processing

Image data refers to medical images received from hospitals, institutions, or publicly available datasets. When collected retrospectively, the relevant raw data is not available, and, therefore, the images must remain reconstructed as-is. Conversely, when gathered prospectively, the raw data are utilized directly for radiomics studies or with reconstruction parameters appropriate for that research. Indeed, clinical reconstruction settings are properties optimized for ease of visual observation and are primarily designed for detection tasks and not for more proper characterization. Non-isotropic images with larger voxel sizes (e.g., ∼4-5 mm) and post-reconstruction by structural smoothing (e.g., averaging or Gaussian filtering processing) are commonly adopted. For radiomics, smaller and isotropic voxel sizes are more suitable because of the convenience of outlining ROI and reducing biased texture calculations. Moreover, filtering without dropout is not required, and pre-processing pipelines for radiomics (e.g., noise removal, correction of partial volume effects) could apply to unprocessed data. Currently, most radiomics investigations rely on review data, and the impact of image capturing parameters and reconstruction algorithms on radiomic analysis demands to be kept in mind when designing investigative methods.

Non-image data derives from electronic medical records (e.g., age, smoking, clinical staging), test results (e.g., pathology and genomic and proteomic tests), and other clinical notes. The information possessed by non-image and image data is different or complementary. Usually, investigators typically retrieve clinical indicators from a patient's medical history and manually enter them into a new dataset to explore clinical factors contributing to improved predictive outcomes. This manual process is error-prone, and the introduction of such raw errors can be very harmful and challenging to troubleshoot afterward. Hence, a well-designed data quality checking strategy is necessary (1).

Image pre-processing means homogenizing the ROI and making the extracted characteristics rotationally invariant to improve their robustness for radiomic analysis (2). Since inter-slice voxel spacing in medical images is often larger than intra-slice volume spaces, resulting in image data being voxel anisotropic, while rotational invariance of feature extraction requires data to be voxel isotropic. Therefore, interpolation is required to make the voxels equally spaced in each direction. Up-sampling or down-sampling is supposed to be the preferred method for interpolation algorithm selection, and there is no explicit recommendation yet. Furthermore, different modalities of image data may demand distinct interpolation methods. For example, CT-based datasets are usually isotropic, while the provided MRI data are generally non-isotropic. The next step is data normalization, which aims to remove pixels in the segmented region that are not within the specified gray value range. CT and PET data are commonly subjected to region re-segmentation to exclude possible confounding factors, such as pixels of air or bone within the tumor ROI. For MRI data with multiple intensity units, filtering by gray level value is impossible, and filters are ordinarily available to eliminate outlier pixel points. Some examples are the rejection of elements outside the range of grayscale averages +/- three times standard deviations. Finally, a discretization operation is routinely run to reduce the effect of noise. Ideally, a balance exists between removing noise and retaining meaningful signatures. Unfortunately, this also indicates that the optimal choice of the image readout pattern is strongly dependent on the data acquisition parameters (3). The current state of the art is to select the appropriate discretization partner based on experience.

1.2 Second- and higher-order texture features

Gray-Level Cooccurrence Matrix (GLCM) features describe the intensity relationship between a pair of voxels (second-order gray-level). The GLCM calculates the strength information of the voxel pair in all directions and distances (usually one voxel). The specified directions involve two horizontal, two vertical, and four diagonal lines in 2 dimensions and 13 different directions from 3 dimensions during the computation. Then, a matrix is created in each direction to count the probability of certain combinations among neighboring voxels. Finally, the measured rich intensity data are condensed into several different evaluation metrics (4). For example, texture entropy reflects the inhomogeneity or randomness of the gray-level; contrast describes the gray-level differences across voxel pairs; correlation shows the degree of linear correlation of the gray-level; contrast moments measure the local strength changes; maximum probability indicates the most important pair of adjacent voxel values.

Gray-Level Size Zone Matrix (GLSZM) features represent the number of adjacent voxels with the same gray-level image. In the GLSZM, the elements correspond to the count of grayscale regions with the same gray-level (rows) and size (columns), which can come from 2D (8 neighboring regions) or 3D (26 adjacent zones). Therefore, the matrices corresponding to uniform and inhomogeneous ROI are different and behave as wide and flat or narrow matrices. The attributes computed from the matrix have large/small and high/low regions highlighted (5), indicating the distribution of major/minor and superior/inferior gray areas, respectively.

Gray Level Distance Zone Matrix (GLDZM) features combine texture and morphological characteristics. GLDZM adds the additional requirement to GLSZM that neighboring voxel regions with the same ROI edge distance are considered variants of GLSZM (6). It also appears in the names of the profiles, e.g., the small distance high grayscale emphasis signature.

Gray Level Dependence Matrix (GLDM) features are the number of connectives in the image at a predetermined distance, reflecting the grayscale dependence (7). The elements of the GLDM consist of the grayscale level i and the j neighboring dependent pixels and the times of their occurrence counted.

Gray-Level Run-length Matrix (GLRLM) Features defined as the duration of continuous voxels with the same gray value in a particular direction of the image. It provides information about the spatial distribution of one or more orientations from 2D or 3D (8). Where the element (i, j) of the GLRLM represents the number of j consecutive gray levels that occur in the given direction i. GLRLM can calculate the percentage of voxels within the ROI, the run emphasis moment and the non-uniformity, characterizing the granularity, the number of long- and short-run, and the distribution of gray levels and run lengths, respectively. These characteristics are highly similar to GLSZM, without valuable complementary information, and their simultaneous application is generally not recommended.

Neighborhood Gray-Tone Difference Matrix (NGTDM) features capture the difference between each and neighboring voxels at a predetermined distance, i.e., the sum of mean gray level values, similar to human perception of the image (9). Coarseness emphasizes the spatial rate of gray intensity variation, i.e., the relatively uniform gray level among the central voxels and adjacent regions with high coarseness values. There is a positive correlation between contrast and overall dynamic range and spatial rate of change, i.e., a considerable interval of gray value vary in the image; busyness focuses on the spatial frequency of intensity variations, i.e., an ROI consisting of a large number of neighboring blocks with different gray values has a high busyness. In addition, texture intensity reflects the strength of voxel value shifts in the image, the higher the complexity of non-uniform images.

Neighborhood Gray-Level Dependence Matrix (NGLDM) features are similar to NGTDM characteristics (10) which study the gray level relationship per voxel with neighboring voxels, and the only distinguishing factor is whether dependencies exist across them. The reliance on NGLDM traits represents apparent and minor, and uniform dependencies reveal the image heterogeneity, homogeneity, and similarity, respectively.

1.3 Automated segmentation models

Recently, automatic segmentation techniques based on deep learning have gained breakthroughs in medical imaging, with dramatic advances compared to traditional segmentation algorithms (11, 12). Deep learning algorithms seek to learn the hierarchical representation of features with the idea of stacking multiple layers, with the output of the previous one serving as the input to the next and resulting in a stratified expression of the input information. The architecture is a multilayer network consisting of input, hidden (multiple), and output tiers, where only the nodes of the adjacent ones are connected, and the nodes of the same and across layers without being linked. Each level can be similar to a logistic regression model.

Convolutional Neural Networks (CNN) is one of the most widely deployed automatic partitioning frameworks in the field of natural images and has demonstrated strong potential in ROI splitting (13, 14). The core structure of convolution and pooling and fully connected layers through which the input image will pass to obtain the desired output (see *Manuscript Figure 2*). As can be seen in the figure, after several convolutions and pooling, the original multi-dimensional data is "flattened" (i.e., converted to a one-dimensional array) and then attached to the fully connected layer. The fundamental nature of the CNN is that low-level learning of superficial characteristics (e.g., textures and edges) is further employed to study higher-level, more abstract traits. As a result, the network allows learning both local and global peculiarities, which is more conducive to focus on subtle changes in the data.

Fully convolutional neural networks (FCN) convert the fully connected layers of a CNN into separate convolutional layers such that all levels are convolved (13). In CNN, the final desired output value is the probability that the input image belongs to each class, a combination of target detection and semantic segmentation. For FCN, semantic level partitioning comes about by recovering the feature map of the final convolutional layer to the size of the input image. Also, FCN addresses the spatial information lost when mapping the characteristic map to a high-dimensional signature vector in the fully connected level of the CNN. The process could take place in two stages: the encoding procedure is to learn the both low- and high-level traits of the input data and generate a fixed-length feature vector; the decoding aims to classify the trait map at the pixel level by upsampling the characteristic map of the final tier and restoring it to the size of the original image.

Moreover, FCN employs a backpropagation algorithm (15) to train the model in the labeled dataset, a supervised learning process. The first step of the backpropagation mechanism is to randomly assign all weights in the network (forward propagation) to predict the input data in the training set. Then, the loss function is used as an error indicator to compare the predicted values with the known and expected output results and back-propagate all nodes' mistakes.

Meanwhile, the process repeated after updating the corresponding node weights based on the error signal until the model accuracy reaches an acceptable range.

A large proportion of FCN-like algorithms that were applied successfully in the medical image came from well-established classification networks in computer vision, such as AlexNet(16), VGG (17), GoogleNet (18). Over the past decade, the improvement of algorithmic computational efficiency and hardware power, especially the widespread availability of GPU, has accelerated their application in 3D volumetric medical data (e.g., enhanced CT, PET, MRI). For example, the U-Net (19) architecture, broadly utilized for medical image segmentation, forms a U-shaped structure with four downsampling and four upsampling times, respectively, utilizing the previously mentioned encoder-decoder framework. Jin et al. (20) introduced shallower convolutional layer features (higher resolution and shallow layers) using jump connections, allowing to connect different feature mapsĺevels, thus better preserving spatial domain information and learning hallmarks at different levels in the input image. Also, U-Net allows faster and more efficient segmentation in small sample datasets, which is very suitable for the medical image of clinical annotation. Zhao et al. (21) proposed V-Net based on U-Net, introducing a new objective function (Dice coefficient) to optimize the training, which can well handle the case of severe imbalance between the number of foregrounds and background voxels. In addition, DeepMedic is a multiscale two-channel 3D CNN architecture originally intended for lesion segmentation in brain MRI (22). Looney et al. (23) implemented a placental segmentation of 3D US volumes, suggesting that DeepMedic is a proper architecture for cross-modality 3D data. Recently, Yang et al. (11) exploited a combination of 3D FCN and RNN to complete the segmentation of multiple targets, including the fetus, gestational sac, and placenta, in US data. They employed a serialization strategy to resolve blurred boundaries in the images and a hierarchical depth-supervised mechanism to improve the information flow in the network, thus increasing the performance of the segmentation. Similarly, Fiorentino et al. (24) presented a cascaded FCN framework to segmentation fetal head and abdomen in 3D US. Thus, the FCN and CNN frameworks exhibit excellent capabilities in the task of segmenting various tissues (e.g., thorax, abdomen, pelvis, head and neck, brain) across diverse imaging (e.g., CT, US, FDG-PET, MRI), which contributes to the automatic localization of ROIs in radiological analysis.

1.4 Deep learning models

1.4.1 Generative models

1.4.1.1 Generative Adversarial Networks (GAN)

GAN is the best promising generative unsupervised architecture in recent years (25). The network exploits mutual game learning between two independent neural networks, namely the generator and the discriminator, to generate the desired output. The task of the generator model is to learn the input data and make its synthetic data closer to the actual data to “fool“ the discriminator. The discriminator model finds the “fake“ data produced by the generator, distinguishes the differences between them, and discriminates the true from the false. During the training process, an adversarial relationship exists between the two, with the ultimate goal of maximizing the accuracy of the pseudo-samples created by the generator.

1.4.1.2 Deep Belief Networks (DBN)

DBN consist of multiple stacks of restricted Boltzmann machines (RBM), allowing the construction of network architectures of arbitrary depth (26). In the unsupervised learning process, the first layer of RBM to be trained first with the initial observation data, the abstract representation of the input data obtained after the first layer of training as input to the second level (new observation data), and forwards until the last tier of RBM, which eventually generates a joint distribution between observation data and labels. In order to improve the ability of DBN to distinguish other individual differences, a supervised training process might is available to optimize the parameters. Since medical images typically lack data with annotations, a combination of supervised and unsupervised methods is typically considering.

1.4.1.3 Variational Auto-Encoder (VAE)

VAE is a variant of AutoEncoder (25). It differs by adding statistical information that the feature vectors represent Gaussian distributions so that the input data become encoded as characteristic vectors with spatial continuity. After sampling from the signature space, the decoder restores the input data to the same size. In short, the modified encoder generates a large number of latent vectors that follow a unitary Gaussian distribution and then adjusts the loss function containing the latent vectors, the mean square error between the input and output, and the Kullback-Leibler scatter of the Gaussian distribution to fit the original data and give a well-structured trait space.

1.4.2 Discriminative models

1.4.2.1 Convolutional Neural Networks (CNN)

The underlying principles of CNN were already present in Section 1.3. Besides applying segmentation tasks, CNN also perform well in classification problems on multi-modality images (e.g., CT, pathological tissue images,

US) (27–29). Unlike radiomics with automatic segmentation ROI, which employs learned characteristics directly for classification without feature filtering and classifier classification. For example, Chaunzwa et al. (30) built a CNN-based 3D model on CT images of NSCLC patients that significantly differentiated between low- and high-risk mortality groups. Khosravi et al. (31) employed CNN-based network architecture over histopathology images of lung, bladder, and breast cancers with 100% and 92% accuracy in classifying cancer types and two subtypes of lung cancer, respectively. Zhou et al. (32) developed an automatic model for diagnosing benign and malignant thyroid nodules with CNN, and the screening accuracy of benign and malignant nodules on US images was 96%, outperforming other deep learning models.

Furthermore, CNN displays strong potential in medical image registration (33) and reconstruction (34) tasks. Gehlot et al. (35) presented a CNN regression method to solve slow computation and small capture range in 2D/3D image registration. Compared with the intensity-based method, the proposed approach markedly expands the catch scope and reduces the memory occupation. Luo et al. (36) addressed the issue of high-quality image reconstruction of severe artifact data in low-dose X-ray CT utilizing a CNN-based wavelet transform algorithm to suppress specific CT noise and demonstrated its superiority over existing model-based denoising schemes.

1.4.2.2 AutoEncoder (AE)

VE is an unsupervised network structure made of two modules, encoding, and decoding, which can pattern any neural network architecture (37). The encoder learns the hidden features of the input data. At the same time, the decoder utilizes the studied characteristics to reconstruct new ones of the same size as the original data and then trains the encoding and decoding parameters in the network by minimizing the divergence from the original to generated data. When training is complete and random to enter, the decoder can generate approximate data similar to PCA but with better performance. Except for reducing the dimensionality of the input data and acting as a feature extractor for supervised models, it is precious for lack of labeled or sparse data. There exist applications in representation learning, classification, and image denoising (30, 37, 38).

1.4.2.3 Multilayer Perceptron (MLP)

MLP is a feedforward neural network containing one or more hidden layers known as a fully connected neural network (39). It is a forward propagation, where the input data is transferred unidirectionally from the input layer to the output one and can have plural hidden levels. The more tiers a neural network has, the more capable of expressing complex patterns and the higher the computational complexity. Moreover, the heterogeneity problem, which single-layer perceptrons cannot solve, is overcome by a continuous nonlinear function and allows for multiple classification tasks of medical images. For instance, Ho et al. (40) employed medical images of head and neck cancer patients to classify benign and malignant lymph nodes and extra-nodal extension (ENE) and revealed that the MLP neural network predicted malignancy and ENE with 84% and 77% accuracy, respectively.

1.5 Interpretability of depth features

The features learned by deep learning methods are more relevant to the clinical outcomes and data itself than the manually pre-defined characteristics. As seen in *Manuscript Section 2*, radiomic traits from selected external tools or internally developed programs extracted without considering clinical consequences and data attributes in the algorithm design. As a result, such manually defined hallmarks describe general information about the ROI (e.g., shape, the frequency distribution of voxel intensities, and gray levels) and lack specificity about clinical findings, such as conventional radiomics approaches may be less suitable for depicting intra-tumor heterogeneity. In comparison, deep learning algorithms automatically learn depth features from a specific dataset, are more targeted to clinical results and data, and focus more on the variability within the data. In general, the convolutional layer has hundreds of filters to learn certain specific traits of an image. The shallow filters learn low-level visual signatures, while the higher-level filters learn high-level abstract peculiarities, resulting in a multi-level organization of information. As shown in *Manuscript Figure 2*, the first convolutional level studies the voxel intensity information within the ROI, similar to a manually defined first-order histogram feature. The second tier identifies tissue edge information, which corresponds to hand-defined texture properties. The third layers depict more complex morphological information than feature engineering, containing human-defined shape profiles. As layers progress, the convolutional layers define more abstract characters that cannot be interpreted visually but tend to identify heterogeneity within the ROI better. Thus, without the requirement of pre-designed feature formulas, deep learning algorithms extract features containing artificially designed ones and recognize higher-level more abstract characteristics. However, it is also because of the abstract nature of depth features that the models are not very interpretable, and only the results are known, but no reasonable clinical explanation could come out, which is one of the main obstacles to the translation of research results. In addition, the idea that deep neural networks are susceptible to “fooling,“ as confirmed by many investigations (41–43), makes the explainability of the results possibly more critical than the performance of the model.

Although impressive results have emerged in deep learning interpretability research, most of them are limited to specific models that are not readable and understandable for physicians, medical researchers, and patients, restricting

their applications in medical imaging. Nevertheless, in recent years, deep learning interpretability of medical images has attracted attention, which we will describe in the below aspects:

1.5.1 Visualize the lesion area

The black-box nature of deep neural networks lies mainly in the lack of knowledge of how the network organizes, integrates, and interprets image information and the high degree of nonlinearity of the internal hidden layers. The deconvolution method (44) is a popular interpretable technique that provides a way to visualize the interior of a convolutional network by visualizing the convolutional filter and the intermediate hidden levels through an inverted process of inverse pooling-inverse activation-inverse convolution. The lower convolutional tiers focus on tumor edges and texture characteristics, while the higher ones correspond to tumor shapes (e.g., high response to circular or curved shapes). Additionally, lesion regions are visualized primarily by heatmaps (45, 46) and attentional mechanisms (47, 48) to identify suspicious fields and provide a visual basis, such as Khorrami et al. (46) presents the results of unsupervised clustering with radiomic features using heatmap cluster gram. This approach localizes or quantifies doubtful areas of disease in medical images, which can enhance the perception of internal representations of deep learning models and potentially provide a lesion location.

1.5.2 Semanticized results

Concerning the black box problem of deep learning, outcome semanticization simulates a physician's consultation process by generating comprehensible determination procedures and outcomes while outputting diagnostic results, e.g., primary diagnosis reports. That requires the introduction of medical knowledge in the predictive model to explain the semantic concepts of neurons in the neural network. Pulvermuller et al. (49) describes network profiling methods that attempt to establish connections between network neurons and visual semantic notions. Currently, few studies are associated with neurons, mostly combining different types of data such as medical history information and test records into the medical image handling process, and finally generating the corresponding diagnostic statements with an understandable basis for judgment. For example, Wang et al. (50) exploited a multi-level attention mechanism to combine prior clinical knowledge and chest X-rays to annotate the dataset automatically. The results revealed that the TieNet network produced significant disease classification and initial write-up. In terms of interpreting medical image information, Shen et al. (51) evaluated the benignity and malignancy of lung nodules on chest CT in an expert manner by low-level semantic characteristic simulation, demonstrating superior findings over CNN. Thus, the profound fusion of distinct deep learning models with medical knowledge can explain the diagnostic yield and provide clinicians with a referenceable basis for decision-making.

1.5.3 Data causal inference

Regarding logical inference, the logical association of medical image interpretability lies in the causal connection of the data incorporated in the model design phase, but no study has yet indicated which factors are causally bound to the diagnostic outcome. In a study of diabetic retinopathy, Niu et al. (52) drew on the well-known Koch's postulates in epidemiology to link pathological descriptors to lesions and discovered that images synthesized by GAN had lesion-related symptoms. The construction of medical images and medical diagnosis knowledge graphs is beneficial to improve the matching ability and logical reasoning of models. Unfortunately, the integration with medical knowledge is not enough, and only a few related types of research require further exploration.

Despite the promising results of deep learning interpretability research, it is still in the infancy stage and will have a long way to go in the future.

1.6 Study design compliance

From a legal perspective, the recent discussion of AI liability in the EU General Data Protection Regulation states that "improving the interpretability of models is an essential aspect,“ has received attention from policymakers and has shown its importance even more in the context of sensitive applications. Currently, AI-based applications require interpretation at the legal level and, most likely, will soon require a basis for interpretation. From an ethical standpoint, the Hippocratic Oath applies AI systems to clinical settings, and enforcing it is an issue that cannot ignore. Indeed, since the data collection and modeling process itself is biased by considering specific types of patients, it is necessary to carefully consider the ultimate beneficiaries and stakeholders of AI technologies are? Algorithms designed and hidden for commercial purposes are unethical (53) and may exacerbate existing tensions between care and profitability. In addition, a conscious effort to avoid a high reliance on AI and the abandonment of medical knowledge in the automation process leads to ”learned helplessness.” At the same time, automated diagnostic models may pose challenges to accountability and confidentiality in the doctor-patient relationship.

In terms of regulation, the US Food and Drug Administration began regulating computer-aided diagnostic systems decades ago. As new AI algorithms evolve and data are continuously updating, clinical applications seeking regulatory approval must scrutinize the authenticity of training data, predictive performance, generalizability and robustness, and the ability to learn from new data. As a result, new AI applications submitted for regulatory approval may need to

meet rigorous tests, including quality control and risk assessment. The vast amount of healthcare data that has become part of Big Data provides a fertile environment for developing state-of-the-art AI systems. However, it also poses considerable challenges for data security and privacy. Federated Learning currently offers a distributed solution (54) that allows data sharing across countries or centers while preserving data privacy.

2 SUPPLEMENTARY TABLES

Table S1: Overview of general feature extraction and analysis toolkits

Packages	Imaging	ROI tools	Image pre-processing	Radiomic features	IBSI
S-IBEX	CT, MR, PET	Free and Point draw, Nudge	Interpolation, Re-segmentation, Discretization; Image (smoothing, enhancement, deblur), Change enhancement, Resample	109: Morphology, Intensity-based statistics and histogram, GLCM, GLRLM, GLSZM, GLDZM, NGTDM, NGLDM	IBEX:not meet,S-IBEX: meets
PyRadiomics	CT, PET, MR, US, etc.	None	Filters: Wavelet, Laplacian of Gaussian, Several simple (square, square root, logarithm, exponential)	120:19 First-order statistics, Shape (2D:10+3D:16), 24 GLCM, 16 GLRLM, 16 GLSZM, 14 GLDM, 5 NGTDM	Meets
CGITA	Designed for PET, CT, MR	Region growth,Fuzzy C-means	None	72: GLCM, GLSZM, NGLDM, Normalized cooccurrence and Voxel-alignment matrix, Voxel statistics, Texture spectrum	None
Mazda	Designed for MR	Thresholding, Deformable surface	Resample, Discretization, Normalization	279:Statistical, Model-based (fractal, stochastic), Image transform (Fourier, Gabor, Wavelet)	Not meet,bad match with IBEX
CERR	CT, PET, MR, US	Contouring tools, Segment-labeler	Filters: Wavelet, Sobel, Gabor, Laplacian of Gaussian	First-order/histogram statistics, Intensity-volume histogram, Peak/Valley, Shape, Size, GLCM, NGTDM, NGLDM, GLRLM, GLSZM	Meet; Match with PyRadiomics
RadiomiX	CT, PET, MR	None	Discretization, Interpolation: Wavelet Laplacian of Gaussian Resampling	543: Fractal, First order statistics, Shape and size, GLCM, GLRLM, GLSZM; Wavelet decomposition	Partial meet
TexRAD	CT, PET, MR	Manually draw, Thresholding	Filters: Laplacian of Gaussian(Scale: 0mm, 2mm, 4mm, 6 mm)	230: Histogram and statistical; Mean value, Entropy, Kurtosis, Skewness, et.	None
QIFE	CT, MR, PET, US, ,etc.	None	Segmentation deformation, Topology preservation, Maximum connected volume velection, Hole filling	Size and intensity, Edge sharpness, Local volume invariant integral, Surface roughness, Sphericity, GLCM	None

IBSI=Image Biomarker Standardization Initiative.

Table S2: Overview of commonly utilized feature selection methods and classification models

Feature selection methods	Acronym	Classifiers and Evaluation	Acronym
Filtering methods		Supervised learning models	
Univariate analysis		Support vector machine	SVM
Relief	RELF	Logistic regression	LR
Wilcoxon rank sum	WLCR	Multiple logistic regression	MLR
Chi-square test	CHST	Bagging tree	BAT
T-test	TET	Boosting tree	BOT
Spearman correlation	SPC	Gradient boosting tree	GBT
Spearman rank correlation	SPRC	Dual-channel bidirectional long and short-term memory	DC-BiLSTM
Mann–Whitney U test	MWUT	Multivariate Cox proportional hazards regression	MCOX-PHR
Pearson linear correlation coefficient	PLCC	LASSO-logistic regression	LASSO-LR
Distance correlation coefficient	DCC	Decision trees	DT
False Discovery Rate correction	FDRC	Random forest	RF
Fisher's exact test	FET	Multivariate random forest	MRF
McNemar's test	MNT	Linear discriminant analysis	LDA

Continued on next page

Table S2 — Continued from previous page

Feature selection methods	Acronym	Classifiers and Evaluation	Acronym
ANOVA	−−	Recursive feature elimination	RFE
Sparse representation coefficient	SRC	Naïve Bayes	NAB
Orthogonal matching pursuit	OMP	K-nearest neighbor	KNN
Kruskal–Wallis H test	KWHT	Extremely randomized trees	ERT
Shapiro-Wilk test	SWT	Least-squares regression	LSR
X^2 test	XET	Quadratic discriminant analysis	QDA
Gini index	GINI	Random survival forest	RSF
Information gain	IFGN	Multiple Random survival forest	MRSF
Euclidean distance	EUDT	Support vector machine with radial basis function kernel	SVM-RBF
Joint mutual information	JMI	Fisher's linear discriminant	FLD
Multivariate Analysis		Linear kernel and radial basis function	RBF
Minimum redundancy maximum relevance	mRMR	Artificial neural network	ANN
Mutual information	MUIF	Unsupervised learning models	
Mutual information maximization	MIM	K-Means clustering	KMC
Conditional mutual information maximization	CMIM	Fuzzy clustering	FUC
Mutual information feature selection	MIFS	Fonsensus clustering	FOC
Mean of Feature Ratio	MFR	Semi-supervised learning model	
Wrapping methods		Based on deep learning	−−
Recursive feature elimination	RFE	Other technologies	
Las Vegas Wrapper	LVW	Deep learning	DL
Embedding methods		Extreme learning machine	ELM
Least absolute shrinkage and selection operator	LASSO	Model Evaluation	
ElasticNet	ELN	Hosmer lemeshow test	HLT
XGboost	XGBT	Harrell's C-index	HCI
Ridge regression	RIR	Akaike's information criterion	AIC
Decision Trees	DT	Matthews correlation coefficient	MCC
Random Forest	RF	Log-rank test	LRT
Gradient Boosting Tree	GBT	DeLong test	DLT
Random logistic regression	RLR	Relative standard deviation	RSD
support vector machine-based recursive feature elimination	SVM-RFE	Receiver operating characteristic	ROC
Unsupervised methods		Area under curve	AUC
Principal component analysis	PCA	Calibration plot	CAP
Independent component analysis	ICA	Kaplan-Meier curves	KMC
Isometric mapping	ISOMAP	Heatmaps	−−
Cluster analysis	CA	Nomogram	−−
Locally linear embedding	LLE	−−	−−

Table S3: Application of deep learning with radiomics studies in different modalities

**Study;Dataset; Total:Train+Test**	**Modality;Patient; Predictive;Method**	**Features (Clinical;Radiomic;Predictive)**	**Model (Construct;Statistics)**	**Predictive performance(%); Application Type**
**Conventional radiological images**				
Li et al. (55); 2 Centers; 1985: *	Mammographic; **Breast cancer**; Mammographic density classification; DL	–; Depth features; high-order, high-abstraction, and subtle features	ResNet50 with DC (RNDCCA) & 4 Inputs with DC & CA (4IDCCA) & ResNet50, ResNet34, ResNet101, Multi-View inputs (2, 2 or 4), DC and CA	RNDCCA (ACC, AUC, F1-score): 88.7, 97.4, 87.1; 4IDCCA: 90.3, 97.0, 89.2; **Diagnosis**

Continued on next page

Study;Dataset; Total:Train+Test	Modality;Patient; Predictive;Method	Features (Clinical;Radiomic;Predictive)	Model (Construct;Statistics)	Predictive performance(%); Application Type
Kolossvary et al. (56); 1 Hospital; 300: *	CT; **Coronary artery disease (CAD)**; Development of CAD; Radiomics	Cardiovascular risk factor (11); 1276; 13 separate significant clusters	Multivariable and cluster analysis, Robustness of clusters; Similarity matrix and cluster dendrogram, Bar chart	Conventional risk factors, cocaine use, and HIV infection are critical factors for the progression; **Diagnosis**
Benedetti et al. (57); 1 Hospital; 39: *	ceCT, non-ceCT; **Pancreatic neuroendocrine tumors**; Identifying histopathologic features; Radiomics	grade, microscopic vascular infiltration, .etc (17); 69 (First- second- and higher-order statistics features); non-ceCT (17), ceCT (18)	Radiomic analysis; Spearman coefficient, Mann–Whitney test with Bonferroni	Several radiomic features can discriminate histopathology; **Diagnosis**
Pang et al. (29); 1 Center; 1447: 1158 + 289	US; **Breast masses**; Benign vs. malignant; DL	–; Depth features; *	Semi-supervised Inception-V3, TGAN; 5-F CV, AlexNet, VGG, ResNet	AUC 90.41, SEN 87.94, SPE 85.86; **Diagnosis**
Jiang et al. (58); 1 Hospital; 2056: *	MR; Early-stage **cervical cancer**; Vessel invasion; DL	–; Depth features; *	Attention ensemble learning; VGGNet, Inception-v3, ResNet-v2, DenseNet121, VGG19-CBAM, VGG19-SE, 10-F CV, Heatmaps	ACC 81.4, AUC 91.1, SEN 88.1, SPE 75.2; **Diagnosis**
Meng et al. (45); 4 Hospitals; 539:377+162	CT; **Gastric cancer**; Lymph node metastasis (LVM), Lymphovascular invasion (LVI), T stage; Radiomics	7; 867(First-order, GLCM, GLRLM, GLSZM, GLDM, NGTDM); LVM: 2D(7) 3D(6), LVI: 2D(3), 3D(2), pT4 or other pT stage: 2D(7) 3D(5)	LRM, SVM; ICC, Select (MWUT, PLCC, mRMR, LASSO), 5-F CV, Heatmaps	LNM (AUC): 2D(73.1), 3D(72.2), LVI: 2D(69.9), 3D(73.2), pT: 2D(73.1), 3D(72.9); **Diagnosis**
Xie et al. (59); 1 Hospital; 74: *	CT; **Malignant pleural mesothelioma**; BAP1 mutation; Radiomics	morphological (10); 1316 (32 First-order, 74 Texture, 744 Wavelet, 186 LoG); 3D(4), 2D (2)	LR; Univariate and multivariate analysis, SMOTE, MWUT, WLCR, 1000-times bootstrap	3D-based radiomic features have better predictive power than 2D; **Diagnosis**
Du et al. (60); 1 Hospital; 548: 438 + 110	US; **Pregnant women**; Fetal lung development; Radiomics	10; 430 (15 Morphological, 73 Texture, 342 Wavelet); 150-300	SVM; Select (SRC, OMP), 5-F CV	AUC 95-99, ACC 80.6-86.4, SEN 74.5-91.3, SPE 75-88; **Diagnosis**
Ligero et al. (61); 1 Hospital; Cohort 1-3 (115, 67, 63)	CT; Advanced Solid Tumors **(breast, cervix, gastrointestinal)**; Anti–PD-1/PD-L1 Response; Radiomics	9 types; First- second- and higher-order statistics features; 26 (ICC >0.7), 14 (Elastic-net model)	MCOX-PHR; LR, MWUT, AIC, HLT, Kaplan–Meier, 10-F CV	Radiomics (AUC, 95% CI): 70.0, [0.64, 0.77], Radiomics-clinical: 74.0, [0.63, 0.84]; **Diagnosis**
Rossi et al. (62); 3 Centers; 170	CT; **NSCLC**; EGFR; Radiomics	2; 104 (First-order, GLCM, GLDM, GLRLM, GLSZM, NGTDM); 42	SVM; Select (MFR, PCA), Test-Retest, 5-F CV	AUC 85, ACC 88.1; TKIs: 17 RFs associated with T790M; **Diagnosis**
Agazzi et al. (63); 1 Hospital; 84	CT; **NSCLC**; EGFR mutation and ALK rearrangement; Radiomics	Age, Smoking, ALK, and EGFR status; 29 (CT texture fetures by radiologists experienced); 17	Generalized boosted regression model (GBM); SMOTE, mRMR, ANOVA, 10-F CV	ACC 81.76, 95 CI [81.45–82.06]; Noninvasive characterization **Diagnosis**
Choi et al. (64); 3 Centers; 1166: 856 + 310	MR; **Gliomas**; IDH mutation; Radiomics + DL	*; 3Dl 6 Loci, 3D 13 shape and depth features; unclear	Hybrid model (Model 1 & 2); Model 1 (3D U-shaped CNN), Model 2 (34-layer ResNet), Ablation analysis (conducted and saliency maps), DC	3 datasets: ACC (93.8, 87.9, 78.8), AUROC (96, 94, 86), AUPRC (88, 82, 81); **Diagnosis**
Wu et al. (65); 3 Centers; 297:229+68	CT;**Lung adenocarcinoma**;Invasive adenocarcinoma;Radiomics	25; *; SEM(7), GTV region (16)	GTV Model; ComBat, Select (WLCR, SPC, Boruta), DLT, ICC	AUC 98, ACC 93, SEN 98, SPE 78; **Diagnosis**
Liu et al. (66); 2 Hospitals; 148:111+37	PET/CT; **Lung adenocarcinoma**; EGFR; Radiomics	4; CT (1470), PET (100); CT (8), PET (2)	Xgboost; Select (MWUT, MRF, lR)	AUC: EGFR-Mutant 87, Exon19 77, Exon21-L858R 92; **Diagnosis**
Zhang et al. (67); 1 Hospital; 248:175+73	PET/CT; **Lung adenocarcinoma**; EGFR; Radiomics	5; CT (45), PET (47); Maximum, SHAPE, GLCM, GLRLM, NGLDM, GLZLM	MLR; Select (MWUT, TET, CHST, LASSO), 10-F CV, HLT	AUC 87, ACC 80.82, SEN 91.67, SPE 70.27; **Diagnosis**
Mu et al. (68); 4 Hospitals; EGFR, TKIs, ICI: 1232, 67, 149	PET/CT; **NSCLC**; EGFR, Treatment response; DL	7; Depth features; *	DL Score (DLS); Select (MNT, WLCR), Model (MLR), DLT, Heatmaps	EGFR: AUC 79; DLS with PFS: Strong positive (TKIs) and negative (ICI) correlations; **Diagnosis**
Haubold et al. (69); 1 Hospital; 42:*	PET-MRI; Suspected primary **brain tumor**; Mutation; Radiomics	*; 19284; 8-64 features	Linear SVM, RF; Select (TET, ANOVA, CHST, RLR), 3-F CV	AUC: ATRX 85.1 MGMT 75.7 IDH1 88.7 1p19q 97.8; **Diagnosis**
Jin et al. (70); 1 Hospital; 172: 100 + 72	US; **Cervical cancer**; Lymph node metastasis (LNM); Radiomics	3;152(2 Shapes,7 Size,15 Histogram,128 Texture);3 GLCM, 1 GLRLM, 2 GLZLM	LR; Select (MWUT, FET, CHST, ELN, LASSO, RIR)	AUC 77; 6 features significantly associated with LNM; **Diagnosis**
Peng et al. (71); 1 Hospital; 805: *	US; Primary **liver cancer**; Histopathological Subtypes; Radiomics	16; 5234 (First-order, Shape, Wavelet, Texture); Hepatocellular carcinoma (HCC): 16, Intrahepatic cholangiocarcinoma (ICC): 19	LR; ComBat, TET, MWUT, KWHT, SVM-RFE, SPC, LASSO, RF, LR, MLR, mRMR, DT, NAB, KNN, LR, SVM, BAT, RF, ERT, ADB, GBT, Z-score	HCC vs non-HCC AUC: Train 85.4, Test 77.5; ICC vs HCC-ICC AUC: Train 92.0, Test 72.8; **Diagnosis**
Dohan et al. (72); 9 Centers; 230:120+110	CT; **Colon cancer** treated with FOLFIRI and bevacizumab; Treatment response; Radiomics	10; 114; Univariate analysis (20), Multivariate analysis (3)	MCOX-PHR; LRT, Kaplan-Meier, SpectraScore	3 OS significant features could identify patients with good response within 6 months; **Response**
Chen et al. (73); 3 Centers; internal (595: 355 + 118), external (122)	ceCT; **Hepatocellular carcinoma**; Response to first transarterial chemoembolization (TACE); Radiomics	27 items; 3404 (851 x 4ROI); 18	Multivariable LR, MCOX-PHR; LASSO, AIC, 10-F CV, Nomogram, Calibration curve, Kaplan-Meier, LRT	Subclassification: AUC 90; Objective response: hazard ratio:2.43, 95% CI: 1.60–3.69, p >0.001; **Response**

Continued on next page

Table S3 — Continued from previous page

Study;Dataset; Total:Train+Test	Modality;Patient; Predictive;Method	Features (Clinical;Radiomic;Predictive)	Model (Construct;Statistics)	Predictive performance(%); Application Type
Dissaux et al. (74); 4 Centers; 87: 64 + 24	PET/CT; **NSCLC** with SBRT; Local recurrence (LC); Radiomics	6; 92; PET (IC2, Strength), CT(IC2, Flatness)	MCOX-PHR; ComBat, Select (SPRC), DLT, Youden index, Kaplan–Meier	SEN 100, SPE 88; 2 PET features related to LC; **Response**
Fatima et al. (75); 2 Centers; 214 (Recurrence 17, Non 34): *	US; RT treated **head-neck squamous cell carcinoma**; Recurrence; Radiomics	10; 7 quantitative, 24 textures; delta-radiomics (Week-1 2, Week-4 4)	SVM; Select (CHST, FET, SWT, TET, MWUT, LASSO), LRT, Kaplan Meier	Week-1: AUC 75, ACC 80 Week-4: AUC 81, ACC 82; **Response**
Xiong et al. (76); 1 Hospital; 125: 63 + 62	MRI; **Breast cancers**; NAC insensitivity; Radiomics	8; *; Kurtosis, NGTDM-busyness, GLCM-IMC2, NGTDM-busyness	MLR; Select (WLCR, LASSO), 5-F CV, AIC	AUC 79.2, ACC 87.1, PPV 33.3, NPV 92.86; **Response**
DiCenzo et al. (77); 4 Centers; 82: *	US; **breast cancer**; Treatment response with NAC; Radiomics	6; 31 (GLCM); KNN (3), SVM-RBF (4), FLD (3)	KNN; Select (SWT, TET, MWUT), Model (SVM-RBF, FLD), CV	AUC 72.6, ACC 86.6, SEN 83.3, SPE 72.6, F1-score 87.1; **Response**
Jiang et al. (78); 2 Hospitals; 592: *	US; **breast cancer**; Pathological complete response (pCR) with NAC; Radiomics + DL	13; Radiomic (Histogram, Morphology, Intensity, Laws, Wavelet, Texture), Depth features; ROI-1 (5), ROI-2 (7)	DL radiomic nomogram; Select (FET, CHST, TET, MWUT, LASSO), DLT, HLT, ICC, Calibration curve	AUC 94; Able to predict pCR better; **Response**
Quiaoit et al. (79); 3 Centers; 59: *	US; **breast cancer**; Treatment response with NAC; Radiomics	7; Texture (GLCM); Week-1 3, Week-4 4	SVM-RBF (Week 1, Week 4); Select (SWT, ANOVA, MWUT), Model (KNN, FLD)	AUC, ACC, SPE, SEN, F1-Score: Week-1 87, 81, 79, 83, 81; Week-4 87, 81, 82, 80, 81; **Response**
Hu et al. (80); 2 Centers; 231: 161+70	CT; **Esophageal squamous cell carcinoma**; Treatment response to neoadjuvant chemoradiotherapy; Radiomics, DL	11; 851 (first-order, shape, and second-order features included GLCM, GLRLM, GLSZM, GLDM, and NGTDM): 107 (original) and 744 (wavelet); depth features	ResNet50; Xception, VGG16, VGG19, InceptionV3 or InceptionResNetV2, SVM, ComBat harmonization, FET, KWHT, HLT;	ResNet50 (best): ACC 77.1, AUC 80.5, 95% CI [0.696–77.9], radiomics: ACC 67.1 ,AUC 72.5, 95% CI [0.549–0.913]; **Response**
Haider et al. (81); 1 Center; 190: *	PET/CT; **Oropharyngeal squamous cell carcinoma**; Post-radiotherapy locoregional progression; Radiomics	12; 1037 (PET), 1037 (CT): n=11 (14 shape, 18 first-order, 75 texture-matrix);*	RF + clinical model; Random survival forest, RF model, clinical model, 5-F CV, HCI, LRT, Risk stratification and Kaplan-Meier;	C-index: 76, log-rank p-values of 0.003, 0.001, 0.02, 0.006 in Kaplan-Meier analysis; **Prognosis**
Zhao et al. (82); 27 Centers; 87: 61 + 26	MRI; Brain metastases of ALK-positive **NSCLC**; PFS, High and low risk stratification; Radiomics	5; 396 (Histogram 42, GLCM 144, GLRLM 180, Haralick 10, Morphological 9, GLZSM 11); 9	LASSO-LR, R-cores; Select (mRMR, CHST, FET, MWUT, LASSO), 10-F CV, Kaplan-Meier	Intracranial progression within 51 weeks: AUC 85; PFS (R-scores): high- vs. low-risk (P=0.017); **Prognosis**
Ferreira et al. (83); 3 Hospitals; 158: 80% + 20%	PET/CT ; **Cervical cancer**; Disease-free survival Radiomics	4; 215 (first-order, geometry, fractals, texture matrix and others); 3 (clinical), 6 (radiomic)	MCOX-PHR; RF, mRMR, MIFS, GINI, ANOVA, PLCC, Kaplan-Meier curves, LRT, bootstrapped,	AUC 78, F1-score 49, precision 42, recall 63; **Prognosis**
Khorrami et al. (46); 2 Hospitals; 139:50+89	CT; **NSCLC** PD-1/PDL-1; Treatment response, OS; Radiomics	7; 57(Textures, Statistics, Shapes); delta-radiomic features	LDA, MCOX-PHR; Select (WLCR), ICC, Heatmap cluster gram, LRT, Kaplan-Meier	response: AUC 88±8, OS: P(0.0011) C-index(0.72); **Prognosis**
Krarup et al. (84); 2 Hospitals; 233:*	PET/CT; **NSCLC**; Tumor heterogeneity; Radiomics	*; *; GLCM (3), GLRLM(1), GLZLM(1), Shape(1)	MCOX-PHR; Select (SPRC), Uni- and multivariate Cox regression analysis	Selected RFs correlate with PFS and carry prognostic value; **Prognosis**
Liu et al. (85); 1 Hospital; RFA 214: 149+65, SR 205: 144+61	Contrast-enhanced US; **Hepatocellular Carcinoma**; Progression free survival (PFS) for RFA and SR; Radiomics+DL	17; Thousands of features; p ¡ 0.0001 (*)	DL-Based Radiomics Model (Cox-CNN); Select (HLT, MWUT, TET, XET), AIC, Harrell's C-index, Kaplan-Meier survival curves, Nomograms, Visualization	Preoperative 2-year PFS (increase ACC): Radiofrequency ablation (RFA) 12, Surgical resection (SR) 15; **Prognosis**
**Histopathological images**				
Sharma and Mehra (86); BreakHis; 7909: 7118+791	WSI; **Breast cancer**; Benign vs. malignant, Sub-categories 8: benign (4), malignant (4); Pathomics + DL	–; Handcrafted features (HF): color, shape, texture Depth features; Handcrafted (6), Deepth (*)	Linear SVM + VGG16; Pathomic Model (HF + RF, HF + SVM) DL (VGG16, VGG19, ResNet50) + (RF, SVM, LR, KNN, LDA)	Magnification (40,100,200, 400):ACC 93.97 92.92 91.23 91.79; **Diagnosis**
Trivizakis et al. (87); 1 Center; 5000: 4050+50, validation (500)	Histopathological images; **Colorectal cancer**; classify tissue pathology; Pathomics	–; 532: Texture (WPT-LBP, WPT-Gabor filters, WPT-GLCM, WPT-LBP-GLCM, WPT-Gabor-GLCM, and other), first- and higher-order statistics; 6 discrete wavelet texture features	2 SVM and ANN models; 10-F CV, Wavelet packet transform (WPT), Gabor filters, Local binary patterns (LBP), ANOVA	proposed model: ACC: 95.32, recent studies: ACC 87.4; **Diagnosis**
Kim et al. (88); 1 Hospital, 1 public; Hospital 500: 400+100, Center 160 Test	WSI; **Prostate cancer**; Benign vs. malignant, Low-grade vs. high-grade; Pathomics	–;Texture: Energy, Entropy, Skewness, Variance, Kutosis, Uniformity; Energy, Entropy, Skewness, Variance, Kutosis	DC-BiLSTM; Gamma correction, Select (RFE, ANOVA), Model (SVM, LR, BAT, BOT)	ACC, PRE, Recall, F1-score: benign vs. malignant (89.2, 88.7, 90.0, 89.2), low vs. high grade (93.6, 96.3, 91.2, 93.7); **Diagnosis**
Pei et al. (89); 1 Center; 549 (LGG: 201 II + 229, HGG: 119): 80%+20%	WSI; **Glioma**; Classification and grading; DL Pathomics	Diagnoses and molecular information; Depth features; Molecular features, Depth features	DNN and ResNet Models; 5-F CV, Color Normalization of WSI	LGG vs. HGG: ACC 93.81, competitive performance; LGG II vs. III: ACC 73.95, outperform state-of-the-art; **Diagnosis**

Continued on next page

Table S3 — Continued from previous page

Study;Dataset; Total:Train+Test	Modality;Patient; Predictive;Method	Features (Clinical;Radiomic;Predictive)	Model (Construct;Statistics)	Predictive performance(%); Application Type
Li et al. (90); 1 Hospital; 200: 150+50	WSI; **Lung cancer**; Classification and grading; DL Pathomics	–; Depth features; *	DenseNets; Top 10 teams in multi-model and single model methods	Best model: DC 83.98 ± 89.0; Mean DC: 79.66 (Multi-model methods), 75.44 (single model methods); **Diagnosis**
Chen et al. (91); 1 Hospital, GDC-porta; public 377: 284+94, Hos 101	WSI; **Liver cancer**; Hepatocellular carcinoma (HCC), Mutation; DL Pathomics	6; Depth features; *	Inception V3; 3 pathologists, Precision-recall curves, MCC	HCC: ACC 96, F1-score 97.1 (CTNNB1, FMN2, TP53, ZFX4): AUC 71-89; **Diagnosis**
Sun et al. (92); The Cancer Genome Atlas (TCGA); 462: 393+69	WSI; **Liver cancer**; Abnormal vs. normal ; DL Pathomics	–; ResNet-50: Depth features; Patch features aggregated into global labels	Multilayer perceptron (ReLU); Aggregate features: max and mean and 3-norm poolings, MIL technique	ACC 98, F1-score 99, Recall 1; **Diagnosis**
Karimi et al. (93); 1 Hospital; 333: 247+86	WSI; **Prostate cancer**; Benign vs. malignant, Gleason grade; DL Pathomics	–; Depth features; *	LR; 6 pathologists, CNN_{Large}, CNN_{Medium}, $CNN_{Small}(patch)$	Category: ACC 92, Grade 3 vs. Grade 4+5: ACC 86; **Diagnosis**
Woerl et al. (94); 1 Center, TCGA; Center 16, TCGA 363: 327+36	WSI; **Bladder cancer**; Molecular Subtype; DL DL Pathomics	–; Depth features; *	ResNet-based; 4 pathologists, 6-F CV, LRT, Kaplan-Meier, Nanostring analysis, Class activation maps	Center: Mean AUC 89±3 ACC 69.91, TCGA: Mean AUC 85, ACC 75; **Diagnosis**
Wang et al. (95); 1 Center, TCGA; Center 939:585+354, TCGA 500:400+100	WSI; **Lung cancer**; Carcinoma, ADC, SC, SCLC; DL Pathomics	–; ScanNet: Depth features; Patch: AvgFeat, WeightedFeat, MaxFeat Global: MeanPool, Norm3	CNN-WeightedFeat-Norm3-Based-RF, CNN-MaxFeat-MeanPool-Based-RF; Feature visualization: neighborhood embedding (t-SNE)	Center: ACC 97.3 ACC 95.3 TCGA: ACC 82.0 ACC 85.6; Greatly exceeds state-of-the-art methods; **Diagnosis**
McGarry et al. (96); 1 Hospital; Annotation (Single 123, Multi 33)	WSI; **Prostate cancer**; Inter-observer differences; Pathomics	–; *; *	LSR; ANOVA, Krippendorff's alpha, Apparent diffusion coefficient, Radiopathomic mapping	Sensitive to labels annotated by pathologists; Promotes consensus training; **Diagnosis**
Hu et al. (97); 3 Centers; Train:476, validation: 54, 55	WSI; **Melanoma and lung cancer**; Anti-PD-1 response; DL Pathomics	–; 2048; TILs, depth features	VGG-16 + SVM; 10-F CV, 6 DL models (Resnet-50, Inception-V3, VGG-19, Nasnet, Desnet and Mobilenet)	The AUC ranging from 0.55 to 0.71; The best proposed model: 77.8 95% CI [0.638-.0905] (melanoma), 64.5 95% CI [0.494- 0.784] (lung cancer); **Response**
Qu et al. (98); 1 Center; 659 (train, test, validation): 75%, 15%, 15%	WSI; **Breast Cancer**; genetic mutations and biological pathway activities; DL Pathomics	–; Depth features (ResNet-101); *	Multi-layer perceptron (MLP) predictor with self-attention; 1000-times bootstrapping, AUC, ROC, 95% CI, DC	AUC ranges:[68–85] (6 mutations), [69–79] (copy number alteration): [65–79] (pathway activities); **Response**
Wang et al. (99); 2 Hospitals; 222: 110+112	WSI; **Breast Cancer**; gBRCA mutation; DL Pathomics	–; Depth features; *	ResNet; Confusion matrix, DLT, Box plot, bar plot, Bland–Altman plot	AUC, 95% CI(40, 20, 10, and 5 magnification tiles): 76.6, [76.3–76.9], 76.3, [75.8–76.9], 75.0, [73.8–76.1], 55.1 [52.6–57.5]; **Response**
Farahmand et al. (28); 3 Centers; 1241 (188 + 668 + 385): 70%+30%	WSI; **Breast Cancer**; HER2 status and trastuzumab treatment response; DL Pathomics	–; Depth features; *	Inception v3 architecture; 1000-times bootstrap, 5-F CV, heatmap	HER2 status: AUC 81; classifier pre-treatment samples received trastuzumab therapy; **Prognosis**
Arya and Saha (100); 1 Center; 1980	3 modalities; **Breast Cancer**; survival prediction; DL Pathomics	25 clinical features; 3 modeatiles (clinical profile, CNA profile, and gene expression profile); SiGaAtCNN features; STACKED features	Sigmoid gated attention convolutional neural network (SiGaAtCNN) Stacked radiomic features; 10-F CV, LR, SVM, MDNNMD	significant enhancements 5.1 in sensitivity values; AUC 95.0, ACC 91.2, ,PRE 84.1, SEN 79.8; **Prognosis**
Yamashita et al. (101); 2 Centers; 190	3 modalities; **Breast Cancer**; Microsatellite instability prediction; DL Pathomics, pathologists	–; Depth features; unclear	ResNet18 Model; 4-F CV, Confusion matrix	AUC 77.9, 95% CI [0.779-0.838]; sensitivity-weighted operating: AUC 93.7, 95% CI: [0.93-0.962]; **Prognosis**
Klein et al. (102); 2 Centers; 594	WSI; Oropharyngeal squamous cell carcinomas **(OPSCC)**; identifies patients with a favorable prognosis; DL Pathomics	–; Depth features; unclear	MCOX-PHR; DenseNet architecture classified, CHST, MWUT, Kaplan Meier survival curve	The Human papillomavirus association could serve as a single biomarker and potentially identify OPSCC patients with a favorable prognosis; **Prognosis**
Wang et al. (103); 2 Hospital; 29701: 20336+9365	WSI; **Gastric cancer**; Survival outcomes, Lymph nodes with metastases (MLN); DL	Stage, Grade, Age, Size, Type, Blood, Location, Sex; Depth features; Tumor-area-to-MLN-area ratio (T/MLN)	U-Net, ResNet-5, T/MLN; MCOX-PHR, Inception V4, ResNet-101, Kaplan–Meier analysis	The calculated T/MLN as a prognostic marker for interpretable; **Prognosis**

Continued on next page

Table S3 — Continued from previous page

Study;Dataset; Total:Train+Test	Modality;Patient; Predictive;Method	Features (Clinical;Radiomic;Predictive)	Model (Construct;Statistics)	Predictive performance(%); Application Type
Shi et al. (104); 1 Hospital, TCGA; 2771: *	WSI; **Hepatocellular carcinoma**; Survival outcomes, Risk stratification (5), Tissue regional; Pathomics + DL	14; Depth features; Sinusoidal capillarisation, Prominent nucleoli and karyotheca, nucleus/cytoplasm ratio, infiltrating inflammatory cells	ResNet-18 + CRF, ResNet-50 + MCOX-PHR; Select (SPC, DCC), Heatmaps (t-SNE and K-means), LRT	Tumour risk score (TRS): 5 risk groups, associated with tumor immune infiltration and gene mutations; **Prognosis**
Wulczyn et al. (105); TCGA; 15104: 12095+3009	WSI; **10 Cancer**; Survival outcomes; Pathomics + DL	Stage, Age, Sex; Depth features; *	Deep learning system (DLS), Baseline; Kaplan-Meier, Cox_PHM, Heatmaps, C-index, Delta	DLS and survival: Significant correlation; 5-years (DLS): increase d 6.4% AUC; **Prognosis**
Chen et al. (106); TCGA; 2756 (images), 677 (Genomic): *	WSI; **Glioma**, clear cell renal cell carcinoma (**CRCC**); Survival outcomes; Pathomics + Genomics	677 genomic features; GCN: 8 contour, 4 texture, CNN: *, SNN: *; 15	CNN, Graph Convolutional (GNN) and Self-Normalizing (SNN) Networks; 15-F CV, Cox loss, Cross entropy	CNN + GCN + SNN: C-Index Glioma 82.6, CCRCC 72.0; **Prognosis**
**Multi-modality images**				
Guo et al. (107); 1 Center; 97: 70%+30%	MRI(T2-weighted images (T2WI), Diffusion-weighted images (DWI)), ceCT; **Rectal cancer**; Perineural invasion; Radiomics	10 (CA19-9, T and N staging, etc.); 396 (42 first-order, 345 high order, 9 Morphological); 5 T2WI, 1 DWI, 12 ceCT	Univariable and multivariable LR analysis; mRMR, LASSO, MWUT, DLT, Radiomics scores, Decision curve analysis, Nomogram	CT and T2WI scores: AUC 90.6 95% CI [0.761-1.000]; The multi-modality radiomics nomogram had a higher clinical net benefit. **Diagnosis**
Khan et al. (108); 1 Center; T1, T1CE, T2, Flair: *	MRI; **Brain Tumor**; Benign or malignant; DL Radiomics	–; Depth: VGG16, VGG19; Fusion: partial least square	VGG16/VGG19 + ELM; Model (SVM, NAB, Softmax, Ensemble tree), 10-F CV, K-Means clustering, Texture histogram equalization	ACC: BraTs2015 98.16, BraTs2017 97.26, BraTs2018 93.40; **Diagnosis**
Wu et al. (109); 1 Center; 1682: *	MRI, CT; **Lung, breast and brain malignancies**; tumor classification; DL Radiomics	stage, tumor volume, etc; 313 (tumor morphology and spatial heterogeneity); 10 (313 to 10)	MCOX-PHR; U-Net45, Consensus clustering, 10,000-times bootstraps, Kaplan Meier survival curve, Benjamini–Hochberg method	Automatic tumor segmentation and reproducible subtype identification; **Diagnosis**
Zhao et al. (110); 1 Center; 256: *	Non-contrast magnetic resonance; **Liver tumor** (hemangioma and hepatocellular carcinoma; Segmentation and detection; DL Radiomics	–; the multi-size edge dissimilarity maps by edge dissimilarity feature pyramid module; *	United adversarial learning framework; 5-F CV, compared: (U-net, Rg-GAN) and (Faster R-CNN and Tripartite-GAN)	segmentation: DC 83.63, ACC 97.75, Intersection-over-union 81.30; classification: TPR: 92.13, TNR: 93.75, ACC 92.4; **Diagnosis**
Alvarez-Jimenez et al. (111); CPTAC, TCGA; 171: 117+59, 146: CV	WSI, CT; **NSCLC**; Subtypes: ADC, SCC; Radiomics + Pathomics	7+9; Haralick texture features + statistics: WSI 360, CT 120; Cross-scale: Cell density, CT intensity	Linear SVM; Select (SPRC, FDRC, WLCR), Heatmap, 10-F CV	AUC: Pathomic (CPTAC 72, TCGA 77), Radiomic 72 (TCGA), Fusion: 78 (TCGA); **Diagnosis**
Giardina et al. (112); 1 Center; 30: *	; OCT, MPM and LSRM; **Pituitary adenomas**; Discrimination of pituitary gland and adenomas; Radiomics	–; 98 (healthy gland tissue and adenoma); 6 (OCT_GLCM, TPEF_GLCM, OCT_GLSZM, TPET_GLSZM, TPEF_GLSZM, MPM_GLSZM)	Radiomic analysis (OCT, MPM, and LSRM); P-values <0.05	First-level binary classification: ACC 88, Second-level multi-class classification: ACC 99; **Diagnosis**
Shiri et al. (7); TCGA; 150: 82+68	CT, Contrast-enhanced CT, PET; **NSCLC**; EGFR, KRAS mutations; Radiomics + Metabolomics	*; 109: First-order, Shape, GLCM, GLRLM, GLSZM, NGTDM, GLDM; 90 EGFR, 14 KRAS	SVM, KNN, DT, QDA, NAB, EML; Select (TET, SPC), 10-F CV, Z-scores, Heatmaps	AUC multivariate: EGFR 82, KRAS 83; **Diagnosis**
Zhou et al. (113); 1 Center; 116: *	Structural MRI (sMRI), Diffusion Tensor Images (DTI) , resting-state fMRI (rsfMRI); **Adolescent**; Diagnosis of early ADHD; Radiomics	–; Boruta based features; 4 T1/2WI, 6 DTI, 10 rsfMRI	Multimodal machine learning framework; SVM, 10-F CV, Heatmaps	AUC 69.8, ACC 64.3, SEN 60.9, SPE 67.6, F1-score 62.6; Significant improvement over the early feature fusion and unimodal features; **Diagnosis**
Calisto et al. (114); 1 Hospital; 289: *	MammoGraphy, US, MRI, text, and annotations; **Breast cancer**; Breast image classification; Radiomics	*; Radiomic features; *	BreastScreening; Heatmaps, BI-RADS score (by the DenseNet), ANOVA factorial analysis	A significant reduction of cognitive workload and improvement in diagnosis execution; **Diagnosis**
Joo et al. (115); 1 Center; 536: 80%+20%	MRI, 3D dynamic contrast-enhanced sequence; **Breast cancer**; pathologic complete response (pCR) to neoadjuvant chemotherapy (NAC); DL Radiomics	Clinicopathologic characteristics; Depth features (by the 3D-ResNet); 6 dimensions	7 DL models; Nearest neighbor interpolation, Histogram-matching algorithm, CHST, FET	Clinical model: ACC 82.7, P <0.05; Multimodal fusion model: AUC 88.8, ACC 85.0, SEN 66.7, SPE 93.2, PPV 81.4, NPV 86.3 ; **Prognosis**
Yang et al. (116); 3 Centers; 1633 CT, 3414 blood samples: *	CT, laboratory, and baseline clinical datas; **Advanced NSCLC**; Clinical outcomes; DL Radiomics	Medical history; Depth features; *	Simple Temporal Attention (SimTA) model; Multiple layer perceptron (MLP), MCOX-PHR, 5-F CV, Kaplan-Meier method, LRT	AUC 80, 95% CI [74-86]; Provides a promising methodology for predicting response; **Prognosis**

Continued on next page

Table S3 — Continued from previous page

Study;Dataset; Total:Train+Test	Modality;Patient; Predictive;Method	Features (Clinical;Radiomic;Predictive)	Model (Construct;Statistics)	Predictive performance(%); Application Type
Lv et al. (10); TCGA; 296: *	PET, CT; **Head and neck cancer**; Survival outcomes; Radiomics + Metabolomics	8; 127: Shape, intensity, Histogram, GLCM, GLRLM, GLSZM, NGTDM, GLGLM, NGLDM, GLDZM; Top 10	MCOX-PHR; Select (SPC), 3-F CV, C-index, AIC, Kaplan-Meier curves	fusion strategy captures more useful features; **Prognosis**
Amini et al. (6); TCIA; 182: *	PET, CT; **NSCLC**; Improved prognosis; Radiomics	11; 225 (first-order features, 136 textural features: GLCM, GLRLM, GLSZM, GLDZM, NGTDM, NGLDM), 10 moment invariant features; Top 10	Multi-level multi-modality radiomics framework; MCOX-PHR, SPRC, HCI, Kaplan–Meier curves, LRT, Feature-maps	The clinical model outperformed the single-modality but was beaten by the image-level fusion strategy; **Prognosis**
Yan et al. (117); 1 Center; 2313: 80%+20%	MRI; **Glioblastoma**; Glioblastoma progression phenotypes preoperatively; Radiomics, DL Radiomics	10; 841 (92 features: first-order, second-order, and filtered wavelet), 9 MR sequences); 153	Machine learning model, DL model; ML (SVM, KNN, DT), DL (VGG16, ResNet50), TET	ML model: overall AUC 81 (77.5-82.5), AUC 83-89; ResNet50: overall AUC; VGG16: overall AUC 96.1; **Prognosis**
Mariscotti et al. (118); 1 Center; 1574: *	2D mammography, DBT, MRI, US; **Breast cancer**; Surgical outcomes; Radiomics + Ultrasomics	Age, Dimension, Density, Multifocality, Size, Histology; –; Tumor size, Multifocality, Histology	LR; Select (SWT, MWUT, XET, FET), Uni- and multivariate analysis	Larger and multiple lesions significantly associated with mastectomy; **Prognosis**
**3D radiotherapy dose distribution (“images“)**				
Rossi et al. (119); Mutiple centers; 351:*	Radiotherapy; **Prostate cancer**; Gastrointestinal (GI) and genitourinary (GU) toxicity; Dosiomics	DVH and non-treatment related (NTR) parameters; 42 texture analysis (TA) featrues; *	Normal Tissue Complication Probability (NTCP) models; NTCP: NTR, NTR + DVH, NTR + TA, NTR + DVH + TA	GI (AUC): NTR (58), NTR + DVH (68) or NTR + TA (73); GU (AUC): NTR (64), NTR + TA (66), NTR + DVH (no improvement); **Toxicity**
Gabrys et al. (120); 1 hospital; 153:*	Intensity modulated radiotherapy, Tomotherapy; **Head and neck cancer**; Xerostomia; Dosiomics	6; Shape, Dose shape, Demographic; Dosiomic, radiomic, and demographic features	Logit and probit NTCP models; Univariate (Z-score, MWUT, LR model), Multivariate (6 feature selection algorithms, 7 classification algorithms); 10 cleaning/class balancing algorithms	Xerostomia (AUC); Late: anterior–posterior (AP): 72, right–left (RL): 68, Long-term: AP: >72, RF: >78, Parotid volumes: >85; **Toxicity**
Liang et al. (121); 1 hospital; 70:*	Volumetric modulated arc therapy; **NSCLC**; Radiation pneumonitis (RP); dosiomics	NTCP factors; 129: GLCM, GLRLM; NTCP, dosiomics, and dosimetric featrures	Univariate and multivariate LR; Dosimetric model (DCM), NTCP model (NTCPM), Dosiomics model (DSM)	Univariate LR (AUC: DCM, NTCPM, DSM): 66.5, 71.0, 70.9; Multivariate LR: 67.6, 74.4, 78.2; **Toxicity**
Adachi et al. (122); Mutiple centers; 247: 70% + 30%	Stereotactic body radiation therapy; **Early-stage NSCLC**; Radiation pneumonitis (RP); dosiomics	10 dose–volume indices (DVIs); 6808: shape, first order, texture features; 10 DVIs, selected dosiomic features, hybrid features	LightGBM; DVI, dosiomics, and hybrid (DVIs + dosiomics) models, z-scores, 5-F CV	(ROC–AUC, PR–AUC): DVI (66.0 ± 5.4, 27.2 ± 5.2), dosiomics (83.7 ± 5.4, 51.0 ± 11.5), hybrid (84.6 ± 4.9, 53.1 ± 11.6); **Toxicity**
Lee et al. (123); 1 hospital; 388:*	Intensity modulated radiotherapy; **Lung cancer**; Weight loss (WL); dosiomics	Clinical parameter (CP): 10, DVH: 74; 355: CPs, DVHs, radiomics, and dosiomics featrues; Radiomics and dosiomics (R&D) featrues	Ensemble; 7 Inputs: CP, DVH, R&D, DVH + CP, R&D + CP, R&D + DVH, R&D + DVH + CP; Univariate LR, Multivariate analysis (SVM, DNN, Ensemble)	Ensemble classifier: AUC (95% CI) 71.0 (63.7, 78.2); **Toxicity**
Liang et al. (124); 2 hospitals; 70:*	Volumetric modulated arc therapy; **NSCLC**; Radiation pneumonitis (RP); DL dosiomics	Dosimetric and NTCP featrues; *; Dosimetric, NTCP, and dosiomics factors	Multivariate LR; Convolution 3D (C3D) neural network)	C3D model (AUC): 84.2; 3 LR models (AUC): 67.6, 74.4, and 78.2; **Toxicity**
Wu et al. (125); 4 institutions; 237:*	Intensity modulated radiotherapy; **Head and neck cancer**; Locoregional recurrence; Dosiomics	Age, Sex,T-stage, N-stage, TNM-stage, Tumor site, Treatment; Radiomic and dosiomic features; *	MCOX-PHR (RadModel, RadDosModel); CI, Kaplan–Meier curves, CPD model (dose signatures), CLD model (clinical parameters), CPD + CLD model, Dosiomics, LASSO	Dosiomics: CI 66, radiomics: CI 59; Dosiomics: successfully classified the patients into high- and low-risk groups; **Prognosis**
Murakami et al. (126); 1 hospital; 489: 342 + 147	Intensity modulated radiotherapy; **Prostate cancer**; Biochemical failure; Dosiomics	8 clinical variables, 35 dosimetric parameters (DVHs); 2475 dosiomic featrues, 1038 radiomic featrues; 3 clinical variables, 2 dosiomic features	Dosiomics model (multivariate LR); Dosiomics and radiomics models; Kaplan-Meier curves; 5-F CV; MWUT; AIC; SPC	CI: dosiomics 66, radiomics 59; Classified the patients into high- and low-risk groups; **Prognosis**
Buizza et al. (127); 1 center; 57: 80% + 20%	Carbon-ion radiotherapy; **Skull-base chordomas (SBC)**; Local control; Dosiomics	7 clinical variables, 14 shape, 18 first-order, and 75 textural features; MRI and CT-based radiomic, and dosiomic features;	Dosiomics models (SVM, MCOX-PHR); 2 survival models, 5-F CV; Single modality; Combined Modalities (imaging, dose and clinical information); PCA; Kaplan-Meier survival curves	Dosiomic and combined models could consistently stratify patients in high- and low-risk groups, C-index: 80/24, 79/26; **Prognosis**
Hirashima et al. (128); 1 hospital; 888:*	Volumetric modulated arc therapy; **Brain tumor**; Gamma passing rate (GPR) value; Dosiomics	24 plan complexity features; 851 dosiomic featrues; plan complexity features, 3D dosiomic feature; Combination of both	XGBoost models (plan, dosiomics, hybrid); Mean absolute error (MAE), Correlation coefficient (CC), SPRC, ANOVA test	MAE, CC: plan (4.6, 58), dosiomics (4.3, 0.61), hybrid (4.2, 63); **Prognosis**

Continued on next page

Table S3 — Continued from previous page

Study;Dataset; Total:Train+Test	Modality;Patient; Predictive;Method	Features (Clinical;Radiomic;Predictive)	Model (Construct;Statistics)	Predictive performance(%); Application Type
Placidi et al. (129); 8 centers; 30 different dose distributions	Radiotherapy; **Tumor patierns**; the features' stability and capability; Dosiomics	–; 38160 dosiomic featrues, 17 intensity-based statistics, 100 radiomic features, Treatment planning system (TPS); –	Various dose distributions, different dose calculation algorithms, 2 different resolutions of the dose grid; Coefficient of variation (CV), reproducibility/stability (CV <3), sensitivity (CV >1)	Dosiomic characteristics are sensitive to changes in dose calculation parameters and consistent reporting of TPS, dose calculations.**Reproducibility, stability and sensitivity**

ACC=Accuracy;SEN=Sensitivity;SPE=Specificity;PRE=Precision;PPV=Positive predictive value;NPV=Negative predictive value;Dice coefficient=DC;Channel-wise Attention = CA;DL=Deep Learning;F CV=Fold cross-validation;GTV=Gross tumor volume;LOOCV=Leave one out CV;RFs=Radiomic features;Cox-CNN=Convolutional Neural Network–based Cox proportional hazards regression;Contrast-enhanced CT images=ceCT;Non-contrast-enhanced=non-ceCT;Laplacian of Gaussian=LoG;Dose-volume-histogram=DVH;Area under the receiver operating characteristic curve=AUROC;Area under the precision-recall curve=AUPRC;*=Unclear;Other=See Table S2.

REFERENCES

[1]Verma, A. A., Pasricha, S. V., Jung, H. Y., Kushnir, V., Mak, D. Y. F., Koppula, R., et al. (2021). Assessing the quality of clinical and administrative data extracted from hospitals: the general medicine inpatient initiative (gemini) experience. *Journal of the American Medical Informatics Association* 28, 578–587. doi:10.1093/jamia/ocaa225

[2]Nazari, M., Shiri, I., and Zaidi, H. (2021). Radiomics-based machine learning model to predict risk of death within 5-years in clear cell renal cell carcinoma patients. *Computers in Biology and Medicine* 129. doi:ARTN10413510.1016/j.compbiomed.2020.104135

[3]van Timmeren, J. E., Cester, D., Tanadini-Lang, S., Alkadhi, H., and Baessler, B. (2020). Radiomics in medical imaging-"how-to" guide and critical reflection. *Insights into Imaging* 11. doi:ARTN9110.1186/s13244-020-00887-2

[4]Mazzei, M. A., Di Giacomo, L., Bagnacci, G., Nardone, V., Gentili, F., Lucii, G., et al. (2021). Delta-radiomics and response to neoadjuvant treatment in locally advanced gastric cancer-a multicenter study of gircg (italian research group for gastric cancer). *Quantitative Imaging in Medicine and Surgery* 11, 2376–2387. doi:10.21037/qims-20-683

[5]Li, M. J., Chen, T., Zhao, W. L., Wei, C. G., Li, X. B., Duan, S. F., et al. (2020). Radiomics prediction model for the improved diagnosis o clinically significant prostate cancer on biparametric mri. *Quantitative Imaging in Medicine and Surgery* 10, 368–+. doi:10.21037/qims.2019.12.06

[6]Amini, M., Nazari, M., Shiri, I., Hajianfar, G., Deevband, M. R., Abdollahi, H., et al. (2021). Multi-level multi-modality (pet and ct) fusion radiomics: prognostic modeling for non-small cell lung carcinoma. *Physics in Medicine and Biology* 66. doi:ARTN20501710.1088/1361-6560/ac287d

[7]Shiri, I., Maleki, H., Hajianfar, G., Abdollahi, H., Ashrafinia, S., Hatt, M., et al. (2020). Next-generation radiogenomics sequencing for prediction of egfr and kras mutation status in nsclc patients using multimodal imaging and machine learning algorithms. *Molecular Imaging and Biology* 22, 1132–1148. doi:10.1007/s11307-020-01487-8

[8]Shiri, I., Sorouri, M., Geramifar, P., Nazari, M., Abdollahi, M., Salimi, Y., et al. (2021). Machine learning-based prognostic modeling using clinical data and quantitative radiomic features from chest ct images in covid-19 patients. *Computers in Biology and Medicine* 132. doi:ARTN10430410.1016/j.compbiomed.2021.104304

[9]Mashayekhi, R., Parekh, V. S., Faghih, M., Singh, V. K., Jacobs, M. A., and Zaheer, A. (2020). Radiomic features of the pancreas on ct imaging accurately differentiate functional abdominal pain, recurrent acute pancreatitis, and chronic pancreatitis. *European Journal of Radiology* 123. doi:ARTN10877810.1016/j.ejrad.2019.108778

[10]Lv, W. B., Ashrafinia, S., Ma, J. H., Lu, L. J., and Rahmim, A. (2020). Multi-level multi-modality fusion radiomics: Application to pet and ct imaging for prognostication of head and neck cancer. *Ieee Journal of Biomedical and Health Informatics* 24, 2268–2277. doi:10.1109/Jbhi.2019.2956354

[11]Yang, X., Yu, L. Q., Li, S. L., Wen, H. X., Luo, D. D., Bian, C., et al. (2019). Towards automated semantic segmentation in prenatal volumetric ultrasound. *Ieee Transactions on Medical Imaging* 38, 180–193. doi:10.1109/Tmi.2018.2858779

[12]Feng, D., Haase-Schutz, C., Rosenbaum, L., Hertlein, H., Glaser, C., Timm, F., et al. (2021). Deep multi-modal object detection and semantic segmentation for autonomous driving: Datasets, methods, and challenges. *Ieee Transactions on Intelligent Transportation Systems* 22, 1341–1360. doi:10.1109/Tits.2020.2972974

[13]Kattenborn, T., Leitloff, J., Schiefer, F., and Hinz, S. (2021). Review on convolutional neural networks (cnn) in vegetation remote sensing. *Isprs Journal of Photogrammetry and Remote Sensing* 173, 24–49. doi:10.1016/j.isprsjprs.2020.12.010

[14]Ji, Y. Z., Zhang, H. J., Zhang, Z., and Liu, M. (2021). Cnn-based encoder-decoder networks for salient object detection: A comprehensive review and recent advances. *Information Sciences* 546, 835–857. doi:10.1016/j.ins.2020.09.003

[15]Sun, J. D., Peng, Y. J., Guo, Y. F., and Li, D. P. (2021). Segmentation of the multimodal brain tumor image used the multi-pathway architecture method based on 3d fcn. *Neurocomputing* 423, 34–45. doi:10.1016/j.neucom.2020.10.031

[16]Aslan, M. F., Unlersen, M. F., Sabanci, K., and Durdu, A. (2021). Cnn-based transfer learning-bilstm network: A novel approach for covid-19 infection detection. *Applied Soft Computing* 98. doi:ARTN10691210.1016/j.asoc.2020.106912

[17]Samuel, P. M. and Veeramalai, T. (2021). Vssc net: Vessel specific skip chain convolutional network for blood vessel segmentation. *Computer Methods and Programs in Biomedicine* 198. doi:ARTN10576910.1016/j.cmpb.2020.105769

[18]Mohammed, M. A., Abdulkareem, K. H., Garcia-Zapirain, B., Mostafa, S. A., Maashi, M. S., Al-Waisy, A. S., et al. (2021). A comprehensive investigation of machine learning feature extraction and classification methods for automated diagnosis of covid-19 based on x-ray images. *Cmc-Computers Materials & Continua* 66, 3289–3310. doi:10.32604/cmc.2021.012874

[19]Li, X., Jiang, Y. C., Li, M. L., and Yin, S. (2021). Lightweight attention convolutional neural network for retinal vessel image segmentation. *Ieee Transactions on Industrial Informatics* 17, 1958–1967. doi:10.1109/Tii.2020.2993842

[20]Jin, J. B., Zhu, H. Y., Zhang, J. D., Ai, Y., Zhang, J., Teng, Y. Y., et al. (2021). Multiple u-net-based automatic segmentations and radiomics feature stability on ultrasound images for patients with ovarian cancer. *Frontiers in Oncology* 10. doi:ARTN61420110.3389/fonc.2020.614201

[21]Zhao, C., Xu, Y., He, Z., Tang, J. S., Zhang, Y. J., Han, J. G., et al. (2021). Lung segmentation and automatic detection of covid-19 using radiomic features from chest ct images. *Pattern Recognition* 119. doi:ARTN10807110.1016/j.patcog.2021.108071

[22]Junger, S. T., Hoyer, U. C. I., Schaufler, D., Laukamp, K. R., Goertz, L., Thiele, F., et al. (2021). Fully automated mr detection and segmentation of brain metastases in non-small cell lung cancer using deep learning. *Journal of Magnetic Resonance Imaging* 54, 1608–1622. doi:10.1002/jmri.27741

[23]Looney, P., Yin, Y., Collins, S. L., Nicolaides, K. H., Plasencia, W., Molloholli, M., et al. (2021). Fully automated 3-d ultrasound segmentation of the placenta, amniotic fluid, and fetus for early pregnancy assessment. *Ieee Transactions on Ultrasonics Ferroelectrics and Frequency Control* 68, 2038–2047. doi:10.1109/Tuffc.2021.3052143

[24]Fiorentino, M. C., Moccia, S., Capparuccini, M., Giamberini, S., and Frontoni, E. (2021). A regression framework to head-circumference delineation from us fetal images. *Computer Methods and Programs in Biomedicine* 198. doi:ARTN10577110.1016/j.cmpb.2020.105771

[25]Islam, Z., Abdel-Aty, M., Cai, Q., and Yuan, J. H. (2021). Crash data augmentation using variational autoencoder. *Accident Analysis and Prevention* 151. doi:ARTN10595010.1016/j.aap.2020.105950

[26]Huang, Z. A., Zhu, Z. X., Yau, C. H., and Tan, K. C. (2021). Identifying autism spectrum disorder from resting-state fmri using deep belief network. *Ieee Transactions on Neural Networks and Learning Systems* 32, 2847–2861. doi:10.1109/Tnnls.2020.3007943

[27]Zhang, Z., Chen, P., Shi, X., and Yang, L. (2021). Text-guided neural network training for image recognition in natural scenes and medicine. *IEEE Trans Pattern Anal Mach Intell* 43, 1733–1745. doi:10.1109/TPAMI.2019.2955476

[28]Farahmand, S., Fernandez, A. I., Ahmed, F. S., Rimm, D. L., Chuang, J. H., Reisenbichler, E., et al. (2021). Deep learning trained on hematoxylin and eosin tumor region of interest predicts her2 status and trastuzumab treatment response in her2+breast cancer. *Modern Pathology* doi:10.1038/s41379-021-00911-w

[29]Pang, T., Wong, J. H. D., Ng, W. L., and Chan, C. S. (2021). Semi-supervised gan-based radiomics model for data augmentation in breast ultrasound mass classification. *Comput Methods Programs Biomed* 203, 106018. doi:10.1016/j.cmpb.2021.106018

[30]Chaunzwa, T. L., Hosny, A., Xu, Y. W., Shafer, A., Diao, N., Lanuti, M., et al. (2021). Deep learning classification of lung cancer histology using ct images. *Scientific Reports* 11. doi:10.1038/s41598-021-84630-x

[31]Khosravi, P., Kazemi, E., Imielinski, M., Elemento, O., and Hajirasouliha, I. (2018). Deep convolutional neural networks enable discrimination of heterogeneous digital pathology images. *Ebiomedicine* 27, 317–328. doi:10.1016/j.ebiom.2017.12.026

[32]Zhou, H., Jin, Y. H., Dai, L., Zhang, M. W., Qiu, Y. Q., Wang, K., et al. (2020). Differential diagnosis of benign and malignant thyroid nodules using deep learning radiomics of thyroid ultrasound images. *European Journal of Radiology* 127. doi:ARTN10899210.1016/j.ejrad.2020.108992

[33]Fu, Y. B., Lei, Y., Wang, T. H., Patel, P., Jani, A. B., Mao, H., et al. (2021). Biomechanically constrained non-rigid mr-trus prostate registration using deep learning based 3d point cloud matching. *Medical Image Analysis* 67. doi:ARTN10184510.1016/j.media.2020.101845

[34]Zhang, Y., Wu, J., Liu, Y. L., Chen, Y. F., Chen, W., Wu, E. X., et al. (2021). A deep learning framework for pancreas segmentation with multi-atlas registration and 3d level-set. *Medical Image Analysis* 68. doi:ARTN10188410.1016/j.media.2020.101884

[35]Gehlot, S., Gupta, A., and Gupta, R. (2021). A cnn-based unified framework utilizing projection loss in unison with label noise handling for multiple myeloma cancer diagnosis. *Medical Image Analysis* 72. doi:ARTN10209910.1016/j.media.2021.102099

[36]Luo, Y. M., Majoe, S., Kui, J., Qi, H. K., Pushparajah, K., and Rhode, K. (2021). Ultra-dense denoising network: Application to cardiac catheter-based

x-ray procedures. *Ieee Transactions on Biomedical Engineering* 68, 2626–2636. doi:10.1109/Tbme.2020.3041571

[37]Dastider, A. G., Sadik, F., and Fattah, S. A. (2021). An integrated autoencoder-based hybrid cnn-lstm model for covid-19 severity prediction from lung ultrasound. *Computers in Biology and Medicine* 132. doi:ARTN10429610.1016/j.compbiomed.2021.104296

[38]Hou, L., Nguyen, V., Kanevsky, A. B., Samaras, D., Kurc, T. M., Zhao, T. H., et al. (2019). Sparse autoencoder for unsupervised nucleus detection and representation in histopathology images. *Pattern Recognition* 86, 188–200. doi:10.1016/j.patcog.2018.09.007

[39]Yun, J., Park, J. E., Lee, H., Ham, S., Kim, N., and Kim, H. S. (2019). Radiomic features and multilayer perceptron network classifier: a robust mri classification strategy for distinguishing glioblastoma from primary central nervous system lymphoma. *Scientific Reports* 9. doi:ARTN574610.1038/s41598-019-42276-w

[40]Ho, T. Y., Chao, C. H., Chin, S. C., Ng, S. H., Kang, C. J., and Tsang, N. M. (2020). Classifying neck lymph nodes of head and neck squamous cell carcinoma in mri images with radiomic features. *Journal of Digital Imaging* 33, 613–618. doi:10.1007/s10278-019-00309-w

[41]Shao, M. W., Zhang, G. Z., Zuo, W. M., and Meng, D. Y. (2021). Target attack on biomedical image segmentation model based on multi-scale gradients. *Information Sciences* 554, 33–46. doi:10.1016/j.ins.2020.12.013

[42]Han, X. T., Hu, Y. X., Foschini, L., Chinitz, L., Jankelson, L., and Ranganath, R. (2020). Deep learning models for electrocardiograms are susceptible to adversarial attack. *Nature Medicine* 26, 360–+. doi:10.1038/s41591-020-0791-x

[43]Zhang, J. L. and Li, C. (2020). Adversarial examples: Opportunities and challenges. *Ieee Transactions on Neural Networks and Learning Systems* 31, 2578–2593. doi:10.1109/Tnnls.2019.2933524

[44]Singh, S. P., Wang, L. P., Gupta, S., Gulyas, B., and Padmanabhan, P. (2021). Shallow 3d cnn for detecting acute brain hemorrhage from medical imaging sensors. *Ieee Sensors Journal* 21, 14290–14299. doi:10.1109/Jsen.2020.3023471

[45]Meng, L. W., Dong, D., Chen, X., Fang, M. J., Wang, R. P., Li, J., et al. (2021). 2d and 3d ct radiomic features performance comparison in characterization of gastric cancer: A multi-center study. *Ieee Journal of Biomedical and Health Informatics* 25, 755–763. doi:10.1109/Jbhi.2020.3002805

[46]Khorrami, M., Prasanna, P., Gupta, A., Patil, P., Velu, P. D., Thawani, R., et al. (2020). Changes in ct radiomic features associated with lymphocyte distribution predict overall survival and response to immunotherapy in non-small cell lung cancer. *Cancer Immunology Research* 8, 108–119. doi:10.1158/2326-6066.Cir-19-0476

[47]Gao, K., Su, J. P., Jiang, Z. B., Zeng, L. L., Feng, Z. C., Shen, H., et al. (2021). Dual-branch combination network (dcn): Towards accurate diagnosis and lesion segmentation of covid-19 using ct images. *Medical Image Analysis* 67. doi:ARTN10183610.1016/j.media.2020.101836

[48]Liao, W. M., Zou, B. J., Zhao, R. C., Chen, Y. Q., He, Z. Y., and Zhou, M. J. (2020). Clinical interpretable deep learning model for glaucoma diagnosis. *Ieee Journal of Biomedical and Health Informatics* 24, 1405–1412. doi:10.1109/Jbhi.2019.2949075

[49]Pulvermuller, F., Tomasello, R., Henningsen-Schomers, M. R., and Wennekers, T. (2021). Biological constraints on neural network models of cognitive function. *Nature Reviews Neuroscience* 22, 488–502. doi:10.1038/s41583-021-00473-5

[50]Wang, X. S., Peng, Y. F., Lu, L., Lu, Z. Y., and Summers, R. M. (2018). Tienet: Text-image embedding network for common thorax disease classification and reporting in chest x-rays. *2018 Ieee/Cvf Conference on Computer Vision and Pattern Recognition (Cvpr)* , 9049–9058doi:10.1109/Cvpr.2018.00943

[51]Shen, S. W., Han, S. X., Aberle, D. R., Bui, A. A., and Hsu, W. (2019). An interpretable deep hierarchical semantic convolutional neural network for lung nodule malignancy classification. *Expert Systems with Applications* 128, 84–95. doi:10.1016/j.eswa.2019.01.048

[52]Niu, Y. H., Gu, L., Lu, F., Lv, F. F., Wang, Z. J., Sato, I., et al. (2019). Pathological evidence exploration in deep retinal image diagnosis. *Thirty-Third Aaai Conference on Artificial Intelligence / Thirty-First Innovative Applications of Artificial Intelligence Conference / Ninth Aaai Symposium on Educational Advances in Artificial Intelligence* , 1093–1101

[53]Tobia, K., Nielsen, A., and Stremitzer, A. (2021). When does physician use of ai increase liability? *Journal of Nuclear Medicine* 62, 17–21. doi:10.2967/jnumed.120.256032

[54]Liu, T., Yang, L. X., and Lunga, D. (2021). Change detection using deep learning approach with object-based image analysis. *Remote Sensing of Environment* 256. doi:ARTN11230810.1016/j.rse.2021.112308

[55]Li, C., Xu, J. X., Liu, Q. G., Zhou, Y. J., Mou, L. S., Pu, Z. H., et al. (2021). Multi-view mammographic density classification by dilated and attention-guided residual learning. *Ieee-Acm Transactions on Computational Biology and Bioinformatics* 18, 1003–1013. doi:10.1109/Tcbb.2020.2970713

[56]Kolossvary, M., Gerstenblith, G., Bluemke, D. A., Fishman, E. K., Mandler, R. N., Kickler, T. S., et al. (2021). Contribution of risk factors to the development of coronary atherosclerosis as confirmed via coronary ct angiography: A longitudinal radiomics-based study. *Radiology* 299, 97–106. doi:10.1148/radiol.2021203179

[57]Benedetti, G., Mori, M., Panzeri, M. M., Barbera, M., Palumbo, D., Sini, C., et al. (2021). Ct-derived radiomic features to discriminate histologic characteristics of pancreatic neuroendocrine tumors. *Radiologia Medica* 126, 745–760. doi:10.1007/s11547-021-01333-z

[58]Jiang, X. R., Li, J. X., Kan, Y. Y., Yu, T., Chang, S. J., Sha, X. Z., et al. (2021). Mri based radiomics approach with deep learning for prediction of vessel invasion in early-stage cervical cancer. *Ieee-Acm Transactions on Computational Biology and Bioinformatics* 18, 995–1002. doi:10.1109/Tcbb.2019.2963867

[59]Xie, X. J., Liu, S. Y., Chen, J. Y., Zhao, Y., Jiang, J., Wu, L., et al. (2021). Development of unenhanced ct-based imaging signature for bap1 mutation status prediction in malignant pleural mesothelioma: Consideration of 2d and 3d segmentation. *Lung Cancer* 157, 30–39. doi:10.1016/j.lungcan.2021.04.023

[60]Du, Y., Fang, Z., Jiao, J., Xi, G., Zhu, C., Ren, Y., et al. (2021). Application of ultrasound-based radiomics technology in fetal-lung-texture analysis in pregnancies complicated by gestational diabetes and/or pre-eclampsia. *Ultrasound Obstet Gynecol* 57, 804–812. doi:10.1002/uog.22037

[61]Ligero, M., Garcia-Ruiz, A., Viaplana, C., Villacampa, G., Raciti, M. V., Landa, J., et al. (2021). A ct-based radiomics signature is associated with response to immune checkpoint inhibitors in advanced solid tumors. *Radiology* 299, 109–119. doi:10.1148/radiol.2021200928

[62]Rossi, G., Barabino, E., Fedeli, A., Ficarra, G., Coco, S., Russo, A., et al. (2021). Y radiomic detection of egfr mutations in nsclc. *Cancer Research* 81, 724–731. doi:10.1158/0008-5472.Can-20-0999

[63]Agazzi, G. M., Ravanelli, M., Roca, E., Medicina, D., Balzarini, P., Pessina, C., et al. (2021). Ct texture analysis for prediction of egfr mutational status and alk rearrangement in patients with non-small cell lung cancer. *Radiologia Medica* 126, 786–794. doi:10.1007/s11547-020-01323-7

[64]Choi, Y. S., Bae, S., Chang, J. H., Kang, S. G., Kim, S. H., Kim, J., et al. (2021). Fully automated hybrid approach to predict the idh mutation status of gliomas via deep learning and radiomics. *Neuro-Oncology* 23, 304–313. doi:10.1093/neuonc/noaa177

[65]Wu, G. Y., Woodruff, H. C., Shen, J., Refaee, T., Sanduleanu, S., Ibrahim, A., et al. (2020). Diagnosis of invasive lung adenocarcinoma based on chest ct radiomic features of part-solid pulmonary nodules: A multicenter study. *Radiology* 297, 451–458. doi:10.1148/radiol.2020192431

[66]Liu, Q. F., Sun, D. Z., Li, N., Kim, J. M., Feng, D. G., Huang, G., et al. (2020). Predicting egfr mutation subtypes in lung adenocarcinoma using f-18-fdg pet/ct radiomic features. *Translational Lung Cancer Research* 9, 549–+. doi:10.21037/tlcr.2020.04.17

[67]Zhang, J. Y., Zhao, X. M., Zhao, Y., Zhang, J. M., Zhang, Z. Q., Wang, J. F., et al. (2020). Value of pre-therapy f-18-fdg pet/ct radiomics in predicting egfr mutation status in patients with non-small cell lung cancer. *European Journal of Nuclear Medicine and Molecular Imaging* 47, 1137–1146. doi:10.1007/s00259-019-04592-1

[68]Mu, W., Jiang, L., Zhang, J. Y., Shi, Y., Gray, J. E., Tunali, I., et al. (2020). Non-invasive decision support for nsclc treatment using pet/ct radiomics. *Nature Communications* 11. doi:ARTN522810.1038/s41467-020-19116-x

[69]Haubold, J., Demircioglu, A., Gratz, M., Glas, M., Wrede, K., Sure, U., et al. (2020). Non-invasive tumor decoding and phenotyping of cerebral gliomas utilizing multiparametric f-18-fet pet-mri and mr fingerprinting. *European Journal of Nuclear Medicine and Molecular Imaging* 47, 1435–1445. doi:10.1007/s00259-019-04602-2

[70]Jin, X. C., Ai, Y., Zhang, J., Zhu, H. Y., Jin, J. B., Teng, Y. Y., et al. (2020). Noninvasive prediction of lymph node status for patients with early-stage cervical cancer based on radiomics features from ultrasound images. *European Radiology* 30, 4117–4124. doi:10.1007/s00330-020-06692-1

[71]Peng, Y. T., Lin, P., Wu, L. Y., Wan, D., Zhao, Y. J., Liang, L., et al. (2020). Ultrasound-based radiomics analysis for preoperatively predicting different histopathological subtypes of primary liver cancer. *Frontiers in Oncology* 10. doi:ARTN164610.3389/fonc.2020.01646

[72]Dohan, A., Gallix, B., Guiu, B., Le Malicot, K., Reinhold, C., Soyer, P., et al. (2020). Early evaluation using a radiomic signature of unresectable hepatic metastases to predict outcome in patients with colorectal cancer treated with folfiri and bevacizumab. *Gut* 69, 531–539. doi:10.1136/gutjnl-2018-316407

[73]Chen, M. Y., Cao, J. S., Hu, J. H., Topatana, W., Li, S. J., Juengpanich, S., et al. (2021). Clinical-radiomic analysis for pretreatment prediction of objective

response to first transarterial chemoembolization in hepatocellular carcinoma. *Liver Cancer* 10, 38–51. doi:10.1159/000512028

[74]Dissaux, G., Visvikis, D., Da-ano, R., Pradier, O., Chajon, E., Barillot, I., et al. (2020). Pretreatment f-18-fdg pet/ct radiomics predict local recurrence in patients treated with stereotactic body radiotherapy for early-stage non-small cell lung cancer: A multicentric study. *Journal of Nuclear Medicine* 61, 814–820. doi:10.2967/jnumed.119.228106

[75]Fatima, K., Dasgupta, A., DiCenzo, D., Kolios, C., Quiaoit, K., Saifuddin, M., et al. (2021). Ultrasound delta-radiomics during radiotherapy to predict recurrence in patients with head and neck squamous cell carcinoma. *Clinical and Translational Radiation Oncology* 28, 62–70. doi:10.1016/j.ctro.2021.03.002

[76]Xiong, Q. Q., Zhou, X. Z., Liu, Z. Y., Lei, C. Q., Yang, C. Q., Yang, M., et al. (2020). Multiparametric mri-based radiomics analysis for prediction of breast cancers insensitive to neoadjuvant chemotherapy. *Clinical & Translational Oncology* 22, 50–59. doi:10.1007/s12094-019-02109-8

[77]DiCenzo, D., Quiaoit, K., Fatima, K., Bhardwaj, D., Sannachi, L., Gangeh, M., et al. (2020). Quantitative ultrasound radiomics in predicting response to neoadjuvant chemotherapy in patients with locally advanced breast cancer: Results from multi-institutional study. *Cancer Medicine* 9, 5798–5806. doi:10.1002/cam4.3255

[78]Jiang, M., Li, C. L., Luo, X. M., Chuan, Z. R., Lv, W. Z., Li, X., et al. (2021). Ultrasound-based deep learning radiomics in the assessment of pathological complete response to neoadjuvant chemotherapy in locally advanced breast cancer. *European Journal of Cancer* 147, 95–105. doi:10.1016/j.ejca.2021.01.028

[79]Quiaoit, K., DiCenzo, D., Fatima, K., Bhardwaj, D., Sannachi, L., Gangeh, M., et al. (2020). Quantitative ultrasound radiomics for therapy response monitoring in patients with locally advanced breast cancer: Multi-institutional study results. *Plos One* 15. doi:ARTNe023618210.1371/journal.pone.0236182

[80]Hu, Y. H., Xie, C. Y., Yang, H., Ho, J. W. K., Wen, J., Han, L. J., et al. (2021). Computed tomography-based deep-learning prediction of neoadjuvant chemoradiotherapy treatment response in esophageal squamous cell carcinoma. *Radiotherapy and Oncology* 154, 6–13. doi:10.1016/j.radonc.2020.09.014

[81]Haider, S. P., Sharaf, K., Zeevi, T., Baumeister, P., Reichel, C., Forghani, R., et al. (2021). Prediction of post-radiotherapy locoregional progression in hpv-associated oropharyngeal squamous cell carcinoma using machine-learning analysis of baseline pet/ct radiomics. *Translational Oncology* 14. doi:ARTN10090610.1016/j.tranon.2020.100906

[82]Zhao, S. J., Hou, D. H., Zheng, X. M., Song, W., Liu, X. Q., Wang, S. C., et al. (2021). Mri radiomic signature predicts intracranial progression-free survival in patients with brain metastases of alk-positive non-small cell lung cancer. *Translational Lung Cancer Research* 10. doi:10.21037/tlcr-20-361

[83]Ferreira, M., Lovinfosse, P., Hermesse, J., Decuypere, M., Rousseau, C., Lucia, F., et al. (2021). [f-18]fdg pet radiomics to predict disease-free survival in cervical cancer: a multi-scanner/center study with external validation. *European Journal of Nuclear Medicine and Molecular Imaging* 48, 3432–3443. doi:10.1007/s00259-021-05303-5

[84]Krarup, M. M. K., Nygard, L., Vogelius, I. R., Andersen, F. L., Cook, G., Goh, V., et al. (2020). Heterogeneity in tumours: Validating the use of radiomic features on f-18-fdg pet/ct scans of lung cancer patients as a prognostic tool. *Radiotherapy and Oncology* 144, 72–78. doi:10.1016/j.radonc.2019.10.012

[85]Liu, F., Liu, D., Wang, K., Xie, X. H., Su, L. Y., Kuang, M., et al. (2020). Deep learning radiomics based on contrast-enhanced ultrasound might optimize curative treatments for very-early or early-stage hepatocellular carcinoma patients. *Liver Cancer* 9, 397–413. doi:10.1159/000505694

[86]Sharma, S. and Mehra, R. (2020). Conventional machine learning and deep learning approach for multi-classification of breast cancer histopathology images-a comparative insight. *Journal of Digital Imaging* 33, 632–654. doi:10.1007/s10278-019-00307-y

[87]Trivizakis, E., Ioannidis, G. S., Souglakos, I., Karantanas, A. H., Tzardi, M., and Marias, K. (2021). A neural pathomics framework for classifying colorectal cancer histopathology images based on wavelet multi-scale texture analysis. *Scientific Reports* 11. doi:ARTN1554610.1038/s41598-021-94781-6

[88]Kim, C. H., Bhattacharjee, S., Prakash, D., Kang, S., Cho, N. H., Kim, H. C., et al. (2021). Artificial intelligence techniques for prostate cancer detection through dual-channel tissue feature engineering. *Cancers* 13. doi:ARTN152410.3390/cancers13071524

[89]Pei, L. M., Jones, K. A., Shboul, Z. A., Chen, J. Y., and Iftekharuddin, K. M. (2021). Deep neural network analysis of pathology images with integrated molecular data for enhanced glioma classification and grading. *Frontiers in Oncology* 11. doi:ARTN66869410.3389/fonc.2021.668694

[90]Li, Z., Zhang, J. H., Tan, T., Teng, X. C., Sun, X. L., Zhao, H., et al. (2021). Deep learning methods for lung cancer segmentation in whole-slide histopathology images-the acdc@lunghp challenge 2019. *Ieee Journal of Biomedical and Health Informatics* 25, 429–440. doi:10.1109/Jbhi.2020.3039741

[91]Chen, M. Y., Zhang, B., Topatana, W., Cao, J. S., Zhu, H. P., Juengpanich, S., et al. (2020). Classification and mutation prediction based on histopathology h&e images in liver cancer using deep learning. *Npj Precision Oncology* 4. doi:ARTN1410.1038/s41698-020-0120-3

[92]Sun, C. L., Xu, A., Liu, D., Xiong, Z. W., Zhao, F., and Ding, W. P. (2020). Deep learning-based classification of liver cancer histopathology images using only global labels. *Ieee Journal of Biomedical and Health Informatics* 24, 1643–1651. doi:10.1109/Jbhi.2019.2949837

[93]Karimi, D., Nir, G., Fazli, L., Black, P. C., Goldenberg, L., and Salcudean, S. E. (2020). Deep learning-based gleason grading of prostate cancer from histopathology images-role of multiscale decision aggregation and data augmentation. *Ieee Journal of Biomedical and Health Informatics* 24, 1413–1426. doi:10.1109/Jbhi.2019.2944643

[94]Woerl, A. C., Eckstein, M., Geiger, J., Wagner, D. C., Daher, T., Stenzel, P., et al. (2020). Deep learning predicts molecular subtype of muscle-invasive bladder cancer from conventional histopathological slides. *European Urology* 78, 256–264. doi:10.1016/j.eururo.2020.04.023

[95]Wang, X., Chen, H., Gan, C. X., Lin, H. J., Dou, Q., Tsougenis, E., et al. (2020). Weakly supervised deep learning for whole slide lung cancer image analysis. *Ieee Transactions on Cybernetics* 50, 3950–3962. doi:10.1109/Tcyb.2019.2935141

[96]McGarry, S. D., Bukowy, J. D., Iczkowski, K. A., Lowman, A. K., Brehler, M., Bobholz, S., et al. (2020). Radio-pathomic mapping model generated using annotations from five pathologists reliably distinguishes high-grade prostate cancer. *Journal of Medical Imaging* 7. doi:Artn05450110.1117/1.Jmi.7.5.054501

[97]Hu, J., Cui, C. L., Yang, W. X., Huang, L. H., Yu, R. S., Liu, S. Y., et al. (2021). Using deep learning to predict anti-pd-1 response in melanoma and lung cancer patients from histopathology images. *Translational Oncology* 14. doi:ARTN10092110.1016/j.tranon.2020.100921

[98]Qu, H., Zhou, M., Yan, Z. N., Wang, H., Rustgi, V. K., Zhang, S. T., et al. (2021). Genetic mutation and biological pathway prediction based on whole slide images in breast carcinoma using deep learning. *Npj Precision Oncology* 5. doi:ARTN8710.1038/s41698-021-00225-9

[99]Wang, X. X., Zou, C., Zhang, Y., Li, X. Q., Wang, C. X., Ke, F., et al. (2021). Prediction of brca gene mutation in breast cancer based on deep learning and histopathology images. *Frontiers in Genetics* 12. doi:ARTN66110910.3389/fgene.2021.661109

[100]Arya, N. and Saha, S. (2021). Multi-modal advanced deep learning architectures for breast cancer survival prediction. *Knowledge-Based Systems* 221. doi:ARTN10696510.1016/j.knosys.2021.106965

[101]Yamashita, R., Long, J., Longacre, T., Peng, L., Berry, G., Martin, B., et al. (2021). Deep learning model for the prediction of microsatellite instability in colorectal cancer: a diagnostic study. *Lancet Oncology* 22, 132–141

[102]Klein, S., Quaas, A., Quantius, J., Loser, H., Meinel, J., Peifer, M., et al. (2021). Deep learning predicts hpv association in oropharyngeal squamous cell carcinomas and identifies patients with a favorable prognosis using regular h&e stains. *Clinical Cancer Research* 27, 1131–1138. doi:10.1158/1078-0432.Ccr-20-3596

[103]Wang, X. D., Chen, Y., Gao, Y. S., Zhang, H. Q., Guan, Z. H., Dong, Z., et al. (2021). Predicting gastric cancer outcome from resected lymph node histopathology images using deep learning. *Nature Communications* 12. doi:ARTN163710.1038/s41467-021-21674-7

[104]Shi, J. Y., Wang, X., Ding, G. Y., Dong, Z., Han, J., Guan, Z., et al. (2021). Exploring prognostic indicators in the pathological images of hepatocellular carcinoma based on deep learning. *Gut* 70, 951–961. doi:10.1136/gutjnl-2020-320930

[105]Wulczyn, E., Steiner, D. F., Xu, Z. Y., Sadhwani, A., Wang, H. W., Flament-Auvigne, I., et al. (2020). Deep learning-based survival prediction for multiple cancer types using histopathology images. *Plos One* 15. doi:ARTNe023367810.1371/journal.pone.0233678

[106]Chen, R. J., Lu, M. Y., Wang, J., Williamson, D. F. K., Rodig, S. J., Lindeman, N. I., et al. (2020). Pathomic fusion: An integrated framework for fusing histopathology and genomic features for cancer diagnosis and prognosis. *IEEE Trans Med Imaging* PP. doi:10.1109/TMI.2020.3021387

[107]Guo, Y., Wang, Q., Guo, Y., Zhang, Y. Y., Fu, Y., and Zhang, H. M. (2021). Preoperative prediction of perineural invasion with multi-modality radiomics in rectal cancer. *Scientific Reports* 11. doi:ARTN942910.1038/s41598-021-88831-2

[108]Khan, M. A., Ashraf, I., Alhaisoni, M., Damasevicius, R., Scherer, R., Rehman, A., et al. (2020). Multimodal brain tumor classification using deep learning

and robust feature selection: A machine learning application for radiologists. *Diagnostics* 10. doi:ARTN56510.3390/diagnostics10080565

[109]Wu, J., Li, C., Gensheimer, M., Padda, S., Kato, F., Shirato, H., et al. (2021). Radiological tumour classification across imaging modality and histology. *Nature Machine Intelligence* 3, 787–+. doi:10.1038/s42256-021-00377-0

[110]Zhao, J. F., Li, D. W., Xiao, X. J., Accorsi, F., Marshall, H., Cossetto, T., et al. (2021). United adversarial learning for liver tumor segmentation and detection of multi-modality non-contrast mri. *Medical Image Analysis* 73. doi:ARTN10215410.1016/j.media.2021.102154

[111]Alvarez-Jimenez, C., Sandino, A. A., Prasanna, P., Gupta, A., Viswanath, S. E., and Romero, E. (2020). Identifying cross-scale associations between radiomic and pathomic signatures of non-small cell lung cancer subtypes: Preliminary results. *Cancers* 12. doi:ARTN366310.3390/cancers12123663

[112]Giardina, G., Micko, A., Bovenkamp, D., Krause, A., Placzek, F., Papp, L., et al. (2021). Morpho-molecular metabolic analysis and classification of human pituitary gland and adenoma biopsies based on multimodal optical imaging. *Cancers* 13. doi:ARTN323410.3390/cancers13133234

[113]Zhou, X. C., Lin, Q. M., Gui, Y. Y., Wang, Z. X., Liu, M. H., and Lu, H. (2021). Multimodal mr images-based diagnosis of early adolescent attention-deficit/hyperactivity disorder using multiple kernel learning. *Frontiers in Neuroscience* 15. doi:ARTN71013310.3389/fnins.2021.710133

[114]Calisto, F. M., Santiago, C., Nunes, N., and Nascimento, J. C. (2021). Introduction of human-centric ai assistant to aid radiologists for multimodal breast image classification. *International Journal of Human-Computer Studies* 150. doi:ARTN10260710.1016/j.ijhcs.2021.102607

[115]Joo, S., Ko, E. S., Kwon, S., Jeon, E., Jung, H., Kim, J. Y., et al. (2021). Multimodal deep learning models for the prediction of pathologic response to neoadjuvant chemotherapy in breast cancer. *Scientific Reports* 11. doi: ARTN1880010.1038/s41598-021-98408-8

[116]Yang, Y., Yang, J. C., Shen, L., Chen, J. J., Xia, L. L., Ni, B. B., et al. (2021). A multi-omics-based serial deep learning approach to predict clinical outcomes of single-agent anti-pd-1/pd-l1 immunotherapy in advanced stage non-small-cell lung cancer. *American Journal of Translational Research* 13, 743–+

[117]Yan, J. L., Toh, C. H., Ko, L., Wei, K. C., and Chen, P. Y. (2021). A neural network approach to identify glioblastoma progression phenotype from multimodal mri. *Cancers* 13. doi:ARTN200610.3390/cancers13092006

[118]Mariscotti, G., Durando, M., Tagliafico, A., Campanino, P. P., Bosco, D., Casella, C., et al. (2020). Preoperative breast cancer staging with multi-modality imaging and surgical outcomes. *European Journal of Radiology* 122. doi: ARTN10876610.1016/j.ejrad.2019.108766

[119]Rossi, L., Bijman, R., Schillemans, W., Aluwini, S., Cavedon, C., Witte, M., et al. (2018). Texture analysis of 3d dose distributions for predictive modelling of toxicity rates in radiotherapy. *Radiotherapy and Oncology* 129, 548–553. doi:10.1016/j.radonc.2018.07.027

[120]Gabrys, H. S., Buettner, F., Sterzing, F., Hauswald, H., and Bangert, M. (2018). Design and selection of machine learning methods using radiomics and dosiomics for normal tissue complication probability modeling of xerostomia. *Frontiers in Oncology* 8. doi:ARTN3510.3389/fonc.2018.00035

[121]Liang, B., Yan, H., Tian, Y., Chen, X. Y., Yan, L. L., Zhang, T., et al. (2019). Dosiomics: Extracting 3d spatial features from dose distribution to predict incidence of radiation pneumonitis. *Frontiers in Oncology* 9. doi:ARTN26910.3389/fonc.2019.00269

[122]Adachi, T., Nakamura, M., Shintani, T., Mitsuyoshi, T., Kakino, R., Ogata, T., et al. (2021). Multi-institutional dose-segmented dosiomic analysis for predicting radiation pneumonitis after lung stereotactic body radiation therapy. *Medical Physics* 48, 1781–1791. doi:10.1002/mp.14769

[123]Lee, S. H., Han, P. J., Hales, R. K., Voong, K. R., Noro, K., Sugiyama, S., et al. (2020). Multi-view radiomics and dosiomics analysis with machine learning for predicting acute-phase weight loss in lung cancer patients treated with radiotherapy. *Physics in Medicine and Biology* 65. doi:ARTN19501510.1088/1361-6560/ab8531

[124]Liang, B., Van, Y., Chen, X. Y., Yan, H., Yan, L. L., Zhang, T., et al. (2020). Prediction of radiation pneumonitis with dose distribution: A convolutional neural network (cnn) based model. *Frontiers in Oncology* 9. doi:ARTN150010.3389/fonc.2019.01500

[125]Wu, A. Q., Li, Y. B., Qi, M. K., Lu, X. Y., Jia, Q. Y., Guo, F. T., et al. (2020). Dosiomics improves prediction of locoregional recurrence for intensity modulated radiotherapy treated head and neck cancer cases. *Oral Oncology* 104. doi:ARTN10462510.1016/j.oraloncology.2020.104625

[126]Murakami, Y., Soyano, T., Kozuka, T., Ushijima, M., Koizumi, Y., Miyauchi, H., et al. (2021). Dose-based radiomic analysis (dosiomics) for intensity-modulated radiotherapy in patients with prostate cancer: Correlation between planned dose distribution and biochemical failure. *International journal of radiation oncology, biology, physics* doi:10.1016/j.ijrobp.2021.07.1714

[127]Buizza, G., Paganelli, C., D'Ippolito, E., Fontana, G., Molinelli, S., Preda, L., et al. (2021). Radiomics and dosiomics for predicting local control after carbon-ion radiotherapy in skull-base chordoma. *Cancers* 13. doi:ARTN33910.3390/cancers13020339

[128]Hirashima, H., Ono, T., Nakamura, M., Miyabe, Y., Mukumoto, N., Iramina, H., et al. (2020). Improvement of prediction and classification performance for gamma passing rate by using plan complexity and dosiomics features. *Radiotherapy and Oncology* 153, 250–257. doi:10.1016/j.radonc.2020.07.031

[129]Placidi, L., Gioscio, E., Garibaldi, C., Rancati, T., Fanizzi, A., Maestri, D., et al. (2021). A multicentre evaluation of dosiomics features reproducibility, stability and sensitivity. *Cancers* 13. doi:ARTN383510.3390/cancers13153835
